# A new mass spectral library for high-coverage and reproducible analysis of the *Plasmodium falciparum*–infected red blood cell proteome

**DOI:** 10.1093/gigascience/giac008

**Published:** 2022-03-07

**Authors:** Ghizal Siddiqui, Amanda De Paoli, Christopher A MacRaild, Anna E Sexton, Coralie Boulet, Anup D Shah, Mitchell B Batty, Ralf B Schittenhelm, Teresa G Carvalho, Darren J Creek

**Affiliations:** Drug Delivery Disposition and Dynamics, Monash Institute of Pharmaceutical Sciences, Monash University, Parkville, VIC 3052, Australia; Drug Delivery Disposition and Dynamics, Monash Institute of Pharmaceutical Sciences, Monash University, Parkville, VIC 3052, Australia; Drug Delivery Disposition and Dynamics, Monash Institute of Pharmaceutical Sciences, Monash University, Parkville, VIC 3052, Australia; Drug Delivery Disposition and Dynamics, Monash Institute of Pharmaceutical Sciences, Monash University, Parkville, VIC 3052, Australia; Department of Physiology, Anatomy and Microbiology, La Trobe University, Bundoora, VIC 3086, Australia; Department of Biochemistry and Molecular Biology, Biomedicine Discovery Institute, Monash University, Clayton, VIC 3800, Australia; Monash Bioinformatics Platform, Biomedicine Discovery Institute, Monash University, Clayton, VIC 3800, Australia; Drug Delivery Disposition and Dynamics, Monash Institute of Pharmaceutical Sciences, Monash University, Parkville, VIC 3052, Australia; Department of Biochemistry and Molecular Biology, Biomedicine Discovery Institute, Monash University, Clayton, VIC 3800, Australia; Department of Physiology, Anatomy and Microbiology, La Trobe University, Bundoora, VIC 3086, Australia; Drug Delivery Disposition and Dynamics, Monash Institute of Pharmaceutical Sciences, Monash University, Parkville, VIC 3052, Australia

**Keywords:** Plasmodium falciparum, malaria, proteomics, data-dependent acquisition, data-independent acquisition, red blood cells, LC-MS/MS

## Abstract

**Background:**

*Plasmodium falciparum* causes the majority of malaria mortality worldwide, and the disease occurs during the asexual red blood cell (RBC) stage of infection. In the absence of an effective and available vaccine, and with increasing drug resistance, asexual RBC stage parasites are an important research focus. In recent years, mass spectrometry–based proteomics using data-dependent acquisition has been extensively used to understand the biochemical processes within the parasite. However, data-dependent acquisition is problematic for the detection of low-abundance proteins and proteome coverage and has poor run-to-run reproducibility.

**Results:**

Here, we present a comprehensive *P. falciparum*–infected RBC (iRBC) spectral library to measure the abundance of 44,449 peptides from 3,113 *P. falciparum* and 1,617 RBC proteins using a data-independent acquisition mass spectrometric approach. The spectral library includes proteins expressed in the 3 morphologically distinct RBC stages (ring, trophozoite, schizont), the RBC compartment of trophozoite-iRBCs, and the cytosolic fraction from uninfected RBCs. This spectral library contains 87% of all *P. falciparum* proteins that have previously been reported with protein-level evidence in blood stages, as well as 692 previously unidentified proteins. The *P. falciparum* spectral library was successfully applied to generate semi-quantitative proteomics datasets that characterize the 3 distinct asexual parasite stages in RBCs, and compared artemisinin-resistant (Cam3.II^R539T^) and artemisinin-sensitive (Cam3.II^rev^) parasites.

**Conclusion:**

A reproducible, high-coverage proteomics spectral library and analysis method has been generated for investigating sets of proteins expressed in the iRBC stage of *P. falciparum* malaria. This will provide a foundation for an improved understanding of parasite biology, pathogenesis, drug mechanisms, and vaccine candidate discovery for malaria.

## Background

Malaria, a mosquito-borne disease caused by *Plasmodium* parasites, is endemic in countries of Southeast Asia, Latin America, and the sub-Saharan regions of Africa. *Plasmodium falciparum* is the most lethal of human *Plasmodium* parasites [1]. *P. falciparum* has a complex life cycle characterized by distinctive morphological changes that span the human and mosquito host, with each stage of development supported by versatile biological processes that allow the parasite to adapt to multiple host environments. However, disease outcomes associated with this disease are entirely attributed to the proliferative asexual development within human red blood cells (RBCs). During a single replication cycle within the RBCs, parasites undergo pronounced changes over a period of 48 hours, which can be roughly divided into 3 stages: rings (0–20 hours post invasion [h.p.i.]), trophozoites (22–38 h.p.i.), and schizonts (40–48 h.p.i.). The latter stage results in up to 32 daughter merozoites rupturing from a single infected cell, re-entering circulation, and propagating the infection [2–4]. Because of the disease conditions associated with these RBC stages, it is not surprising that most treatment efforts focus on this stage.

The global endeavour towards eradication of malaria would be greatly enhanced by access to an effective and affordable vaccine. However, with no such vaccine yet available, prevention and treatment approaches will continue to rely on the use of vector control strategies and chemotherapeutics. Furthermore, eradication from endemic regions is hampered by the emergence of drug resistance to frontline artemisinin-based therapies and partner drugs in the Greater Mekong region of Southeast Asia [5, 6] and, more recently and perhaps most troubling, the emergence of *de novo* artemisinin resistance in Africa [7]. Collectively, this drug-resistance hampers the global progress towards elimination goals and highlights the urgent need for a greater understanding of *P. falciparum* biochemistry to underpin the discovery of new medicines, diagnostics and vaccines.

In recent years, large-scale quantitative proteomics has facilitated the accurate identification of proteins in complex mixtures, examination of alterations in protein expression and abundance, and probing the composition of protein-protein complexes. While other system-wide methods for molecular analysis of *P. falciparum*, such as transcriptomics, have been useful for identifying unique sets of potential molecular targets, messenger RNA (mRNA) expression often poorly correlates with protein abundance [8–12]. Therefore, direct measurement of protein abundance provides a better representation of the parasite phenotype under given study conditions compared to transcript levels.

Liquid chromatography tandem mass spectrometry (LC-MS/MS)-based quantitative proteomics is the method of choice to measure dynamic changes in global protein levels across biological samples. Data-dependent acquisition (DDA) has been extensively used to understand the scope of protein changes across different conditions, whereby the top 10 or 20 precursor MS1 ions detected by mass spectrometry (MS) for each scan are fragmented to give product ion spectra (MS2), which provides a fingerprint for peptide detection and identification [13, 14]. However, the stochastic nature of precursor selection for fragmentation leads to inconsistencies and variations in peptide identification between replicates and samples. This becomes particularly problematic for low-abundance peptides and reduces the number of proteins that can be accurately quantified [15, 16]. Data-independent acquisition (DIA) is gaining popularity as an alternative data collection method, as MS2 spectra are collected for multiple peptides within a predefined mass-to-charge (*m/z*) range by co-isolating and fragmenting all peptide precursors within the *m/z* range at once [17]. Although the *m/z* range definition may still exclude some peptide populations, DIA ultimately results in extremely high run-to-run reproducibility and a more comprehensive dataset over a shorter time period, making it superior to DDA [17–21].

The primary approach for DIA analysis requires prior knowledge of peptide fragmentation stored in spectral ion libraries. Furthermore, as library quality directly influences DIA results, it is important to have a comprehensive and in-depth library that accurately represents the proteome of the organism under investigation [17, 18]. Comprehensive spectral libraries are available for many model organisms [19–21]. However, no such library exists for *P. falciparum*, although such a tool would provide multifaceted support for the identification of much-needed drug targets and vaccine candidates. In this study, we produced a comprehensive library obtained from *P. falciparum–*infected RBCs (iRBCs), identifying 3,113 parasite and 1,617 RBC proteins. The spectral library combined with the DIA-MS method was used to perform quantitative analyses of the 3 distinct asexual RBC stages of the 3D7 wild-type reference line, and the trophozoite stage of artemisinin-resistant (Cam3.II^R539T^ and Cam3.II^C580Y^) and artemisinin-sensitive (Cam3.II^rev^) lines. The comprehensive library generated in this study combined with the DIA methodology provides an exquisite and valuable resource to address basic biological questions. Drug mode-of-action studies and the identification of novel therapeutic targets, as well as studies aimed at identifying vaccine antigens and diagnostic markers of *P. falciparum* infection, will also benefit from this resource.

## Data Description

Given that nearly half of the world's population is at risk of contracting malaria, an in-depth understanding of the proteins involved in disease onset and progression, and how their expression, structure, and function are responsible for disease pathology in the iRBC stage, is critical. Technical advances in proteomics for malaria, such as DIA, are required to fully identify and quantify the entire complement of proteins. DIA analysis requires access to large and comprehensive proteomic datasets. To address these gaps, we generated a comprehensive spectral library from highly synchronized asexual ring-, trophozoite-, and schizont-stage parasites of the *P. falciparum* 3D7 reference strain, including the cytosolic fractions from uninfected RBCs (uRBCs) and the infected-RBC cytosol (where the parasite exports a large number of its proteins [22]) of trophozoite-iRBCs. Parasites were released from the host RBC using 0.1% saponin, centrifuged, and collected as pellets for downstream protein extraction (saponin pellet). The saponin supernatant, containing the soluble cytosolic contents from the host RBC, were incubated with TALON® Metal Affinity Resin to remove haemoglobin prior to protein extraction. Proteins from both the cytosolic and parasite fractions were solubilized and subjected to proteolysis with trypsin. The digested samples were then extensively fractionated using SCX Bond Elut Plexa cartridges and analysed using nanoLC-MS/MS. The raw data were analysed using MaxQuant to generate the spectral library, identifying 3,113 parasite and 1,617 RBC proteins, which was then incorporated into Spectronaut for further application. The spectral library covered 87% of all proteins previously detected in the *P. falciparum* blood stages by MS and added a further 692 proteins that were not previously reported with detection at the protein level in asexual *P. falciparum* parasites.

The spectral library was successfully applied for quantitative analysis of *P. falciparum* proteins. We demonstrated a capacity to quantify 2,063 *P. falciparum* proteins with nearly no missing proteins across ring, trophozoite, and schizont stages of infection using the DIA method. Subsequent enrichment analysis highlighted a plethora of stage-specific functional diversity across blood-stage development. We also used the spectral library to compare 2,317 *P. falciparum* proteins between artemisinin-resistant (Cam3.II^R539T^ and Cam3.II^C580Y^) and artemisinin-sensitive (Cam3.II^rev^) parasites [23]. We confirmed our previous DDA-based quantitative proteomics analysis of these lines [24] and further identified a number of additional parasite proteins enriched in specific dysregulated pathways. Table 1 shows the parasite stage, including hours post RBC invasion, and the number of parasites used for quantitative analysis. This analysis confirmed that the spectral library generated in this work, accompanied by the DIA methodology, can successfully perform reproducible, specific, and accurate quantitative proteomics of *P. falciparum* asexual RBC stages and can be used to investigate a wide range of biological questions. Furthermore, the comparative DIA studies will be available as datasets in PlasmoDB [25] to act as reference databases for the worldwide community of malaria researchers.

**Table 1:** Parasites used for quantitative proteomics applying the *P. falciparum* spectral library and DIA-MS methodology

Parasite line	Stage	No. of parasites used	Normalized on protein amount (µg)	Protein ratio (µg/1 mL of parasites)
3D7 reference strain	Ring (6–12 h.p.i.)	16% Parasitaemia, 2% HCT, 45 mL culture	500	69
3D7 reference strain	Trophozoite (22–28 h.p.i.)	8% Parasitaemia, 2% HCT, 15 mL culture	500	417
3D7 reference strain	Schizont (38–42 h.p.i.)	8% Parasitaemia, 2% HCT, 15 mL culture	500	417
Cam3.II^R539T^ artemisinin resistant [23]	Trophozoite (22–26 h.p.i.)	8% Parasitaemia, 2% HCT, 15 mL culture	500	417
Cam3.II^rev^ artemisinin sensitive [23]	Trophozoite (22–26 h.p.i.)	8% Parasitaemia, 2% HCT, 15 mL culture	500	417
Cam3.II^C580Y^ artemisinin resistant [23]	Trophozoite (22–26 h.p.i.)	8% Parasitaemia, 2% HCT, 15 mL culture	500	417

HCT: haematocrit; h.p.i.: hours post invasion.

## Analyses

### Generation of DDA library

**Figure 1:**
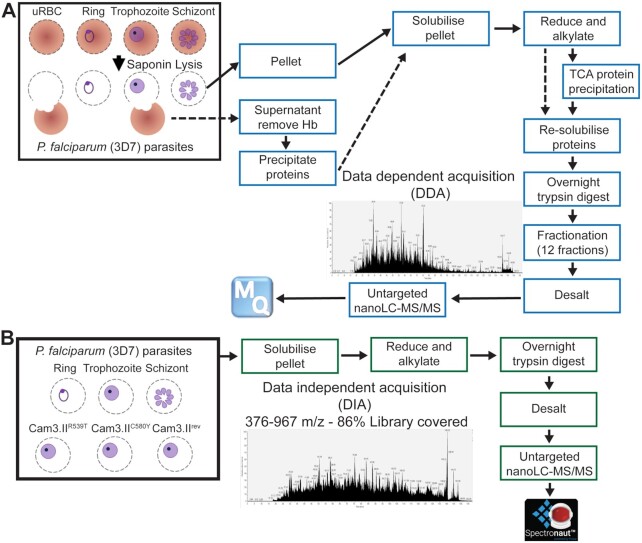
Flow chart for creation and application of the spectral library for *Plasmodium falciparum*–infected red blood cells. (A) The spectral library (blue boxes) was built from uninfected red blood cells (uRBC), ring-, trophozoite-, and schizont-stage (*P. falciparum* 3D7) parasites, where both the saponin pellets (solid arrows) and supernatants (dashed arrows) were combined (3–5 mg of proteins), trypsinized, fractionated extensively, and data analysed using Maxquant. (B) The spectral library was then used to analyse samples (green boxes) from *P. falciparum* 3D7 parasites (ring-, trophozoite-, and schizont-stage) and artemisinin-resistant (Cam3.II^R539T^, Cam3.II^C580Y^) and artemisinin-sensitive (Cam3.II^rev^) parasites (saponin pellets only, 500 µg of proteins) over a window of 376–967 *m/z* covering 86% of the spectral library generated. The data were analysed using Spectronaut^TM^. Hb: haemoglobin.

Here we present a comprehensive *P. falciparum* asexual RBC stage spectral library to support protein quantification by DIA-MS. The library was generated by combining 56 DDA analyses of peptide samples derived from asexual ring-, trophozoite-, and schizont-stage parasites (parasite pellets) and the cytosolic fractions from uRBCs and the RBC compartment of trophozoite-iRBCs. For the cytosolic fractions, haemoglobin was removed using TALON® Resin prior to protein precipitation using trichloroacetic acid (TCA) and further solubilization as per Fig. 1. The DDA data were collected through extensive peptide fractionation using SCX cartridges and analysed using untargeted nanoLC-MS/MS with reversed phase chromatography and high-resolution (Orbitrap) MS. The library was generated using MaxQuant (Fig. 1). The *P. falciparum* asexual RBC stage spectral library identified 44,449 proteotypic peptides, which mapped to 3,113 *P. falciparum* proteins and 1,617 human RBC proteins (Supplementary Data S1).

### Properties of the *P. falciparum* asexual RBC stage spectral library

**Figure 2:**
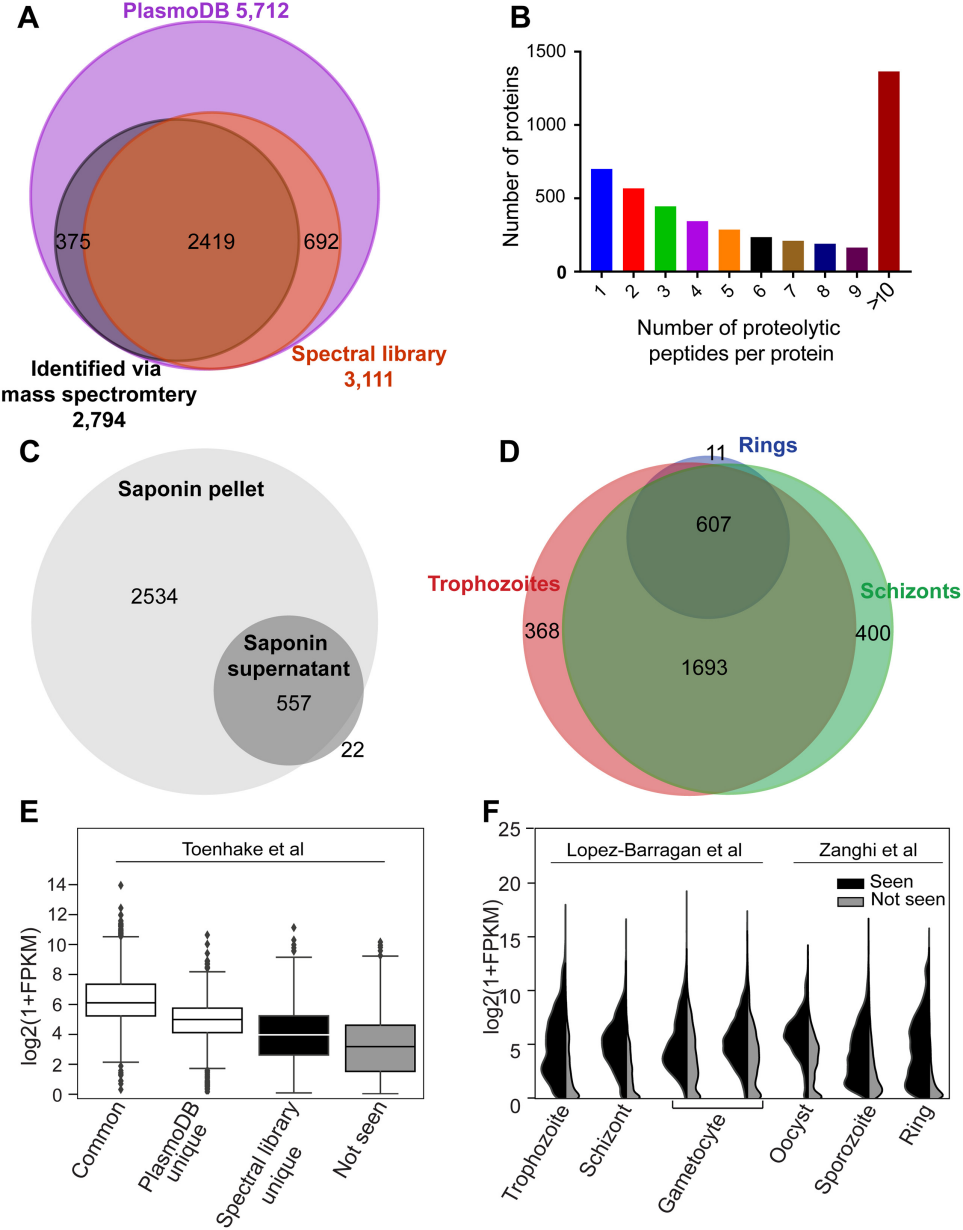
Characterization of *Plasmodium falciparum* asexual red blood cell stage spectral library. (A) Venn diagram describing overlap of identified *P. falciparum* proteins from the generated spectral library (orange) with *P. falciparum* protein-encoding genes in PlasmoDB (purple), and PlasmoDB subset annotated with protein-level evidence from mass spectrometry (black). (B) The number of proteotypic peptides per protein in the *P. falciparum* spectral library. (C) Venn diagram of identified *P. falciparum* proteins for spectral library generation from saponin soluble and insoluble fractions, and (D) of ring-, trophozoite-, and schizont-stage parasites. (E) Box plot of FPKM distribution of transcript expression level from Toenhake et al. [26] dataset for genes common to spectral library and PlasmoDB (common), unique to PlasmoDB and spectral library, and not identified (not seen) with logarithmic values of FPKM on the vertical axis. Boxes show the median and inter-quartile range and whiskers extend 1.5 times the IQR beyond each quartile. Values beyond this range are shown as individual points. (F) Violin plots displaying the expression intensity distribution of the genes from Lopez-Barragan et al. [27] and Zanghi et al. [28] datasets with products identified (seen, left) and not identified (not seen, right) in the *P. falciparum* spectral library across the distinct life cycle stages.

To demonstrate the proteome coverage of the *P. falciparum* asexual stage spectral library, we compared the proteins included in this library with those in the online reference database, PlasmoDB (release 51) [25] (5,712 protein-encoding genes based on the current genome annotation), and those with evidence of prior detection at the protein level (2,792 protein groups) based on mass spectrometric approaches in the asexual RBC stage [25]. We report that 2,419 of the 3,113 proteins identified in this library have been detected previously, representing 87% of genes previously annotated with evidence of protein-level expression in *P. falciparum*. Importantly, the remaining 692 proteins identified in this library have not been reported previously with MS evidence of protein-level expression in asexual stage development (Supplementary Data S2) (Fig. 2A). Among these 692 additional proteins, 210 (30%) were identified with 1 unique peptide, while the rest were identified with ≥2 unique peptides. The single-hit peptides had high-quality MS2 spectra and the same peptide was seen 15 times, on average, in the DDA dataset (minimum 4 times). Furthermore, to show the coverage of each protein detected in the entire library, we calculated the number of proteotypic peptides observed per protein. Roughly 30% of the proteins in the library contained >10 proteotypic peptides, and 85% of these contained ≥2 proteotypic peptides per protein (Supplementary Data S3) (Fig. 2B). Further analysis of proteins identified in this spectral library revealed 579 *P. falciparum* proteins identified from the saponin supernatant of iRBCs, of which only 22 proteins were specific to the RBC cytosol of the iRBCs and not detected in parasite cell pellets (Fig. 2C). Of the 3,091 *P. falciparum* proteins identified from the parasite pellet, 630 came from the ring-stage fraction, 2,674 from the trophozoite-stage fraction, and 2,706 from the schizont-stage fraction, with 607 proteins common between all 3 stages (Fig. 2D). For the uRBC proteome, we identified a total of 1,617 proteins, of which 408 were soluble in the saponin lysate, 478 overlapped between soluble and insoluble fractions, and 731 were unique to the insoluble fraction (Supplementary Data S1 and Supplementary Fig. S1).

It is important to assess the characteristics of this library in comparison to what has been previously published for *P. falciparum* to ensure that it is well suited to relevant studies of parasite biology. Therefore, we analysed proteins identified in this spectral library with respect to published mRNA expression (Fig. 2E). We analysed 4 classes of parasite proteins: those in this library and with prior MS-level evidence in asexual blood stages documented in PlasmoDB (common); proteins absent from this library but with protein-level evidence in PlasmoDB (PlasmoDB unique); proteins in this library that had not previously been identified in asexual RBC stages (library unique); and predicted parasite proteins that are not identified either in this library or in any published asexual RBC proteomics dataset (not seen) (Fig. 2E) [26]. Proteins commonly seen had the highest transcript abundance based on normalized RNAseq counts (FPKM), while proteins unique to either PlasmoDB or this library had lower transcript abundance (Fig. 2E). Furthermore, proteins that lacked proteomic-level evidence in asexual stages had the lowest transcript abundance, suggesting that this basal level of transcription does not necessitate translation of detectable amounts of this protein subset in asexual stages, and that expression levels may be higher in other stages of the *P. falciparum* lifecycle. To address this hypothesis, we compared the protein expression of identified (seen) and unidentified (not seen) proteins to previously published mRNA expression data from sexual stages in blood (gametocytes) and mosquito (oocyst and sporozoite). Our results indicate that proteins “not seen” had higher transcript abundance in gametocytes, oocyst, and sporozoites compared to asexual stages (Fig. 2F) [27, 28], supporting the hypothesis that undetected proteins with low-level mRNA transcription in asexual stages likely have a specific function in sexual stage parasite development. Our study provides clear evidence that ∼50% of *P. falciparum* genes are expressed during asexual RBC stages, while the other 50% have a primary function in other life cycle stages.

### Accuracy of the asexual spectral library using DIA-MS methodology

**Figure 3:**
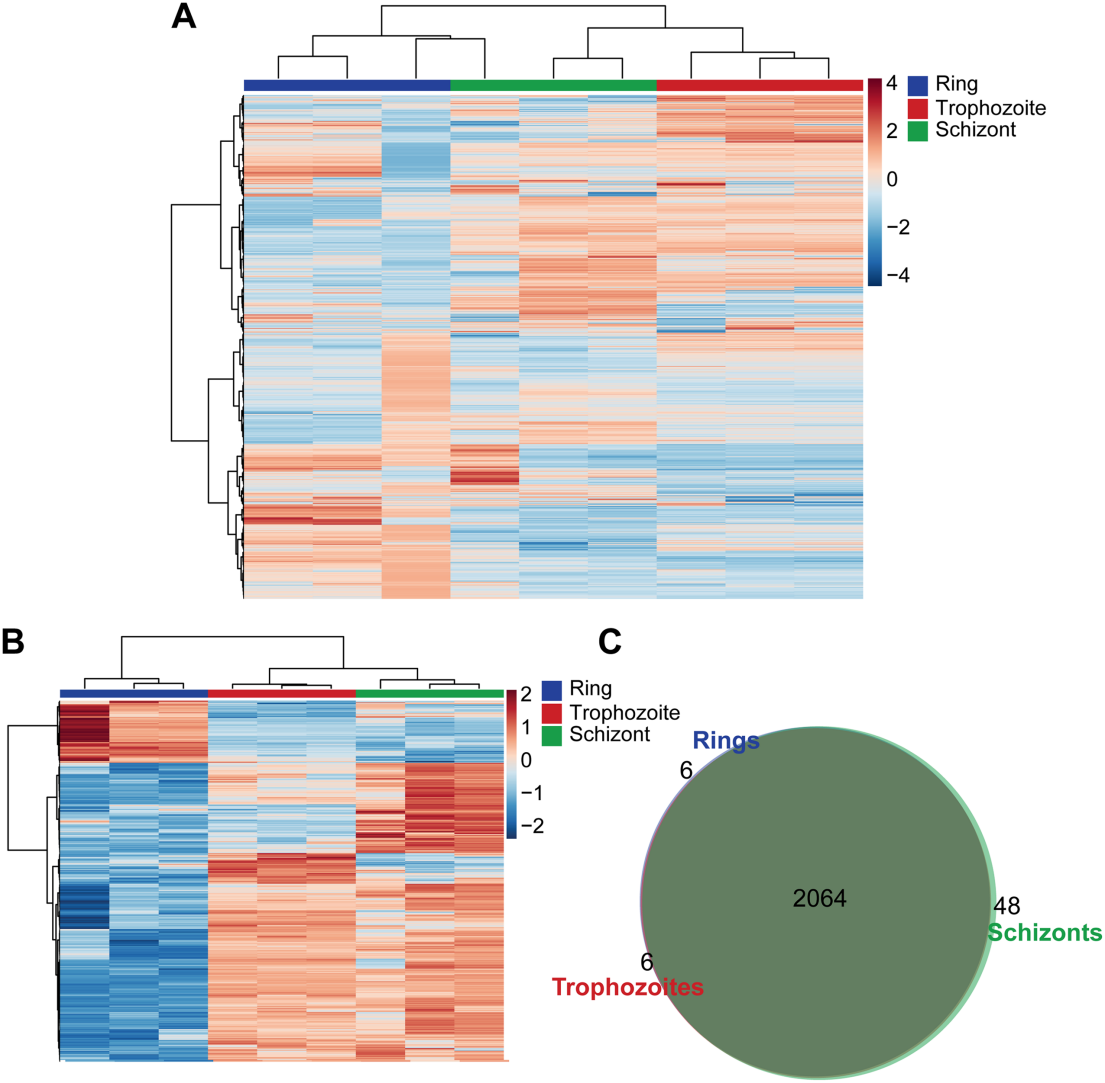
Hierarchical clustering of *P. falciparum* proteins of ring-, trophozoite-, and schizont-stage parasites. Vertical clustering displays similarities between sample groups, while horizontal clusters reveal the relative abundances of all identified proteins (2,064) (A) and the 400 (B) most significantly different proteins from DIA analysis. (C) Venn diagram of identified *P. falciparum* proteins in ring-, trophozoite-, and schizont-stage parasites using the DIA-MS. Data shown are from 3 independent biological replicates. The colour scale bar represents log_2_ (mean-centered and divided by the standard deviation of each variable) intensity values.

To show the application of the *P. falciparum* spectral library using DIA-MS, we prepared ring-, trophozoite-, and schizont-stage parasites with a minimum of 3 biological replicates in Experiment 1 and 2–3 biological replicates in Experiment 2 (Fig. 3 and Supplementary Fig. S2). Raw DIA files were loaded into Spectronaut^TM^ and processed with its associated default workflow for peptide identification against our spectral library, followed by protein normalization and quantification. In total, >2,000 proteins (2,064 proteins for Experiment 1 [Supplementary Data S4]; 2,120 proteins for Experiment 2 [Supplementary Data S5]) were quantified in each of the distinct stages of the parasite using the *P. falciparum* library (Fig. 3 and Supplementary Fig. S2). For comparison, the raw DIA files from Experiment 1 were also analysed using a spectral library–free DIA “DirectDIA” approach, which identified a significantly lower number of proteotypic peptides (14,639 peptides) than the spectral library search (33,731 peptides), resulting in increased coefficient of variation (CV) between samples. This improved precision and proteome coverage from spectral library–based DIA compared to a library-free approach has been observed in previous studies [29, 30]. The DirectDIA approach was therefore not used for identification and quantification of proteins.

**Figure 4:**
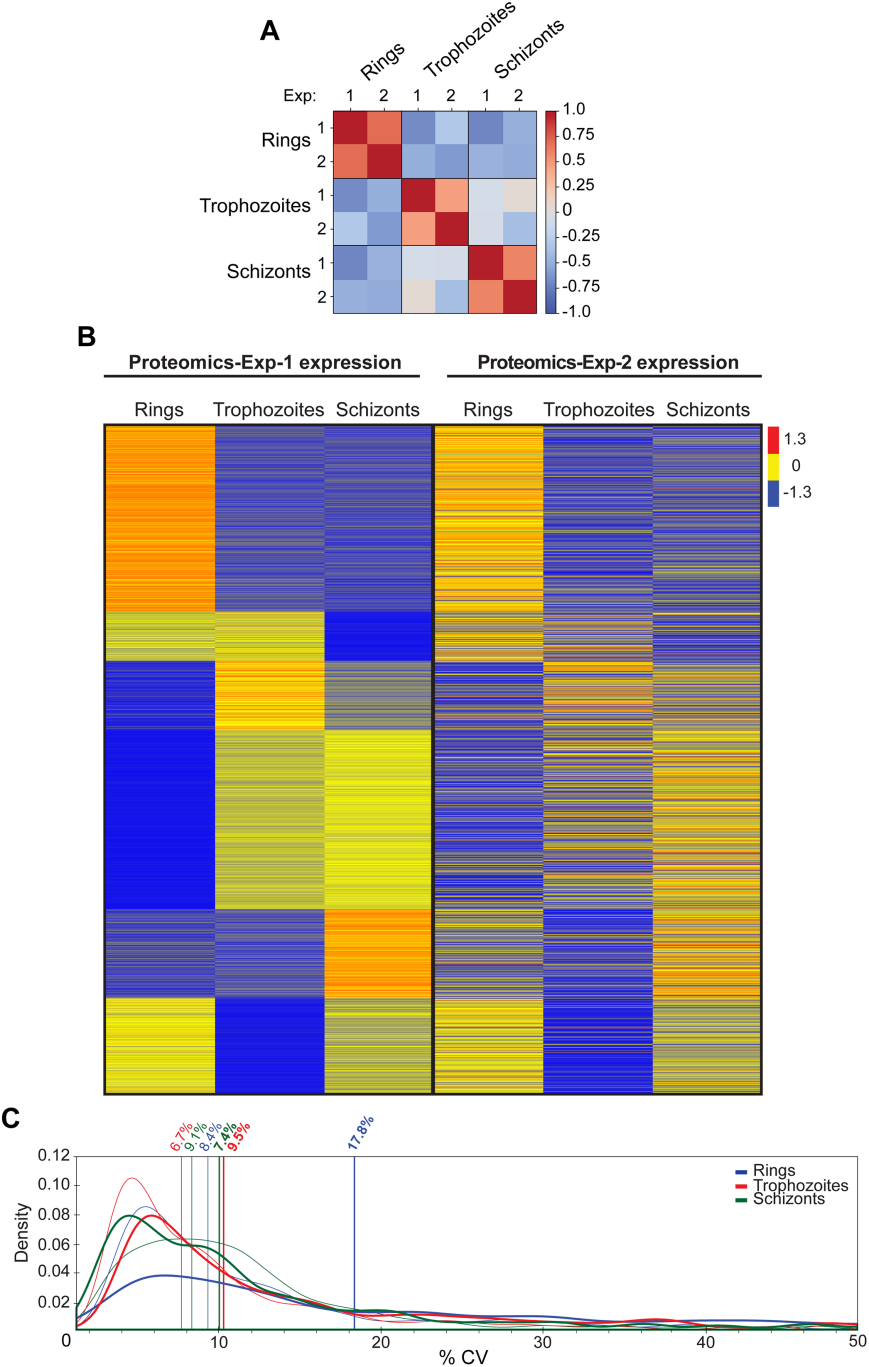
Analysing stage-specific DIA-MS data using the *P. falciparum* spectral library. (A) Pearson correlation of mean protein intensities in each specific stage (ring-, trophozoite,- and schizont-stage parasites) identified in Experiments 1 and 2. (B) Heat map of the *P. falciparum* mean protein expression patterns of 1,990 common proteins from ring- to trophozoite- to schizont-stage parasites in Experiments 1 and 2. *Left:* proteomics data (Experiment 1); *right:* independent proteomics dataset (Experiment 2). The colour code is as follows: red indicates up-regulated proteins; blue indicates down-regulated proteins; yellow indicates unchanged proteins. (C) Coefficient of variation (%CV) of protein intensities in Experiment 1 (n = 3) and Experiment 2 (n = 2–3) for each of the distinct stages. Bold lines show Experiment 2. Vertical lines indicate median %CV for each stage in each experiment.

To evaluate the quality of the *P. falciparum* library–based analysis, we compared protein abundances of 1,990 common proteins (proteins without missing values) across the 2 different experiments (Supplementary Data S6 and Fig. 4). Pearson correlation coefficients between Experiments 1 and 2 were 0.7 for rings, 0.5 for trophozoites, and 0.6 for schizonts (Fig. 4A). Heat map analysis of protein expression across the 3 stages for the 2 experiments indicated that the expression patterns of the proteins are reproducible (Fig. 4B). To further determine quantitative reproducibility, we computed the CV in each specific asexual stage across both experiments. For all asexual specific stages, the median CVs of protein abundances were <10% (Fig. 4C) with the exception of the ring-stage sample in Experiment 2 (CV = 17.8%). Collectively, the *P. falciparum* asexual library–based analysis using our DIA-MS exhibited excellent reproducibility.

**Figure 5:**
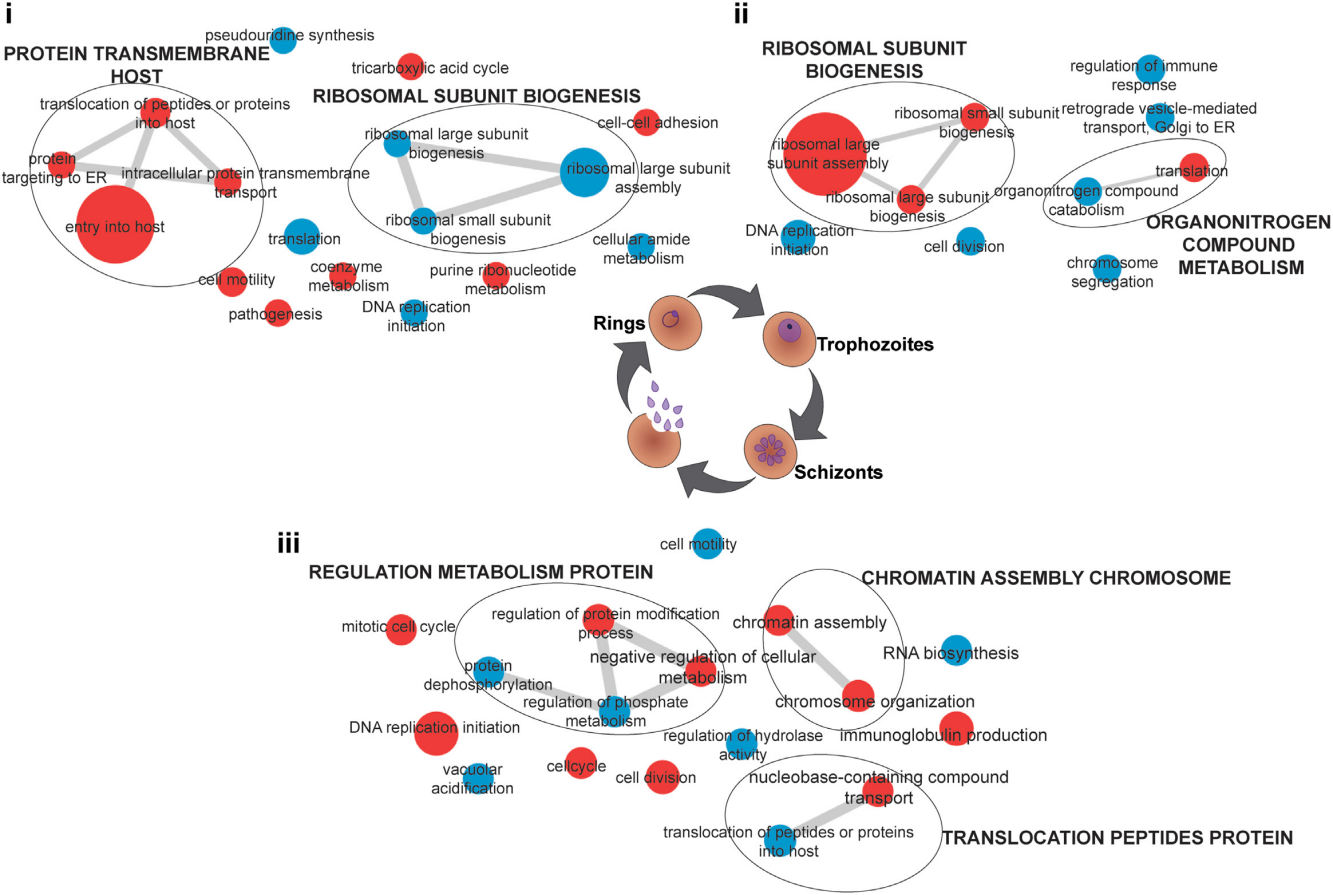
Gene Ontology network analysis of processes enriched in the spectral library for each stage of asexual development. Gene Ontology (GO) terms significantly overrepresented in the transition between the 3 distinct stages of the asexual development (**i**, schizont- to ring-stage parasites; **ii**, ring- to trophozoite-stage parasites; **iii**, trophozoite- to schizont-stage parasites) were obtained using in-house methods and imported into REVIGO for network compilation. Visualization of network enrichment was performed using Cytoscape (v3.8.2). Each node is sized by increasing GO term uniqueness and coloured according whether the process is up (red) or down (blue) by comparison to the subsequent developmental stage. Grey bars connecting each node represent gene overlap between terms.

Hierarchical clustering confirms the good agreement between biological replicates and shows a clear distinction of expression pattern between the 3 asexual stages (Fig. 3, Supplementary Figs S2 and S3). Heat map analysis shows that trophozoite and schizont stages are very similar to one another but quite distinct from the early ring-stage parasites (Fig. 3, Supplementary Figs S2 and S3). Analysis of proteins differentially expressed between these distinct stages (that had a raw *P*-value of <0.05), including proteins enriched from ring- to trophozoite-stage, trophozoite- to schizont-stage, and finally schizont- to ring-stage, revealed sets of proteins specific to each of these stage transitions (Fig. 5). This analysis of the expression pattern of proteins across the stages was further confirmed and compared to Experiment 2 and another experiment (Experiment 3, Supplementary Data S7), which only contained the comparison of ring- to schizont-stage parasites (Supplementary Fig. S2; trophozoite-stage parasite data were not acquired). Enrichment analysis showed a distinct clustering of GO biological processes across each of the 3 developmental stages (Supplementary Data S8; Fig. 5). Comparison of the ring- to trophozoite-stage transition displayed a range of significantly overrepresented pathways (*P*  $\le $ 0.05), with the most significant clustered processes enriched in regulation of host cell entry and protein transport–related terms in rings, which collectively describes establishment of active infection within a new host RBC. In contrast, both trophozoite- to schizont- and schizont- to ring-stage transitions display a more specific clustering of enriched pathways; ribosomal biogenesis (up-regulated in trophozoites) is the most representative cluster in trophozoite- to schizont-stage, whereas processes relating to cell division (up-regulated in schizonts) are highly represented in schizont- to ring-stage parasites, consistent with the replicative end point of asexual development (Fig. 5).

**Figure 6:**
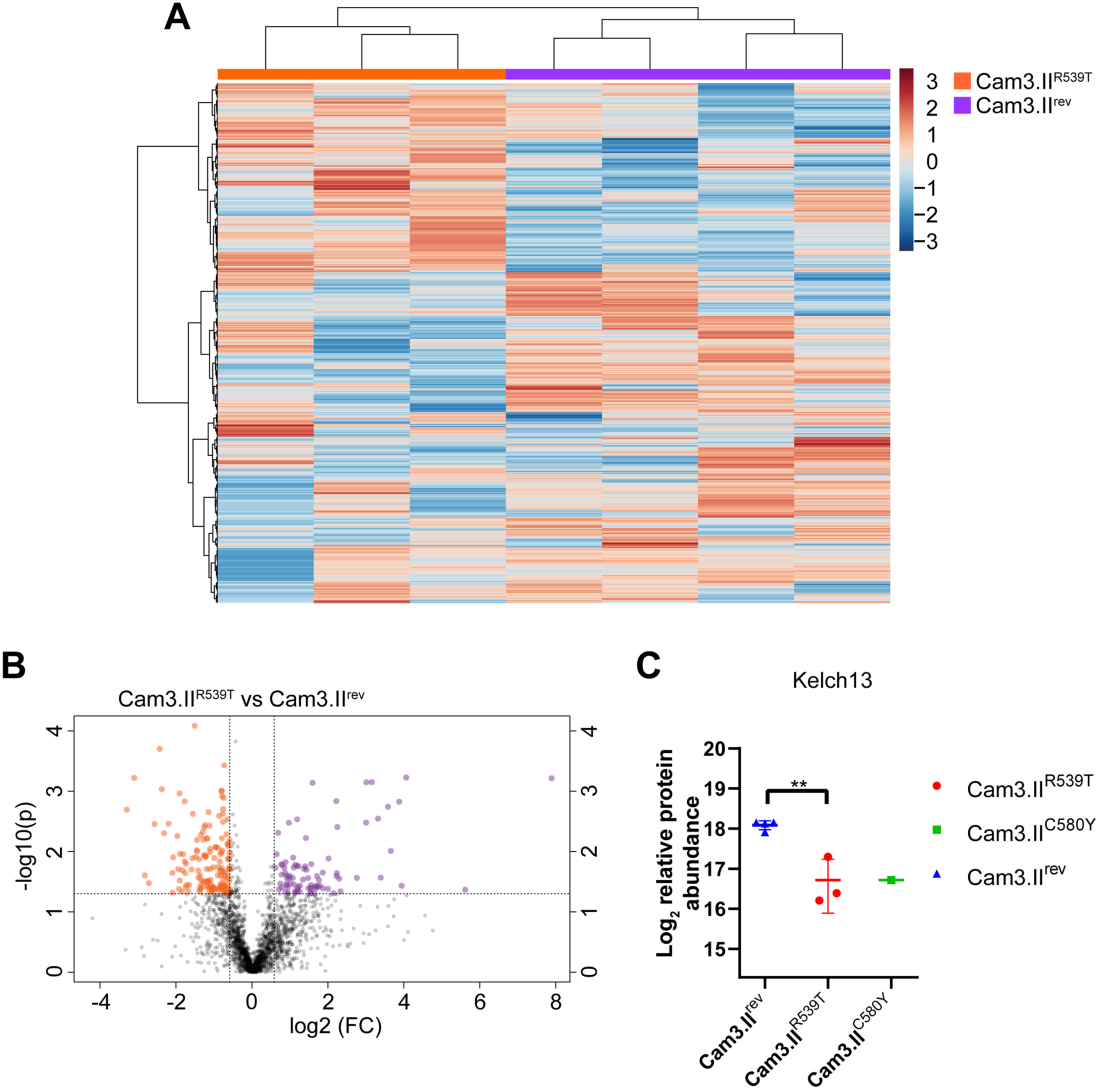
DIA-MS analysis of artemisinin-resistant (Cam3.II^R539T^) and artemisinin-sensitive (Cam3.II^rev^) parasites using the *P. falciparum* spectral library. (A) Hierarchical clustering of the *P. falciparum* proteins of Cam3.II^R539T^ (n = 3) and Cam3.II^rev^ (n = 4). Vertical clustering displays similarities between sample groups, while horizontal clusters reveal the relative abundances of identified *P. falciparum* proteins (2,317). The colour scale bar represents log_2_ (mean-centered and divided by the standard deviation of each variable) intensity values. (B) Volcano plot of differential protein abundance between Cam3.II^R539T^ and Cam3.II^rev^ parasites. Proteins above the significance threshold (*P* < 0.05) and fold change ≥ 1.5 are shown as orange (up-regulated in Cam3.II^R539T^) and purple (up-regulated in Cam3.II^rev^) dots. Data shown from ≥3 independent biological replicates. (C) log_2_ relative abundance (mean ± standard deviation) of PF3D7_1343700 (Kelch13) in Cam3.II^rev^, Cam3.II^R539T^, and Cam3.II^C580Y^. **P = 0.001.

**Figure 7:**
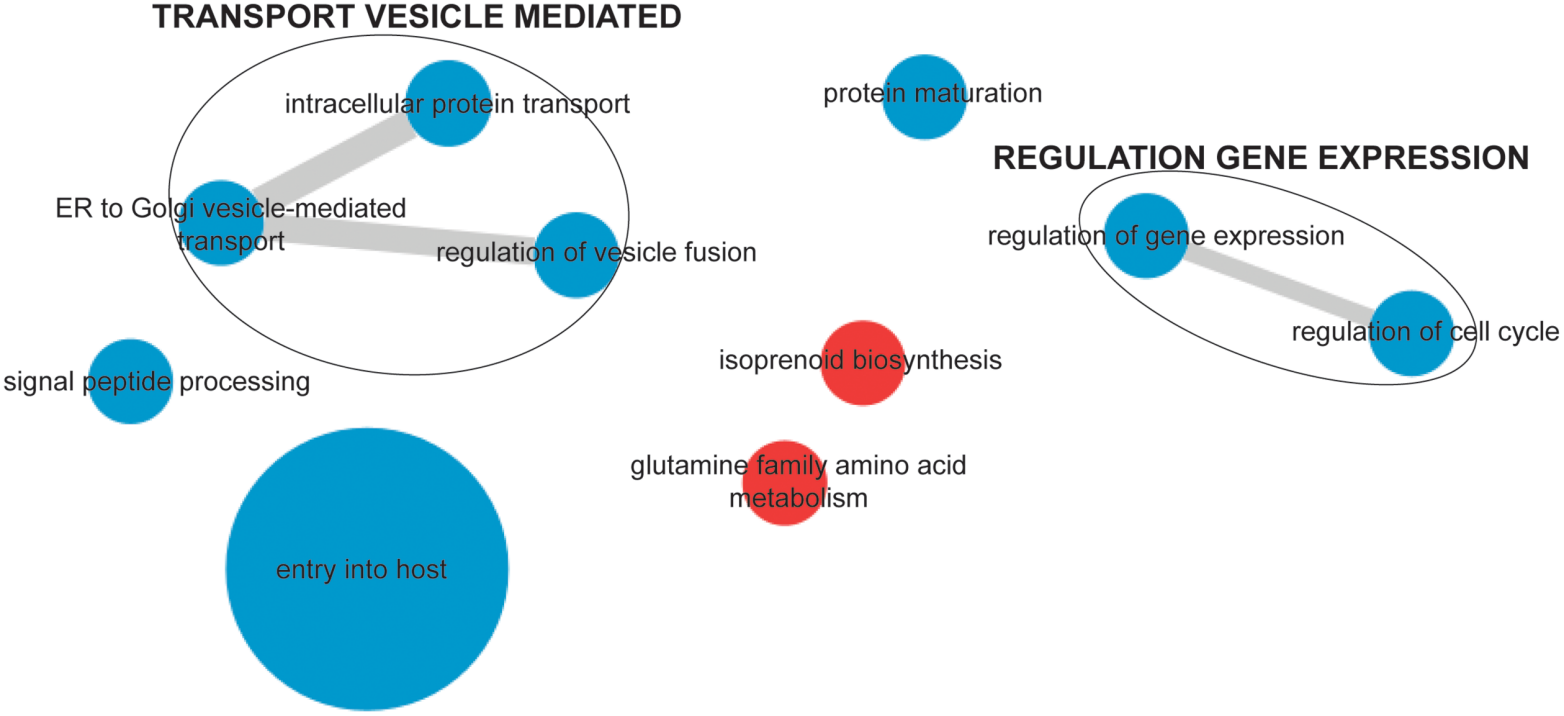
Comparative enrichment analysis of trophozoite-stage artemisinin-resistant (Cam3.II^R539T^) and artemisinin-sensitive (Cam3.II^rev^) parasites. Pathway enrichment networks were prepared from significantly overrepresented GO terms obtained by in-house statistical methods. Nodes are sized by increasing GO term uniqueness and coloured according to whether a process is up (red) or down (blue) in resistant versus sensitive parasites. ER: endoplasmic reticulum.

The common reference strain of *P. falciparum*, 3D7 wild-type strain, was used for the generation of this spectral library. To test whether this DIA-MS library can be used for other *P. falciparum* strains, we performed quantitative DIA-MS on 500 µg of protein lysate from the artemisinin-resistant Kelch-13-mutant Cambodian isolate, Cam3.II^R539T^, and the related artemisinin-sensitive Cam3.II^rev^ line. We identified a total of 2,317 *P. falciparum* proteins in all samples (Supplementary Data S9). Heat map and volcano plot analysis of all identified proteins demonstrated a greater number of proteins that were dysregulated between the 2 lines compared to previous proteomics analyses using a DDA-based approach (Fig. 6A and B) [24]. The abundance of 1 of the dysregulated proteins, Kelch13 (Pf3D7_1343700), was found to be 2-fold lower in artemisinin-resistant Cam3.II^R539T^ parasites compared to artemisinin-sensitive Cam3.II^rev^ (Fig. 6C), consistent with previous studies [24, 31, 32]. One sample from the related artemisinin-resistant line bearing a different mutation in *Kelch13*, Cam3.II^C580Y^, was also analysed to confirm proteins that were differentially dysregulated in artemisinin-resistant parasites compared to *Kelch13* wild-type (Cam3.II^rev^) parasites. Detailed analysis of GO term enrichment from dysregulated proteins identified entry into host cell and vesicular trafficking (*P* < 0.006) to be overrepresented in proteins significantly dysregulated (*P* > 0.05) (Fig. 7), suggesting a role for these processes in the mechanism of artemisinin resistance (Supplementary Data S10).

## Discussion

*P. falciparum* continues to cause the most severe form of malaria in humans. Despite years of study into the basic biochemical and molecular characteristics of these complex parasites, many questions remain unanswered. DDA-MS has been used extensively to reveal important features of the parasite's regulatory mechanisms. However, there are many issues with DDA-MS, including run-to-run reproducibility and identification of less abundant proteins. Therefore, a relatively high amount of starting material and extensive sample fractionation is required to generate high-quality proteomics data with reasonable depth of coverage. This is evident from our DDA-MS of each of the distinct stages, and also from previous publications, where 1–2 mg of starting material was used followed by extensive fractionation [24, 33]. In comparison, many of the DDA-MS problems can be circumvented using a data-independent acquisition (DIA-MS) methodology, which requires 2–4 times less biomass of starting material (500 µg) and avoids the need for fractionation (Fig. 1B).

Another benefit of generating a spectral library rather than using traditional DDA-MS is reproducibility and run-to-run consistency in regards to the identification of peptides and proteins. This is shown in our DDA-MS analysis of ring-, trophozoite-, and schizont-stage parasites for spectral library generation, where only 607 proteins were found to be common to all 3 stages (Fig. 2D). This is further supported by many published proteomics analyses of these 3 distinct stages, where 600–700 proteins were commonly identified [31, 34–36], and proteomics analysis of 1 distinct stage with different treatment conditions, where 600–1,000 proteins were identified [37, 38]. In contrast, with DIA-MS, the overlap of proteins detected across the 3 stages is frequently roughly of the order of 2,000 *P. falciparum* proteins—3 times more than in DDA-MS analysis (Fig. 4C, Supplementary Fig. 2A and C). Previous DDA-MS of artemisinin-resistant and -sensitive parasites had identified 2,824 proteins. However, owing to the nature of DDA-MS, only 520 proteins were included in the final analysis because the dataset contained many missing values [24]. Using DIA-MS methods in this study to analyse these same resistant and sensitive parasites led to the reproducible identification of 2,317 *P. falciparum* proteins across all samples, again demonstrating the benefits of DIA-MS using this spectral library compared to a typical DDA-MS. We were also able to demonstrate that DIA-MS experiments analysed by MS on different days were generally reproducible (Fig. 4A and B), and the median CV of protein abundances within each experiment was <10%, with the exception of ring-stage proteomics from Experiment 2 (Fig. 4C). Saponin lysis of mature iRBCs (trophozoites and schizonts) is more reproducible compared to ring-stage parasites, with a number of metabolomics and proteomic studies of this early stage demonstrating the heavy influence of host metabolites and proteins, subsequently contributing to the variability between samples [24, 39]. Despite this, we were still able to demonstrate that DIA-MS using the spectral library very clearly outperforms DDA-MS when it comes to reproducibility and run-to-run identification.

In quantitative proteomic studies, high quantification reproducibility is of utmost importance. Therefore, for the majority of DDA-based proteomics studies of *P. falciparum* asexual stages, labelling of peptides is usually necessary [24, 31, 37]. Labelling approaches generally limit the number of samples that can be included in a single study and are expensive and time consuming, while DIA-MS allows for label-free protein quantification across the entire proteome with quantification performance comparable to labelling methods [40]. This was evident from the DIA-MS of the resistant and sensitive parasites, where we identified Kelch13 to be decreased in abundance in artemisinin-resistant parasites, with comparable fold changes to those previously shown using peptide labelling [24] (Fig. 6C). Furthermore, our previous quantitative analysis of artemisinin-resistant and -sensitive parasites only identified Kelch13 to be dysregulated [24]. However, the DIA-MS demonstrated a larger number of proteins to be dysregulated between the 2 lines (Fig. 6). Enrichment analysis of significantly dysregulated proteins revealed 2 important processes—entry into host and vesicular-mediated transport (Fig. 7). Protein transport (down-regulated in artemisinin-resistant parasites) is of particular interest because Kelch13 has been shown to interact with a number of proteins involved in vesicular trafficking; more specifically Kelch13 was shown to be involved in endocytosis of host haemoglobin [32, 41]. Mutations in *Kelch13* alter haemoglobin uptake within the parasite, and the mechanism by which the parasite internalizes and transforms haemoglobin into haemozoin is central to artemisinin activation and efficacy [32, 41]. Therefore, a lack of haemoglobin uptake would result in increased artemisinin tolerance. Enrichment of the process entry into host cell (down-regulated in artemisinin-resistant parasites) is surprising and could be a secondary stress response of the resistant parasites by dysregulating the expression of invasion proteins. Previous studies have demonstrated drug-treated trophozoite-stage parasites to have dysregulated proteins involved in invasion [33, 37]. Another possibility for the enrichment of invasion proteins could be related to the role of Kelch13 in vesicular trafficking. Invasion proteins are sorted into secretory organelles, which are endosomal-like structures, and it is possible that mutation of Kelch13 protein affects either biogenesis of secretory organelles or sorting of invasion proteins into these organelles [42, 43].

Access to a detailed spectral library is critical for understanding the mechanisms by which *P. falciparum* parasites regulate their development, and identifying proteins important for each specific stage is a key element towards a rational design of agents for prophylaxis and treatment of malaria. Our study demonstrated that by combining this spectral library with the DIA-MS approach, we were able to add critical information about proteins expressed in the 3 distinct asexual blood stages of the *P. falciparum* parasite. Previously, quantitative analysis of these 3 distinct stages using DDA analysis demonstrated that there is a large proportion of proteins (54% of identified proteins) exhibiting variable expression across these stages [34]. However, this DIA-based analysis demonstrated that trophozoite- to schizont-stage parasite proteins are similar in expression, while ring- to trophozoite- or schizont-stage are very different (Fig. 3, Supplementary Figs S2 and S3). Analysis of the differences between ring and trophozoite stages found enrichment for parasite proteins predominantly involved in host cell invasion and protein sorting (vesicular trafficking) (Fig. 5). This is expected because these proteins are necessary for parasite invasion and establishment of a niche within the host RBC that will allow for the uptake and utilization of nutrients from the extracellular environment [44, 45]. Trophozoite-stage parasites are known to be the most metabolically active stage of development [46–48], and the enrichment of ribosomal biogenesis aligns with an active synthesis of proteins to kick-start metabolism. As the parasite ages to a schizont, the enrichment of cell division proteins is in preparation for mitotic production of daughter cells [49] (Fig. 5). These daughter cells, upon rupture of the infected cell, re-invade naive RBCs to propagate infection.

**Figure 8:**
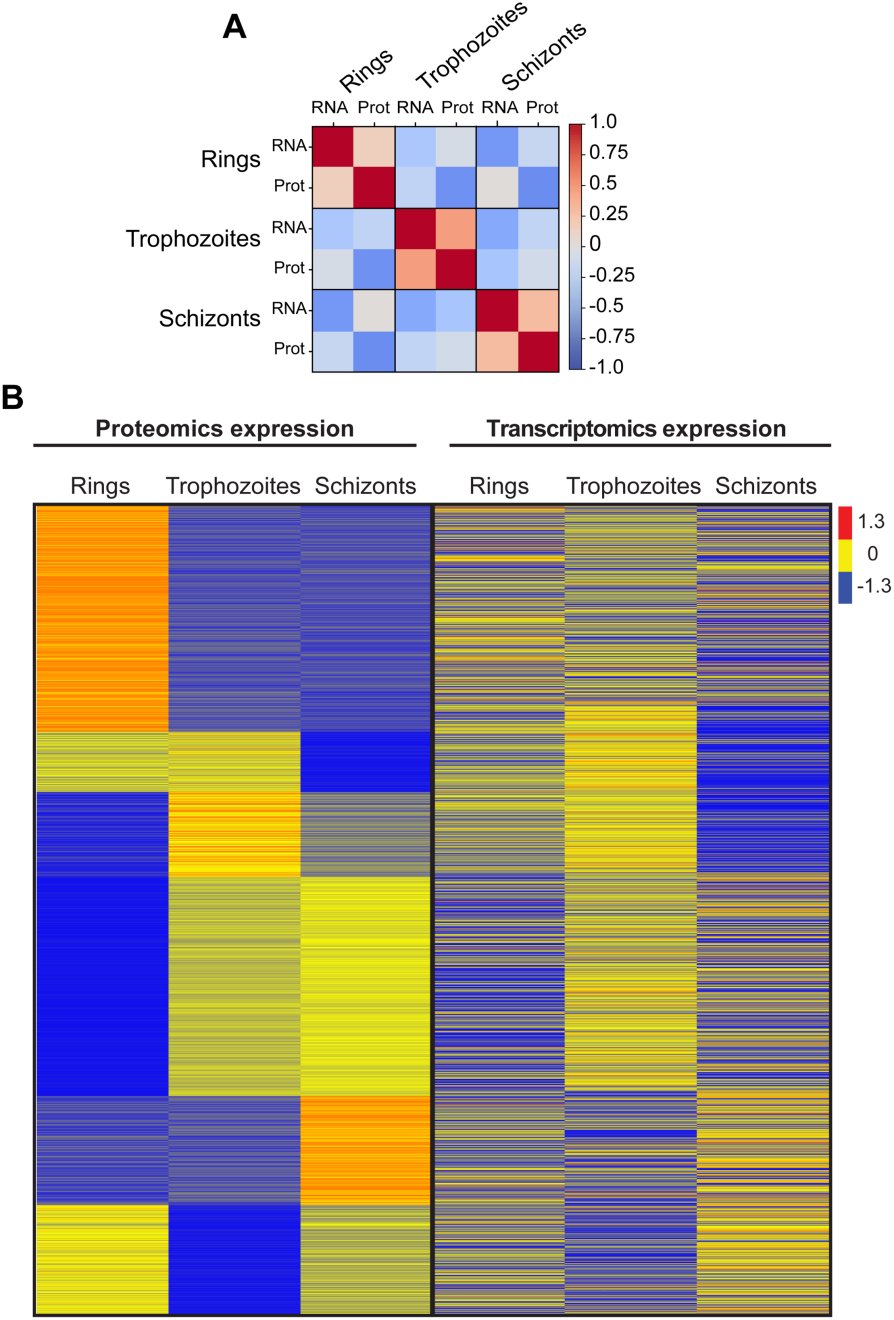
Heat map of the *P. falciparum* gene and protein expression patterns from ring- to trophozoite- to schizont-stage parasites. (A) Pearson correlation of mean protein intensities (1,990 common proteins identified in Experiments 1 and 2) in each specific stage (ring-, trophozoite-, and schizont-stage parasites) to RNA transcript abundance from Toenhake et al. [26]. (B) *Left:* Proteomics data (DIA) of 1,990 common proteins from ring- to trophozoite- to schizont-stage parasites in Experiments 1 and 2. *Right:* Transcriptomics data from Toenhake et al. [26]. The colour code is as follows: red indicates up-regulated proteins or transcripts; blue indicates down-regulated proteins or transcripts; yellow indicates unchanged proteins or transcripts.

It is well known that there is little correlation between mRNA and protein abundances in a number of systems, as a result of post-transcriptional and post-translational regulatory mechanisms. These mechanisms have also been shown to be important in regulation of gene expression in the asexual stages of *P. falciparum* parasites [50, 51]. We were interested in testing this observation using the quantitative analysis of the 3 distinct stages by comparing proteins in this library to published mRNA expression of the same stages (Supplementary Data S11) [26]. As per previous publications, our analysis showed that there is little to no correlation between protein and mRNA abundance (0.2 for rings, 0.5 for trophozoites, and 0.3 for schizonts) (Fig. 8A and B). Furthermore, translational repression is a common regulatory mechanism used by these parasites for an effective transition to other stages, which has been clearly demonstrated for gametocytes and sporozoites, where after fully maturing and upon successful transmission, they rapidly translate available mRNA [52]. Importantly, it is yet to be shown that translational repression is used in *P. falciparum* asexual stages and more work is warranted to resolve the role of these control mechanisms in this stage of the parasite's life cycle.

Spectral library searching, as opposed to sequence searching via *in silico* predicted fragmentation spectra, has greater sensitivity for peptide ions included in the library; however, relatively few DDA datasets are analysed in this way. A major concern is the incompleteness of the spectral library, and to address this issue, we analysed the completeness of the generated library by comparing it to the database of the *P. falciparum* genome from PlasmoDB. We found that this comprehensive library has 87% coverage of all previously reported *P. falciparum* proteins with protein-level evidence detected by MS; the other 13% missing from this library were only identified following specific peptide enrichment procedures, such as phosphoproteomics [25]. This library also added a further 692 proteins to the genes detectable at the protein level in asexual *P. falciparum* parasites (Fig. 2A). Analysis of mRNA expression for proteins not seen in our library suggested that they may be expressed in other stages of the *P. falciparum* life cycle, such as gametocytes (sexual stage of the parasite), oocyst, and sporozoites (mosquito stages of the parasite) [27, 28] (Fig. 2F). This demonstrates that this spectral library will be advantageous for the malaria community only for the analysis of the asexual stages, and further DDA proteomics data from other stages are required for the expansion of the library to cover additional stages of the life cycle.

In addition to the spectral library of the parasite, a library of the host RBC cytosol was also established. *P. falciparum* heavily modifies its host cell, and although much is known about modification of the host cell membrane, export of parasite proteins in the RBC cytosol and modification of RBC cytosolic proteins (such as phosphorylation [53]) remains to be further explored. This spectral library sets the tone for further investigations in host-parasite interactions and opens the door to host-directed therapy approaches.

An important consideration for the use of public libraries will be the portability of information between instrument types and laboratories. For example, can the DDA spectral library generated on a specific instrument platform be used to analyse DIA-MS data on a different platform from laboratories around the world? Although the best comparability is achieved when fragment ion spectra are generated on the same instrument platform [54], fragment ion spectra acquired from instruments using “beam type” collision-induced dissociation such as QTOFs and Q-Orbitrap [55] (used in this study) are sufficiently comparable for their effective use as prior knowledge in a peptide-centric analysis [56]. In regards to chromatographic information, owing to the addition of iRT peptides in the samples, the accuracy of such methods is generally tolerant to changes in gradient length and column dimensions; however, some misalignment will result from changes in other factors such as mobile/stationary phases or column temperature because they can affect peptide elution [57]. This suggests that our spectral library should provide a useful DIA reference library for most LC-MS/MS platforms with “beam type” fragmentation, but reproducibility for optimal DIA-MS analysis will be greatest when using a quadrupole-Orbitrap instrument with HCD fragmentation operating under the same LC-MS/MS conditions described here.

In conclusion, we have generated a comprehensive asexual stage spectral library and demonstrated that it can be successfully applied for consistent quantification of >2,000 *P. falciparum* proteins using a DIA-MS analysis pipeline. We showed that quantitative analysis is effective across different *P. falciparum* strains but is specific to the asexual blood stages of the parasite. Our freely accessible library will furnish the research malaria community with a resource to explore future proteome-phenotypic studies with the advantage of robustness, reproducibility, and streamlined procedures.

### Potential implications

Proteome spectral libraries are available for a number of model organisms, such as yeast and humans. Here, we provide the first MS-based proteome spectral library for *P. falciparum*, the most lethal malaria parasite that infects humans. In this study, we demonstrated how this library can facilitate DIA-MS proteomics analysis to understand basic biological processes of the parasite in distinct stages and can contribute to understanding drug resistance mechanisms in these parasites. Furthermore, the datasets generated in this study for the 3 distinct asexual blood stages will act as reference points for malaria researchers wishing to understand the expression profile of their protein of interest across the stages. From our successful application, we can be certain that spectral library–based quantitative DIA-MS will usher in a new wave of proteomics studies in the malaria research community across a wide range of applications, including in molecular classification, biomarker discovery, analysis of pathogenesis pathways, drug and vaccine discovery, and unravelling mechanisms of therapy response and resistance. The robustness and efficiency of DIA-MS will also overtake other semi-quantitative methods as the go-to method for measurement of protein abundances, allowing routine measurement of ∼2,000 proteins rather than single-protein measurements provided by antibody-based approaches.

## Methods

### *P. falciparum* spectral library generation using parasite pellets

Asexual *P. falciparum* (3D7 reference strain, Cam3.II^R539T^, Cam3.II^C580Y^, and Cam3.II^rev^) were cultured as per standard methods [58], with minor adjustments [59]. Briefly, cultures were maintained using O-positive human RBCs (Australian Red Cross Blood Service) at 2% or 4% haematocrit (HCT) in modified RPMI 1640 medium containing hypoxanthine and 0.5% (w/v) Albumax II (Gibco, Australia) at 37°C under defined atmospheric conditions (95% nitrogen, 4% carbon dioxide, 1% oxygen).

To achieve tightly synchronous cultures for library generation, *P. falciparum* cultures with a high proportion of ring-stage parasites (30 mL, 10–12% parasitaemia, 2% HCT) were synchronized by performing sorbitol lysis twice, 10 h apart. For schizont library generation, parasites were harvested 27 h after the second sorbitol lysis. At this time point, the cultures contained a high proportion of segmented schizonts and a small proportion of young ring-stage parasites (<10%). For ring library generation, parasites were harvested in the second cycle (16–18 h.p.i.), while trophozoites were harvested 28–30 h.p.i. Proteomic samples were prepared as previously described with minor modifications [24, 60]. For generating stage-specific samples, iRBCs were pelleted by centrifugation (650*g*, 5 min) and parasites were isolated from RBCs by resuspending in saponin lysis buffer (0.1% w/v in phosphate-buffered saline [PBS]) containing protease and phosphatase inhibitors ([PPI]; 1 × complete mini protease inhibitor cocktail [Roche, Australia], 20 mM sodium fluoride, and 0.1 mM sodium orthovanadate) and incubated for 10 min on ice. Isolated parasites were pelleted (4,000*g*, 7 min) and washed (15,850*g*, 3 min, supernatant discarded after each wash) a total of 3 times in 1 mL 1 × PBS with PPI.

### *P. falciparum* library generation using cytosolic uRBC and iRBC fractions

Synchronous cultures were obtained with a double sorbitol lysis (6 h interval). At ∼30 h.p.i., iRBCs were collected by magnet purification [61], quantified using a Neubauer hemocytometer (ThermoFisher Scientific, Australia), and 10^9^ iRBCs were aliquoted in microtubes. As a control, 10^9^ uRBCs from the same donor were also aliquoted into microfuge tubes. This was performed 10 times (using RBCs from different donors) and the protein samples were pooled together to achieve 10^10^ uRBCs and 10^10^ iRBCs.

The uRBCs and iRBCs were lysed for 10 min on ice in 600 μL saponin lysis buffer (as described above). After centrifugation (16,200*g*, 5 min, 4°C), the supernatant, containing the cytosolic fraction of the RBCs, was carefully collected and loaded onto 600 μL of TALON® Metal Affinity Resin slurry (Takara, U.S) and washed with an equal volume of 1× PBS with PPI (600*g*, 2 min) to remove haemoglobin. Samples were incubated with the resin for 10 min on a rotating wheel at 4°C, and the haemoglobin-free supernatant was collected (2 min at 600*g*; 2 min at 2,400*g*, transferring the supernatant to fresh pre-chilled microfuge tubes after each wash). Ice-cold TCA was added to samples (1:20 of final volume) and incubated for 10 min on ice to facilitate protein precipitation. The pellet was washed in 1 mL acetone (16,200*g*, 3 min) and the acetone removed and evaporated before samples were stored at −80°C until required.

### Sample collection for quantitative DIA-MS using *P. falciparum* spectral library

To achieve tightly synchronous cultures for quantitative DIA-MS analysis, late schizont-stage parasites were enriched by magnet purification [61]. The highly enriched parasite fraction was then added to fresh uRBCs (2% HCT) and left to invade for 3 h before sorbitol synchronization. For 3D7 parasites, the parasitaemia was adjusted to 16% and 45 mL was used to prepare ring-stage samples (6 h.p.i.). For trophozoite and schizont stages, 15 mL of the same sample were adjusted to 8% parasitaemia and samples prepared at 22–28 and 38–42 h.p.i., respectively. For artemisinin-resistant (Cam3.II^R539T^, Cam3.II^C580Y^) and artemisinin-sensitive (Cam3.II^rev^) parasites, the parasitaemia was adjusted to 8%, and 15 mL of trophozoite-stage parasites at 22–26 h.p.i. were collected.

### Sample preparation for *P. falciparum* spectral library

Saponin pellets were solubilized with SDC lysis buffer (100 mM 4-(2-hydroxyethyl)-1-piperazineethanesulfonic acid [HEPES], 1% sodium deoxycholate [SDC], pH 8.1) supplemented with PPI and probe sonicated with 3 pulses at 30 sec each. Following sonication, samples were boiled at 95°C for 5 mins and allowed to return to room temperature (RT) before reducing and alkylating with tris(2-carboxyethyl) phosphine (TCEP) (10 mM final) and iodoacetamide (40 mM final) at 95°C for 5 mins. After returning to RT, proteins were precipitated using ice-cold TCA and pellets were resuspended in SDC lysis buffer without PPI and sonicated to aid protein solubilization. Protein concentration was measured using the Pierce bicinchoninic acid (BCA) protein assay (ThermoFisher Scientific, Australia) and samples adjusted to 3–5 mg of protein per sample. Trypsin (1:50; Promega, Australia) was added and samples were incubated 16 h at 37°C with constant agitation at 1,500 rpm in a Multi-Therm™ (Benchmark Scientific, U.S). On the following day, trypsin activity was quenched using 5% (v/v) formic acid (FA), before adding 100% (v/v) ethyl acetate to remove detergent. The samples were centrifuged at 4000*g* for 5 mins, and the top layer of supernatant was removed. Samples were dried using CentriVap Benchtop Centrifugal Vacuum Concentrator (Labconco, U.S) at 100 mbar and 37°C for 10 mins to remove excess ethyl acetate. The samples were then diluted 5-fold and loaded onto an SCX Bond Elut Plexa (Agilent, Australia) and eluted into 12 fractions as described previously [24]. Peptides were then eluted from SCX cartridges using 500 μL of elution buffers at a rate of 1 drop/sec. Elution buffers consisted of increasing concentrations of ammonium acetate (Sigma-Aldrich, Australia) (75, 100, 125, 150, 175, 225, 250, 275, 300, 325, and 350 mM) with 20% (v/v) acetonitrile (ACN) and 0.5% (v/v) FA. The final elution buffer consisted of 80% ACN and 5% ammonium hydroxide (Sigma-Aldrich, Australia) to remove any remaining bound peptides. Eluates (fractions) were semi-dried to remove most of the ACN and then subjected to desalting using in-house–generated StageTips as described previously [62]. The desalted fractions were dried to completion and reconstituted in 20 μL of 2% ACN and 0.1% FA, sonicated for 15 mins, and subjected to automatic vortexing for a further 15 mins to allow complete resuspension of peptides. To facilitate retention time alignments among samples, a retention time kit (iRT kit, Biognosys, GmbH, Switzerland) was spiked at a concentration of 1:20 (v/v) for all fractions [63]. Samples were stored at −80°C, and the particle-free supernatant was transferred to LC-MS vials immediately prior to LC-MS/MS analysis.

### Sample preparation for quantitative DIA-MS

Sample preparation for DIA-MS was mostly the same as sample preparation for spectral library generation with minor modifications. Significantly less protein material was digested overnight with trypsin, 500 µg compared to 3–5 mg. On the following day, the peptide samples were not subjected to SCX fractionation but rather directly desalted using in-house–generated StageTips as described previously [62]. The desalted peptide samples were then dried to completion and reconstituted in 20 μL of 2% ACN and 0.1% FA with iRT peptides [63]. Samples were stored at −80°C, and the particle-free supernatant was transferred to LC-MS vials immediately prior to LC-MS/MS analysis.

### Mass spectrometric instrumentation and data acquisition

For DDA acquisition, NanoLC-MS/MS was carried out on a Q Exactive HF Orbitrap LC-MS/MS system as described previously [24], with minor modifications. Samples were loaded at a flow rate of 15 μL/min onto a reversed-phase trap column (100 μm × 2 cm), Acclaim PepMap media (Dionex, ThermoFisher Scientific, Australia), and maintained at a temperature of 40°C. Peptides were eluted from the trap column at a flow rate of 0.25 μL/min through a reversed-phase capillary column (75 μm × 50 cm) (LC Packings, Dionex, ThermoFisher Scientific, Australia). For acquisition by HPLC, a 158-min gradient was set using an incremental gradient that reached 30% ACN after 123 min, 34% ACN after 126 min, 79% ACN after 131 min, and 2% ACN after 138 min for a further 20 min. The mass spectrometer was operated in a data-dependent mode with each cycle comprising 1 full scan performed at 70,000 resolution (AGC target of 3e^6^, mass range of 375–1,575 *m/z*, and maximum injection time of 54 ms). The 20 most intense precursors with charge states 2–6 were selected for fragmentation at 17,500 resolution (AGC target of 5e^5^, normalized collision energy of 27.0, activation time of 15 ms, isolation window of 1.8 *m/z*, maximum injection time of 118 ms, and dynamic exclusion enabled). For DIA, a 25-fixed-window set-up of 24 *m/z* effective precursor isolation over the *m/z* range of 376–967 Da was applied. Full scan was the same as DDA mode with fragmentation resolution at 17,500 (AGC target of 2e^5^, maximum injection time at auto, normalized collision energy of 27.0).

### Shotgun data searching and spectral library generation

DDA files were searched against *P. falciparum* (UP000001450, release version 2016_04) and *Homo sapiens* (UP000005640, release version 2017_05) UniProt FASTA databases and the Biognosys iRT peptides database. The number of entries in the database actually searched were 3,970,852 with trypsin as enzyme specificity and 2 missed cleavages were permitted. Carbamidomethylation of cysteines was set as a fixed modification. Oxidation of methionine and protein N-terminal acetylation were set as variable modifications. Parent mass error tolerance and fragment mass tolerance were set to 20 ppm. For both peptide and protein identification, a false discovery rate of 1% was used. MaxQuant search results were imported as spectral libraries into Spectronaut using default settings and iRT values were computed using the linear iRT regression function embedded in Spectronaut. A consensus library was generated for *P. falciparum* iRBCs and saved for downstream targeted analysis. The consensus library contained 44,449 peptides corresponding to 4,730 proteins.

### Spectronaut targeted data extraction

Raw files were processed using Spectronaut^TM^ (version 13.0) against the in-house–generated *P. falciparum* spectral library. For processing, raw files were loaded in Spectronaut; the ideal mass tolerances for data extraction and scoring were calculated on its extensive mass calibration with a correction factor of 1. Both at precursor and fragment level, the highest data-point within the selected *m/z* tolerance was chosen. Identification of peptides against the library was based on default Spectronaut settings (Manual for Spectronaut 13.0, [64] ). Briefly, precursor Qvalue Cut-off and Protein Qvalue Cut-off were as per default at 1% and therefore only those that passed this cut-off were considered as identified and used for subsequent processing. Retention time prediction type was set to dynamic iRT. Interference correction was performed at the MS2 level. For quantification, interference correction was activated and cross-run normalization was performed using the total peak area at a significance threshold of 0.01. Fold-changes for the ring stage versus trophozoite stage and schizont stage were calculated in Microsoft Excel and *P*-values were calculated using a standard *t*-test. Volcano plots and hierarchical clustering were performed in Metaboanalyst [65]. Hierarchical clustering analysis was carried out on 2 sets, namely, all identified P*. falciparum* proteins and 400 differentially regulated proteins.

### Comparative analysis of protein to mRNA expression

The public transcriptomics data for the same developmental stages of *P. falciparum* from Toenhake et al. [26] were obtained from the Gene Expression Omnibus repository (Accession No.: GSE104075). Count data were generated by mapping to the reference genome of *P. falciparum* (P3D7-release-39) using the RNAsik pipeline implemented in the Laxy platform [66]. The transcriptomics data were then CPM normalized in Degust Software. The comparative analysis was carried out in R for common gene/protein expressions found in both transcriptomics and proteomics data. First, the mean expression of each protein was calculated for each developmental stage, which was then used to calculate Pearson correlation coefficient between transcript and protein abundance.

### GO Enrichment analysis

Protein abundance values were log-transformed and subjected to pairwise *t*-tests to assess differences in abundance between ring, trophozoite, and schizont stages, or between Cam3.II^R539T^ and Cam3.II^rev^ parasites. The resulting *P*-values were used as protein scores in a GO enrichment analysis using topGO, using the classic algorithm and K-S statistic to assess GO-term enrichment.

Overrepresented GO terms extracted were imported into the Reduce and Visualize Gene Ontology (REVIGO) web server [67] using default parameters with *P. falciparum* as the chosen database for term size. The resulting output file containing summarized GO terms (redundant terms removed) was visualized in Cytoscape (v3.8.0) [68]. Nodes were sized according to GO term uniqueness (i.e., fewer redundant terms merged with more general, higher-order terms). Nodes were coloured by fold-change up (red) or down (blue) when compared between life cycle stages or resistant versus sensitive parasites.

## Data Availability

All the raw data (DDA) and search result files (MaxQuant Excel output) used to generate the *P. falciparum* spectral library have been deposited in the ProteomeXchange Consortium through the PRIDE partner repository [69] with identifier PXD027241. The raw data (DIA-MS) files generated in this study for quantitative analysis and their Spectronaut protein intensity have been deposited in the ProteomeXchange Consortium through the PRIDE partner repository [69] with identifier PXD027301. Other data further supporting this work are openly available in the *GigaScience* repository, GigaDB [70].

## Additional Files

**Supplementary Figure S1**. Venn diagrams depicting overlaps of identified proteins by data-dependent acquisition .

**Supplementary Figure S2**. Venn diagram comparison and hierarchical cluster analysis of differentially expressed proteins.

**Supplementary Figure S3**. Volcano plot of differential protein abundance from 2,064 identified *P. falciparum* proteins from Experiment 1.

**Supplementary Data S1**. Intensities of proteins identified in the spectral library. Uniprot IDs for *H. sapiens* proteins and PlasmoDB IDs for *P. falciparum* proteins with their corresponding gene names are provided. For each gene, the relative protein intensities measured using a DDA approach in different stages of the parasite red blood cell stage (ring, trophozoite, and schizont stage) and supernatant of infected cell are also provided.

**Supplementary Data S2**. Comparison of *P. falciparum* proteins in this spectral library with PlasmoDB. Annotated as “common” are proteins that are identified in this spectral library and have mass spectrometry (MS) evidence in the red blood cell (RBC) stage of infection from PlasmoDB. Annotated as “not seen” are proteins not identified in this spectral library and that also have no MS evidence of being present in the RBC stage of infection. Annotated as “PlasmoDB-unique” are proteins that have MS evidence in the RBC stage of infection but were not detected in this spectral library. Annotated as “spectral-library-unique” are proteins that have MS evidence in the RBC stage of infection from PlasmoDB but were identified in this spectral library.

**Supplementary Data S3**. Number of proteolytic peptides per protein of *H. sapiens* and *P. falciparum* identified in this spectral library.

**Supplementary Data S4**. Protein intensities for DIA Experiment 1: Ring- vs trophozoite- vs schizont-stage parasites with 3 biological replicates per condition.

**Supplementary Data S5**. Protein intensities for DIA Experiment 2: Ring- vs trophozoite- vs schizont-stage parasites, with 2–3 biological replicates per condition.

**Supplementary Data S6**. Comparison of commonly identified *P. falciparum* protein expression of Experiments 1 and 2.

**Supplementary Data S7**. Protein intensities for DIA Experiment 3: Ring- vs schizont-stage parasites with a minimum of 3 biological replicates per condition.

**Supplementary Data S8**. GO terms (with protein IDs in GO term) of differentially expressed proteins. Annotated as “rings to trophozoites enrichment” are GO terms enriched in ring-stage parasites compared to trophozoites. Annotated as “schizonts to rings enrichment” are GO terms enriched in schizont-stage parasites compared to rings. Annotated as “trophozoites to schizonts enrichment” are GO terms enriched in trophozoite-stage parasites compared to schizonts.

**Supplementary Data S9**. Protein intensities for DIA experiments—artemisinin resistant (Cam3.II^R539T^) vs sensitive (Cam3.II^rev^).

**Supplementary Data S10**. GO terms (with protein IDs in GO term) of differentially expressed proteins in artemisinin-resistant line (Cam3.II^R539T^) compared to artemisinin-sensitive (Cam3.II^rev^).

**Supplementary Data S11**. Comparison of protein expression (common proteins identified from Experiments 1 and 2) from the 3 distinct stages of the red blood cell infection to published RNA levels.

giac008_GIGA-D-21-00227_Original_Submission

giac008_GIGA-D-21-00227_Revision_1

giac008_Response_to_Reviewer_Comments_Revision_1

giac008_Reviewer_1_Report_Original_Submission Samantha Emery -- 9/17/2021 Reviewed

giac008_Reviewer_2_Report_Original_Submission Guenther Kahlert -- 9/21/2021 Reviewed

giac008_Reviewer_3_Report_Original_Submission Radoslaw Sobota -- 9/23/2021 Reviewed

giac008_Supplemental_Files

## Abbreviations

ACN: acetonitrile; ART: artemisinin; CV: coefficient of variation; DDA: data-dependent acquisition; DIA: data-independent acquisition; FA: formic acid; GO: Gene Ontology; h.p.i.: hours post invasion; iRBC: infected red blood cell; iRT: indexed retention time; K-S: Kolmogorov-Smirnov; LC-MS/MS: liquid chromatography tandem mass spectrometry; mRNA: messenger RNA; *m/z*: mass-to-charge ratio; Kelch13: PfKelch13; MS: mass spectrometry; PBS: phosphate-buffered saline; PPI: protease and phosphatase inhibitors; RBC: red blood cell; TCA: trichloroacetic acid; uRBC: uninfected red blood cell.

## Competing Interests

The authors declare that they have no competing interests.

## Funding

Funding support was provided by the Australian National Health and Medical Research Council (NHMRC) project grants Nos. APP1128003 and APP1160705 and fellowship to D.J.C. (No. APP1148700).

## Authors’ Contributions

G.S., A.D., A.E.S., and C.B. performed the experiments. G.S. processed the data and analysed the results. G.S., C.A.M., A.D.S., and M.B.B. conducted further analysis of the results. G.S., A.D., A.E.S., C.B., C.A.M., A.D.S., M.B.B., and R.B.S. drafted the manuscript. T.G.C. and D.J.C. supervised the study. All authors revised and approved the final version of the manuscript. The authors have no conflicts of interest to declare that are relevant to the content of this article.
