# Supplementary material for: A new mass spectral library for high-coverage and reproducible analysis of the Plasmodium falciparum–infected red blood cell proteome
Source: Gigascience. 2022 Mar 7;11:giac008. doi: 10.1093/gigascience/giac008 (PMC8900498; doi:10.1093/gigascience/giac008)
Supplement: giac008_GIGA-D-21-00227_Revision_1 [file giac008_giga-d-21-00227_revision_1.pdf]

GigaScience

A new mass spectral library for high-coverage and reproducible analysis of the Plasmodium falciparum-infected red blood cell proteome

--Manuscript Draft--

Manuscript Number:	GIGA-D-21-00227R1
Full Title:	A new mass spectral library for high-coverage and reproducible analysis of the Plasmodium falciparum-infected red blood cell proteome
Article Type:	Research
Funding Information:	national health and medical research council (APP1128003) — A/Prof Darren J Creek<br>national health and medical research council (APP1160705) — A/Prof Darren J Creek<br>national health and medical research council (APP1148700) — A/Prof Darren J Creek
Abstract:	Background: Plasmodium falciparum causes the majority of malaria mortality worldwide, and the disease occurs during the asexual red blood cell (RBC) stage of infection. In the absence of an effective and available vaccine, and with increasing drug resistance, asexual RBC stage parasites are an important research focus. In recent years, mass spectrometry-based proteomics using Data Dependent Acquisition (DDA) has been extensively used to understand the biochemical processes within the parasite. However, DDA is problematic for the detection of low abundance proteins, proteome coverage, and has poor run-to-run reproducibility.<br>Results: Here, we present a comprehensive P. falciparum -infected RBC (iRBC) spectral library to measure the abundance of 44,449 peptides from 3,113 P. falciparum and 1,617 RBC proteins using a Data Independent Acquisition (DIA) mass spectrometric approach. The spectral library includes proteins expressed in the three morphologically distinct RBC stages (ring, trophozoite, schizont), the RBC compartment of trophozoite-iRBCs, and the cytosolic fraction from uninfected RBCs (uRBC). This spectral library contains 87% of all P. falciparum proteins that have previously been reported with protein-level evidence in blood stages, as well as 692 previously unidentified proteins. The P. falciparum spectral library was successfully applied to generate semi-quantitative proteomics datasets that characterise the three distinct asexual parasite stages in RBCs, and compare artemisinin resistant (Cam3.II R539T) and sensitive (Cam3.II rev) parasites.<br>Conclusion : A reproducible, high-coverage proteomics spectral library and analysis method has been generated for investigating sets of proteins expressed in the iRBC stage of P. falciparum malaria. This will provide a foundation for an improved understanding of parasite biology, pathogenesis, drug mechanisms and vaccine candidate discovery for malaria.
Corresponding Author:	Darren J Creek<br><br>AUSTRALIA
Corresponding Author Secondary Information:	
Corresponding Author's Institution:	
Corresponding Author's Secondary Institution:	
First Author:	Ghizal Siddiqui
First Author Secondary Information:	
Order of Authors:	Ghizal Siddiqui
	Amanda De Paoli

Powered by Editorial Manager® and ProduXion Manager® from Aries Systems Corporation

	Christopher A MacRaild
	Anna E Sexton
	Coralie Boulet
	Anup D Shah
	Mitchell B Batty
	Ralf B Schittenhelm
	Teresa G Carvalho
	Darren J Creek
**Order of Authors Secondary Information:**	
**Response to Reviewers:**	We thank the editor and reviewers for a thorough and helpful review of this manuscript. The suggested edits have been added to the manuscript and a point-by-point response to each reviewer comment is below:<br><br>Reviewer #1.<br>1.The reviewer has asked for how this DDA library performs compared to other DIA searching strategies, including using options for (1) spectral library prediction and (2) library-free searches? Can they demonstrate benefits, either in terms of coverage or detecting lower abundance proteins?<br><br>Response: We thank the reviewer for the comment and have performed a DirectDIA search (library-free search) using our dataset (Experiment 1 comparing the distinct stages of the red blood cell stage infection) and found that spectral-library searches resulted in significantly greater number of proteotypic peptides compared to DirectDIA. Furthermore, this finding is consistent with previous reports that compared these types of approaches, and references have been included. This additional analysis is added to the results section, lines 204-211.<br><br>2.Can the Venn diagrams in Fig1C, Fig1D, Fig2C be proportional as formatted in the Venn in Fig1A?<br><br>Response: The Figures have been modified as per suggestion.<br><br>3.I was not able to access the data in PDX027241 as no dataset was available when I used the reviewer login - can the authors double check and verify this?<br><br>Response: The data have now been made publicly available, so the reviewers should now be able to access it.<br><br>4.After downloading supplementary data tables, I noted that there were often several .csv files for the same supplementary data table number. Are these intended to be separate sheets in a single excel? The descriptions of the SD are very short, and extending the supplementary data table descriptions on pg21.<br>Response: We thank the reviewer for the comment, and we have modified the csv files to combine a number of supplementary dataset and have added detailed description for each of the supplementary data sheets in pg 21, lines 572-606. The supplementary datasheet numbers have also been modified and this has been updated within the manuscript.<br><br>5.In SD1 can the protein IDs be tidied up to separate protein IDS and gene names and a column added with cleaned UniProt/PlasmoDB ascensions?<br><br>Response: This has been modified as per suggestion.<br><br>6.SD2 "Supplementary_data_sheet_2-non-seen" doesn't appear to have the same column formats as all the other tables for SD2?<br><br>Response: This has been modified as per suggestion.<br><br>7.Can the SD4 sheets be combined into a single table for ease of reference?

Powered by Editorial Manager® and ProduXion Manager® from Aries Systems Corporation

	Response: We thank the reviewer for the comment, however due to journal requirements, we are unable to combine into a single sheet, as they have to be individual csv files for access of data to those interested in reproducing our findings.<br><br>8.Can the protein IDs in each GO cluster be added to the SD6 tables for each GO term?<br><br>Response: This has been modified as per suggestion.<br><br><br>Reviewer #2.<br>1.The authors did not consider in the introduction and the discussion is that there are different types of mass spectrometers and their fragmentation pattern has to be noted. DIA methods were first introduced for TOF instruments and using Orbitrap mass spectrometers provides different challenges in fragmentation and dwell times. Please add more in detail information about this topic to the manuscript.<br><br>Response: We thank the reviewer for the comment and we have added a paragraph in the discussion addressing their points in lines 374-386.<br><br><br>Reviewer #3.<br>1.Please provide details of instrumentation and DIA analysis (fragment ions resolution etc.)<br><br>Response: We thank the reviewer for the comment and we have modified the methods section as requested, please see lines 494-510.
**Additional Information:**	
**Question**	**Response**
Are you submitting this manuscript to a special series or article collection?	No
**Experimental design and statistics**<br><br>Full details of the experimental design and statistical methods used should be given in the Methods section, as detailed in our Minimum Standards Reporting Checklist. Information essential to interpreting the data presented should be made available in the figure legends.<br><br>Have you included all the information requested in your manuscript?	Yes
**Resources**<br><br>A description of all resources used, including antibodies, cell lines, animals and software tools, with enough information to allow them to be uniquely identified, should be included in the	Yes

Methods section. Authors are strongly encouraged to cite Research Resource Identifiers (RRIDs) for antibodies, model organisms and tools, where possible.<br><br>Have you included the information requested as detailed in our Minimum Standards Reporting Checklist?	
**Availability of data and materials**<br><br>All datasets and code on which the conclusions of the paper rely must be either included in your submission or deposited in publicly available repositories (where available and ethically appropriate), referencing such data using a unique identifier in the references and in the “Availability of Data and Materials” section of your manuscript.<br><br>Have you have met the above requirement as detailed in our Minimum Standards Reporting Checklist?	Yes

Manuscript with DOI

Click here to access/download;Manuscript;Submission-GigaScience-cleaned DOI.docx

A new mass spectral library for high-coverage and reproducible analysis of the *Plasmodium falciparum*-infected red blood cell proteome

Authors: Ghizal Siddiqui[1*], Amanda De Paoli[1], Christopher A. MacRaild[1], Anna E. Sexton[1], Coralie Boulet[2], Anup D. Shah[3,4], Mitchell B. Batty[1], Ralf B. Schittenhelm[3], Teresa G. Carvalho[2], and Darren J. Creek[1*]

[1]Monash Institute of Pharmaceutical Sciences, Monash University, Melbourne, Australia; [2]Department of Physiology, Anatomy and Microbiology, La Trobe University, Bundoora, Victoria 3086, Australia, [3]Monash Proteomics & Metabolomics Facility, Department of Biochemistry and Molecular Biology, Biomedicine Discovery Institute, Monash University, Clayton, Victoria 3800, Australia, [4]Monash Bioinformatics Platform, Biomedicine Discovery Institute, Monash University, Clayton, Victoria 3800, Australia.

* Corresponding authors: Darren J. Creek, Drug Delivery, Disposition and Dynamics, Monash Institute of Pharmaceutical Sciences, Monash University, Parkville Campus, Parkville, Victoria, Australia. Tel: +61 (0) 3 9903 9249; Fax: +61 (0) 3 9903 9583; e-mail, Darren.creek@monash.edu

Ghizal Siddiqui, Drug Delivery, Disposition and Dynamics, Monash Institute of Pharmaceutical Sciences, Monash University, Parkville Campus, Parkville, Victoria, Australia. Tel: +61 (0) 3 9903 9282; e-mail, Ghizal.siddiqui@monash.edu

Abstract

Background: *Plasmodium falciparum* causes the majority of malaria mortality worldwide, and the disease occurs during the asexual red blood cell (RBC) stage of infection. In the absence of an effective and available vaccine, and with increasing drug resistance, asexual RBC stage parasites are an important research focus. In recent years, mass spectrometry-based proteomics using Data Dependent Acquisition (DDA) has been extensively used to understand the biochemical processes within the parasite. However, DDA is problematic for the detection of low abundance proteins, proteome coverage, and has poor run-to-run reproducibility.

Results: Here, we present a comprehensive *P. falciparum*-infected RBC (iRBC) spectral library to measure the abundance of 44,449 peptides from 3,113 *P. falciparum* and 1,617 RBC proteins using a Data Independent Acquisition (DIA) mass spectrometric approach. The spectral library includes proteins expressed in the three morphologically distinct RBC stages (ring, trophozoite, schizont), the RBC compartment of trophozoite-iRBCs, and the cytosolic fraction from uninfected RBCs (uRBC). This spectral library contains 87% of all *P. falciparum* proteins that have previously been reported with protein-level evidence in blood stages, as well as 692 previously unidentified proteins. The *P. falciparum* spectral library was successfully applied to generate semi-quantitative proteomics datasets

that characterise the three distinct asexual parasite stages in RBCs, and compared artemisinin resistant (Cam3.II^{R539T}) and sensitive (Cam3.II^{rev}) parasites.

Conclusion: A reproducible, high-coverage proteomics spectral library and analysis method has been generated for investigating sets of proteins expressed in the iRBC stage of *P. falciparum* malaria. This will provide a foundation for an improved understanding of parasite biology, pathogenesis, drug mechanisms and vaccine candidate discovery for malaria.

Keywords: *Plasmodium falciparum*, Malaria, Proteomics, Data dependent acquisition, Data independent acquisition, Red blood cells, LC-MS/MS

Background

Malaria, a mosquito-borne disease caused by *Plasmodium* parasites, is endemic in countries of Southeast Asia, Latin America and the sub-Saharan regions of Africa. *Plasmodium falciparum* is the most lethal of human *Plasmodium* parasites [1]. *P. falciparum* has a complex lifecycle characterised by distinctive morphological changes that span the human and mosquito host, with each stage of development supported by versatile biological processes that allow the parasite to adapt to multiple host environments. However, pathologies associated with this disease are entirely attributed to the proliferative asexual development within the human red blood cells (RBCs). During a single replication cycle within the RBCs, parasites undergo pronounced changes over a period of 48 hours which can be roughly divided into three stages: rings (0-20 hours post invasion (h.p.i)), trophozoites (22-38 h.p.i) and schizonts (40-48 h.p.i). The latter stage results in up to 32 daughter merozoites rupturing from a single infected cell, re-entering circulation and propagating the infection [2-4]. Because of the pathologies associated with these RBCs stages, it is not surprising that most treatment efforts focus on this stage.

The global endeavour towards eradication of malaria would be greatly enhanced by access to an effective and affordable vaccine. However, with no such vaccine yet available, prevention and treatment approaches will continue to rely on the use of vector control strategies and chemotherapeutics. Furthermore, eradication from endemic regions is hampered by the emergence of drug resistance to frontline artemisinin-based therapies and partner drugs in the Greater Mekong region of Southeast Asia [5, 6] and, more recently and perhaps most troubling, the emergence of *de novo* artemisinin resistance in Africa [7]. Collectively, this drug-resistance hampers the global progress towards elimination goals and highlights the urgent need for a greater understanding of *P. falciparum* biochemistry to underpin the discovery of new medicines, diagnostics and vaccines.

In recent years, large-scale quantitative proteomics has facilitated the accurate identification of proteins in complex mixtures, examination of alterations in protein expression and abundance, and probing the composition of protein-protein complexes. While other system-wide methods for molecular analysis of *P. falciparum*, such as transcriptomics, have been useful for identifying unique sets of potential molecular targets, mRNA expression often poorly correlates with protein abundance [8-12]. Therefore, direct measurement of protein abundance provides a better representation of the parasite phenotype under given study conditions compared to transcript levels.

Liquid chromatography tandem mass spectrometry (LC-MS/MS)-based quantitative proteomics is the method of choice to measure dynamic changes in global protein levels across biological samples. Data dependent acquisition (DDA) has been extensively used to understand the scope of protein changes across different conditions, whereby the top 10 or 20 precursor MS1 ions detected in the MS for each scan are fragmented to give product ion spectra (MS2), which provides a fingerprint for peptide detection and identification [13, 14]. However, the stochastic nature of precursor selection for

fragmentation leads to inconsistencies and variations in peptide identification between replicates and samples. This becomes particularly problematic for low abundance peptides and reduces the number of proteins that can be accurately quantified [15, 16]. Data-independent acquisition (DIA) is gaining popularity as an alternative data collection method, as MS2 spectra are collected for multiple peptides within a predefined mass-to-charge (*m/z*) range by co-isolating and fragmenting all peptide precursors within the *m/z* range at once [17]. Although the *m/z* range definition may still exclude some peptide populations, DIA ultimately results in extremely high run-to-run reproducibility and a more comprehensive data set over a shorter time period, making it superior to DDA [17-21].

The primary approach for DIA analysis requires prior knowledge of peptide fragmentation stored in spectral ion libraries. Furthermore, as library quality directly influences DIA results, it is important to have a comprehensive and in-depth library that accurately represents the proteome of the organism under investigation [17, 18]. Comprehensive spectral libraries are available for many model organisms [19-21]. However, no such library exists for *P. falciparum*, although such a tool would provide multifaceted support for the identification of much needed drug targets and vaccine candidates. In this study, we produced a comprehensive library obtained from *P. falciparum*-infected RBCs (iRBCs), identifying 3,113 parasite and 1,617 RBC proteins. The spectral library combined with the DIA-MS method was used to perform quantitative analyses of the three distinct asexual RBC stages of the 3D7 wild-type reference line, and the trophozoite stage of artemisinin resistant (Cam3.II^{R539T} and Cam3.II^{C580Y}) and sensitive (Cam3.II^{rev}) lines. The comprehensive library generated in this study combined with the DIA methodology provides an exquisite and valuable resource to address basic biological questions. Drug mode-of-action studies, the identification of novel therapeutic targets, as well as studies aimed at identifying vaccine antigens and diagnostic markers of *P. falciparum* infection will also benefit from this resource.

Data description

Given nearly half of the world's population is at risk of contracting malaria, an in-depth understanding of the proteins involved in disease onset and progression, and how their expression, structure, and function are responsible for disease pathology in the iRBC stage, is critical. Technical advances in proteomics for malaria, such as DIA, are required to fully identify and quantify the entire complement of proteins. DIA analysis requires access to large and comprehensive proteomic datasets. To address these gaps, we generated a comprehensive spectral library from highly synchronised asexual ring-, trophozoite- and schizont-stage parasites of the *P. falciparum* 3D7 reference strain, including the cytosolic fractions from uRBCs and the infected-RBC cytosol (where the parasite exports a large number of its proteins [22]) of trophozoite-iRBCs. Parasites were released from the host RBC using 0.1% saponin, centrifuged and collected as pellets for downstream protein extraction (saponin pellet). The saponin supernatant, containing the soluble cytosolic contents from the host RBC, were incubated with TALON® Metal Affinity Resin to remove haemoglobin prior to protein extraction. Proteins from both the cytosolic and parasite fractions were solubilised and subjected to proteolysis with trypsin. The digested samples were then extensively fractionated using SCX Bond Elut Plexa cartridges and analysed using nanoLC-MS/MS. The raw data were analysed using MaxQuant to generate the spectral library, identifying 3,113 parasite and 1,617 RBC proteins, which was then incorporated into Spectronaut for further application. The spectral library covered 87% of all proteins previously detected in the *P. falciparum* blood stages by mass spectrometry and added a further 692 proteins that were not previously reported with detection at the protein-level in asexual *P. falciparum* parasites.

The spectral library was successfully applied for quantitative analysis of *P. falciparum* proteins. We demonstrated a capacity to quantify 2,063 *P. falciparum* proteins with nearly no missing proteins across ring, trophozoite, and schizont stages of infection using the DIA method. Subsequent enrichment analysis highlighted a plethora of stage-specific functional diversity across blood-stage development. We also utilised the spectral library to compare 2,317 *P. falciparum* proteins between artemisinin resistant (Cam3.II^{R539T} and Cam3.II^{C580Y}) and sensitive (Cam3.II^{rev}) parasites [23]. We confirmed our previous DDA-based quantitative proteomics analysis of these lines [24] and further identified a number of additional parasite proteins enriched in specific dysregulated pathways. Table 1 shows the parasite stage, including hours post RBC invasion, and the number of parasites used for quantitative analysis. This analysis confirmed that the spectral library generated in this work, accompanied by the DIA methodology, can successfully perform reproducible, specific and accurate quantitative proteomics of *P. falciparum* asexual RBC stages and can be used to investigate a wide range of biological questions. Furthermore, the comparative DIA studies will be available as datasets in PlasmoDB [25], to act as reference databases for the worldwide community of malaria researchers.

Table 1. Parasites used for quantitative proteomics applying the *P. falciparum* spectral library and DIA-MS methodology. h.p.i: hours post invasion, HCT: haematocrit.

**Parasite line**	**Stage**	**Number of parasites used**	**Normalised on protein amount**	**µg protein/parasite**
3D7 reference strain	Ring (6-12 h.p.i)	16% parasitaemia, 2% HCT, 45 ml culture	500 µg of protein	69 µg/ 1ml of parasites
3D7 reference strain	Trophozoite (22-28 h.p.i)	8% parasitaemia, 2% HCT, 15 ml culture	500 µg of protein	417 µg/1 ml of parasites
3D7 reference strain	Schizont (38-42 h.p.i)	8% parasitaemia, 2% HCT, 15 ml culture	500 µg of protein	417 µg/1 ml of parasites
Cam3.II^{R539T} Artemisinin resistant [23]	Trophozoite (22-26 h.p.i)	8% parasitaemia, 2% HCT, 15 ml culture	500 µg of protein	417 µg/1 ml of parasites
Cam3.II^{rev} Artemisinin sensitive [23]	Trophozoite (22-26 h.p.i)	8% parasitaemia, 2% HCT, 15 ml culture	500 µg of protein	417 µg/1 ml of parasites
Cam3.II^{C580Y} Artemisinin resistant [23]	Trophozoite (22-26 h.p.i)	8% parasitaemia, 2% HCT, 15 ml culture	500 µg of protein	417 µg/1 ml of parasites

Analyses

Generation of DDA library

Here we present a comprehensive *P. falciparum* asexual RBC stage spectral library to support protein quantification by DIA-MS. The library was generated by combining 56 DDA analyses of peptide samples derived from asexual ring-, trophozoite- and schizont-stage parasites (parasite pellets) and the cytosolic fractions from uRBCs and the RBC compartment of trophozoite-iRBCs. For the cytosolic fractions, haemoglobin was removed using TALON® Resin prior to protein precipitation using TCA and further solubilisation as per Figure 1. The DDA data was collected through extensive peptide fractionation using SCX cartridges and analysed using untargeted nanoLC-MS/MS with reversed phase chromatography and high resolution (Orbitrap) mass spectrometry. The library was generated using MaxQuant (Fig 1). The *P. falciparum* asexual RBC stage spectral library identified 44,449 proteotypic peptides which mapped to 3,113 *P. falciparum* proteins and 1,617 human RBC proteins (Sup data 1).

Properties of the P. falciparum asexual RBC stage spectral library

To demonstrate the proteome coverage of the *P. falciparum* asexual stage spectral library, we compared the proteins included in this library with those in the online reference database, PlasmoDB (release 51) [25] (5,712 protein-encoding genes based on the current genome annotation), and those with evidence of prior detection at the protein level (2,792 protein groups) based on mass spectrometric approaches in the asexual RBC stage [25]. We report that 2,419 of the 3,113 proteins identified in this library have been detected previously, representing 87% of genes previously annotated with evidence of protein-level expression in *P. falciparum*. Importantly, the remaining 692 proteins identified in this library have not been reported previously with mass spectrometry evidence of protein-level expression in asexual stage development (Sup data 2) (Fig 2A). Among these 692 additional proteins, 210 (30%) were identified with one unique peptide, while the rest were identified with two or more unique peptides. The single-hit peptides had high quality MS2 spectra and the same peptide was seen 15 times, on average, in the DDA dataset (minimum 4 times). Furthermore, to show the coverage of each protein detected in the entire library, we calculated the number of proteotypic peptides observed per protein. About 30% of the proteins in the library contained >10 proteotypic peptides, and 85% of these contained at least two proteotypic peptides per protein (Sup data 3) (Fig 2B). Further analysis of proteins identified in this spectral library revealed 579 *P. falciparum* proteins identified from the saponin supernatant of iRBCs, of which only 22 proteins were specific to the RBC cytosol of the iRBCs and not detected in parasite cell pellets (Fig 2C). Out of the 3,091 *P. falciparum* proteins identified from the parasite pellet, 630 came from the ring-stage fraction, 2,674 from the trophozoite-stage fraction, and 2,706 from the schizont-stage fraction, with 607 proteins common between all three stages (Fig 2D). For the uRBC proteome, we identified a total of 1,617 proteins, of which 408 were soluble in the saponin lysate, 478

overlapped between soluble and insoluble fractions, and 731 were unique to the insoluble fraction (Sup data 1) (Sup Fig 1).

It is important to assess the characteristics of this library in comparison to what has been previously published for *P. falciparum* in order to ensure it is well-suited to relevant studies of parasite biology. Therefore, we analysed proteins identified in this spectral library with respect to published mRNA expression (Fig 2E). We analysed four classes of parasite proteins: those in this library and with prior mass spectrometry level evidence in asexual blood stages documented in PlasmoDB (common); proteins absent from this library but with protein-level evidence in PlasmoDB (PlasmoDB unique); proteins in this library that had not previously been identified in asexual RBC stages (library unique); and predicted parasite proteins that are not identified in either this library or in any published asexual RBCs proteomics dataset (not seen) (Fig 2E) [26]. Proteins commonly seen had the highest transcript abundance based on normalised RNAseq counts (FPKM), while proteins unique to either PlasmoDB or this library had lower transcript abundance (Fig 2E). Furthermore, proteins that lacked proteomic-level evidence in asexual stages had the lowest transcript abundance, suggesting that this basal level of transcription does not necessitate translation of detectable amounts of this protein subset in asexual stages, and that expression levels may be higher in other stages of the *P. falciparum* lifecycle. To address this hypothesis, we compared the protein expression of identified (seen) and unidentified (not seen) proteins to previously published mRNA expression data from sexual stages in blood (gametocytes) and mosquito (oocyst and sporozoite). Our results indicate that proteins “not seen” had higher transcript abundance in gametocytes, oocyst and sporozoites compared to asexual stages (Fig 2F) [27, 28], supporting the hypothesis that undetected proteins with low-level mRNA transcription in asexual stages likely have a specific function in sexual stage parasite development. Our study provides clear evidence that ~50% of *P. falciparum* genes are expressed during asexual RBC stages, while the other 50% have a primary function in other life cycle stages.

Accuracy of the asexual spectral library using DIA-MS methodology

To show the application of the *P. falciparum* spectral library using DIA-MS, we prepared ring-, trophozoite- and schizont-stage parasites with a minimum of three biological replicates in experiment 1 and two-three biological replicates in experiment 2 (Fig 3 and Sup Fig 2). Raw DIA files were loaded into Spectronaut^{TM} and processed with its associated default workflow for peptide identification against our spectral library, followed by protein normalisation and quantification. In total, more than 2,000 proteins (2,064 proteins for experiment 1 (Sup data 4); 2,120 proteins for experiment 2 (Sup data 5)) were quantified in each of the distinct stages of the parasite using the *P. falciparum* library (Fig 3 and Sup Fig 2). For comparison, the raw DIA files from experiment 1 were also analysed using a spectral library-free DIA “DirectDIA” approach, which identified a significantly lower number of proteotypic peptides (14,639 peptides) than the spectral library search (33,731 peptides) resulting in increased

coefficient of variation (CV) between samples. This improved precision and proteome coverage from spectral library-based DIA compared to a library-free approach has been observed in previous studies [29, 30]. The DirectDIA approach was therefore not used for identification and quantification of proteins.

To evaluate the quality of the *P. falciparum* library-based analysis, we compared protein abundances of 1,990 common proteins (proteins without missing values) across the two different experiments (Sup data 6 and Fig 4). Pearson's correlation coefficients between experiment 1 and experiment 2 were 0.7 for rings, 0.5 for trophozoites and 0.6 for schizonts (Fig 4A). Heatmap analysis of protein expression across the three stages for the two experiments indicated that the expression patterns of the proteins are reproducible (Fig 4B). To further determine quantitative reproducibility, we computed the CV in each specific asexual stage across both experiments. For all asexual specific stages, the median CVs of protein abundances were below 10% (Fig 4C) with the exception of the ring-stage sample in experiment 2 (CV=17.8%). Collectively, the *P. falciparum* asexual library-based analysis using our DIA-MS exhibited excellent reproducibility.

Hierarchical clustering confirms the good agreement between biological replicates and shows a clear distinction of expression pattern between the three asexual stages (Fig 3, Sup Fig 2, and Sup Fig 3). Heatmap analysis shows that trophozoite and schizont stages are very similar to one another, but quite distinct from the early ring-stage parasites (Fig 3, Sup Fig 2, and Sup Fig 3). Analysis of proteins differentially expressed between these distinct stages (that had a raw p-value of < 0.05), including proteins enriched from ring- to trophozoite-stage, trophozoite- to schizont-stage and finally schizont- to ring-stage, revealed sets of proteins specific to each of these stage transitions (Fig 5). This analysis of the expression pattern of proteins across the stages was further confirmed and compared to experiment two and another experiment (experiment three, Sup data 7), which only contained the comparison of ring- to schizont- stage parasites (Sup Fig 2; trophozoite-stage parasite data was not acquired). Enrichment analysis showed a distinct clustering of GO biological processes across each of the three developmental stages (Sup data 8) (Fig 5). Comparison of the ring- to trophozoite-stage transition displayed a range of significantly over-represented pathways ($p \leq 0.05$), with the most significant clustered processes enriched in regulation of host-cell entry and protein transport-related terms in rings, which collectively describes establishment of active infection within a new host RBC. In contrast, both trophozoite to schizont- and schizont- to ring-transitions display a more specific clustering of enriched pathways; ribosomal biogenesis (up-regulated in trophozoites) is the most representative cluster in trophozoite- to schizont-stage, whereas processes relating to cell division (up-regulated in schizonts) are highly represented in schizont- to ring-stage parasites, consistent with the replicative end point of asexual development (Fig 5).

The common reference strain of *P. falciparum,* 3D7 wildtype strain, was used for the generation of this spectral library. In order to test if this DIA-MS library can be used for other *P. falciparum* strains, we performed quantitative DIA-MS on 500 µg of protein lysate from the artemisinin resistant Kelch-13-mutant Cambodian isolate, Cam3.II^{R539T}, and the related artemisinin sensitive Cam3.II^{rev} line. We identified a total of 2,317 *P. falciparum* proteins in all samples (Sup data 9). Heatmap and volcano plot analysis of all identified proteins demonstrated a greater number of proteins that were dysregulated between the two lines compared to previous proteomics analyses using a DDA-based approach (Fig 6A and B) [24]. The abundance of one of the dysregulated proteins, Kelch13 (Pf3D7_1343700) was found to be two-fold lower in artemisinin-resistant Cam3.II^{R539T} parasites compared to artemisinin-sensitive Cam3.II^{rev} (Fig 6C) consistent with previous studies [24, 31, 32]. One sample from the related artemisinin resistant line bearing a different mutation in Kelch13, Cam3.II^{C580Y}, was also analysed to confirm proteins that were differentially dysregulated in artemisinin-resistant parasites compared to Kelch13 wildtype (Cam3.II^{rev}) parasites. Detailed analysis of GO term enrichment from dysregulated proteins identified entry into host cell and vesicular trafficking (<0.006 p-value) to be overrepresented in proteins significantly dysregulated (>0.05 p-value) (Fig 7), suggesting a role for these processes in the mechanism of artemisinin resistance (Sup data 10).

Discussion

P. falciparum continues to cause the most severe form of malaria in humans. Despite years of study into the basic biochemical and molecular biology of these complex parasites, many questions remain unanswered. Data-dependent mass-spectrometry based proteomics (DDA-MS) has been used extensively to reveal important features of the parasite's regulatory mechanisms. However, there are many issues with DDA-MS, including run-to-run reproducibility and identification of less abundant proteins. Therefore, a relatively high amount of starting material and extensive sample fractionation is required to generate high quality proteomics data with reasonable depth of coverage. This is evident from our DDA-MS of each of the distinct stages, and also from previous publications, where 1-2 mg of starting material was used followed by extensive fractionation [24, 33]. In comparison, many of the DDA-MS problems can be circumvented using a data-independent acquisition (DIA-MS) methodology, which requires 2-4 times less biomass of starting material (500 µg) and avoids the need for fractionation (Fig 1B).

Another benefit of generating a spectral library rather than using traditional DDA-MS is reproducibility and run-to-run consistency in regards to the identification of peptides and proteins. This is shown in our DDA-MS analysis of ring-, trophozoite- and schizont-stage parasites for spectral library generation, where only 607 proteins were found to be common to all three stages (Fig 2D). This is further supported by many published proteomics analyses of these three distinct stages, where 600-700 proteins were commonly identified [31, 34-36], and proteomics analysis of one distinct stage with different treatment conditions where 600-1,000 proteins were identified [37, 38]. In contrast, with DIA-MS, the overlap of proteins detected across the three stages are frequently around the order of 2,000 *P. falciparum* proteins - three times more than in DDA-MS analysis (Fig 4C, Supp Fig 2A and C). Previous DDA-MS of artemisinin resistant and sensitive parasites had identified 2,824 proteins. However, due to the nature of DDA-MS, only 520 proteins were included in the final analysis as the dataset contained many missing values [24]. Using DIA-MS methods in this study to analyse these same resistant and sensitive parasites led to the reproducible identification of 2,317 *P. falciparum* proteins across all samples, again demonstrating the benefits of DIA-MS using this spectral library compared to a typical DDA-MS. We were also able to demonstrate that DIA-MS experiments analysed by mass spectrometry on different days were generally reproducible (Fig 4A and B), and the median CVs of protein abundances within each experiment was less than 10%, with the exception of ring-stage proteomics from experiment 2 (Fig 4C). Saponin lysis of mature iRBCs (trophozoite and schizonts) is more reproducible compared to ring-stage parasites, with a number of metabolomics and proteomic studies of this early stage demonstrating the heavy influence of host metabolites and proteins, subsequently contributing to the variability between samples [24, 39]. Despite this, we were still able to demonstrate that DIA-MS using the spectral library very clearly outperforms DDA-MS when it comes to reproducibility and run-to-run identification.

In quantitative proteomic studies, high quantification reproducibility is of utmost importance. Therefore, for the majority of DDA-based proteomics studies of *P. falciparum* asexual stages, labelling of peptides is usually necessary [24, 31, 37]. Labelling approaches generally limit the number of samples that can be included in a single study, and are expensive and time consuming, while DIA-MS allows for label-free protein quantification across the entire proteome with quantification performance comparable to labelling methods [40]. This was evident from the DIA-MS of the resistant and sensitive parasites, where we identified Kelch13 to be decreased in abundance in artemisinin resistant parasites, with comparable fold changes to those previously shown using peptide labelling [24] (Fig 6C). Furthermore, our previous quantitative analysis of artemisinin resistant and sensitive parasites only identified Kelch13 to be dysregulated [24]. However, the DIA-MS demonstrated a larger number of proteins to be dysregulated between the two lines (Fig 6). Enrichment analysis of significantly dysregulated proteins revealed two important processes - entry into host and vesicular mediated transport (Fig 7). Protein transport (down-regulated in artemisinin resistant parasites) is of particular interest since Kelch13 has been shown to interact with a number of proteins involved in vesicular trafficking, more specifically Kelch13 is shown to be involved in endocytosis of host haemoglobin [32, 41]. Mutations in Kelch13 alters haemoglobin uptake within the parasite, and the mechanism by which the parasite internalises and transforms haemoglobin into haemozoin is central to artemisinin activation and efficacy [32, 41]. Therefore, a lack of haemoglobin uptake would result in increased artemisinin tolerance. Enrichment of the process, entry into host cell (down-regulated in artemisinin resistant parasites) is surprising and could be a secondary stress response of the resistant parasites by dysregulating the expression of invasion proteins. Previous studies have demonstrated drug treated trophozoite-stage parasites to have dysregulated proteins involved in invasion [33, 37]. Another possibility for the enrichment of invasion proteins could be related to the role of Kelch13 in vesicular trafficking. Invasion proteins are sorted into secretory organelles, which are endosomal like structures, and it is possible that mutation of Kelch13 affects either biogenesis of secretory organelles or sorting of invasion proteins into these organelles [42, 43].

Access to a detailed spectral library is critical for understanding the mechanisms by which *P. falciparum* parasites regulate their development, and identifying proteins important for each specific stage is a key element towards a rational design of agents for prophylaxis and treatment of malaria. Our study demonstrated that by combining this spectral library with the DIA-MS approach, we were able to add critical information about proteins expressed in the three distinct asexual blood stages of the *P. falciparum* parasite. Previously, quantitative analysis of these three distinct stages using DDA analysis demonstrated that there is a large proportion of proteins (54% of identified proteins) exhibiting variable expression across these stages [34]. However, this DIA-based analysis demonstrated that trophozoite- to schizont-stage parasite proteins are similar in expression, while ring- to trophozoite- or schizont-stage are very different (Fig 3, Sup Fig 2 and Sup Fig 3). Analysis of the differences between

ring and trophozoite stages enriched for parasite proteins predominantly involved in host cell invasion and protein sorting (vesicular trafficking) (Fig 5). This is expected as these proteins are necessary for parasite invasion and establishment of a niche within the host RBC that will allow for the uptake and utilization of nutrients from the extracellular environment [44, 45]. Trophozoite-stage parasites are known to be the most metabolically active stage of development [46-48] and the enrichment of ribosomal biogenesis aligns with an active synthesis of proteins to kick-start metabolism. As the parasite ages to a schizont, the enrichment of cell division proteins is in preparation for mitotic production of daughter cells [49] (Fig 5). These daughter cells, upon rupture of the infected cell, re-invade naïve RBCs to propagate infection.

It is well known that there is little correlation between mRNA and protein abundances in a number of systems, as a result of post-transcriptional and post-translational regulatory mechanisms. These mechanisms have also been shown to be important in regulation of gene expression in the asexual stages of *P. falciparum* parasites [50, 51]. We were interested in testing this observation using the quantitative analysis of the three distinct stages by comparing proteins in this library to published mRNA expression of the same stages (Sup data 11) [26]. As per previous publications, our analysis showed that there is little to no correlation between protein and mRNA abundance (0.2 for rings, 0.5 for trophozoites and 0.3 for schizonts) (Fig 8A and B). Furthermore, translational repression is a common regulatory mechanism used by these parasites for an effective transition to other stages, which has been clearly demonstrated for gametocytes and sporozoites, where after fully maturing and upon successful transmission, they rapidly translate available mRNA [52]. Importantly, it is yet to be shown that translational repression is used in *P. falciparum* asexual stages and more work is warranted to resolve the role of these control mechanisms in this stage of the parasite's life cycle.

Spectral library searching, as opposed to sequence searching via *in silico* predicted fragmentation spectra, has greater sensitivity for peptide ions included in the library; however, relatively few DDA datasets are analysed in this way. A major concern is the incompleteness of the spectral library and in order to address this issue, we analysed the completeness of the generated library by comparing it to the database of the *P. falciparum* genome from PlasmoDB. We found that this comprehensive library has 87% coverage of all previously reported *P. falciparum* proteins with protein-level evidence detected by mass spectrometry, the other 13% missing from this library were only identified following specific peptide enrichment procedures, such as phosphoproteomics [25]. This library also added a further 692 proteins to the genes detectable at the protein-level in asexual *P. falciparum* parasites (Fig 2A). Analysis of mRNA expression for proteins not seen in our library suggested that they may be expressed in other stages of the *P. falciparum* life cycle, such as gametocytes (sexual stage of the parasite), oocyst and sporozoites (mosquito stages of the parasite) [27, 28] (Fig 2F). This demonstrates that this spectral library will be advantageous for the malaria community only for the analysis of the asexual stages, and

further DDA proteomics data from other stages is required for the expansion of the library to cover additional stages of the lifecycle.

In addition to the spectral library of the parasite, a library of the host RBC cytosol was also established. *P. falciparum* heavily modifies its host cell, and although much is known about modification of the host cell membrane, export of parasite proteins in the RBC cytosol and modification of RBC cytosolic proteins (such as phosphorylation [53]) remains to be further explored. This spectral library sets the tone for further investigations in host-parasite interactions, and opens the door to host-directed therapy approaches.

An important consideration for the use of public libraries will be the portability of information between instrument types and laboratories. For example, can the DDA spectral library generated on a specific instrument platform be used to analyse DIA-MS data on a different platform from laboratories around the world? Although the best comparability is achieved when fragment ion spectra are generated on the same instrument platform [54], fragment ion spectra acquired from instruments using “beam type” collision-induced dissociation such as QTOFs and Q-Orbitrap [55] (used in this study) are sufficiently comparable for their effective use as prior knowledge in a peptide-centric analysis [56]. In regards to chromatographic information, due to the addition of iRT peptides in the samples, the accuracy of such methods is generally tolerant to changes in gradient length and column dimensions; however, some misalignment will result from changes in other factors such as mobile/stationary phases or column temperature, as they can affect peptide elution [57]. This suggests that our spectral library should provide a useful DIA reference library for most LC-MS/MS platforms with “beam type” fragmentation, but reproducibility for optimal DIA-MS analysis will be greatest when using a quadrupole-Orbitrap instrument with HCD fragmentation operating under the same LC-MS/MS conditions described here.

In conclusion, we have generated a comprehensive asexual stage spectral library and demonstrated that it can be successfully applied for consistent quantification of >2,000 *P. falciparum* proteins using a DIA-MS analysis pipeline. We showed that quantitative analysis is effective across different *P. falciparum* strains, but is specific to the asexual blood stages of the parasite. Our freely accessible library will furnish the research malaria community with a resource to explore future proteome-phenotypic studies with the advantage of robustness, reproducibility and streamlined procedures.

Potential implications

Proteome spectral libraries are available for a number of model organisms, such as yeast and humans. Here, we provide the first mass spectrometry-based proteome spectral library for *P. falciparum*, the most lethal of the malaria parasite that infects humans. In this study, we demonstrated how this library can facilitate DIA-MS proteomics analysis to understand basic biological processes of the parasite in distinct stages and can contribute to understanding drug resistance mechanisms in these parasites. Furthermore, the datasets generated in this study for the three distinct asexual blood stages will act as reference points for malaria researchers wishing to understand the expression profile of their protein of interest across the stages. From our successful application, we can be certain that spectral library-based quantitative DIA-MS will usher in a new wave of proteomics studies in the malaria research community across a wide range of applications, including in molecular classification, biomarker discovery, analysis of pathogenesis pathways, drug and vaccine discovery, and unravelling mechanisms of therapy response and resistance. The robustness and efficiency of DIA-MS will also overtake other semi-quantitative methods as the go-to method for measurement of protein abundances, allowing routine measurement of ~2,000 proteins rather than single-protein measurements provided by antibody-based approaches.

Methods

P. falciparum spectral library generation using parasite pellets

Asexual *P. falciparum* (3D7 reference strain, Cam3.II^{R539T}, Cam3.II^{C580Y}, and Cam3.II^{rev}) were cultured as per standard methods [58], with minor adjustments [59]. Briefly, cultures were maintained using O^{+} human RBCs (Australian Red Cross Blood Service) at 2% or 4% haematocrit (HCT) in modified RPMI 1640 medium containing hypoxanthine and 0.5% (w/v) Albumax II (Gibco) at 37°C under defined atmospheric conditions (95% N_2, 4% CO_2, 1% O_2).

To achieve tightly synchronous cultures for library generation, *P. falciparum* cultures with a high proportion of ring-stage parasites (30 mL, 10-12% parasitaemia, 2% HCT) were synchronised by performing sorbitol lysis twice, 10 h apart. For schizont library generation, parasites were harvested 27 h after the second sorbitol lysis. At this time point, the cultures contained a high proportion of segmented schizonts and a small proportion of young ring-stage parasites (<10%). For ring library generation, parasites were harvested in the second cycle 16-18 h post-infection (h.p.i), while trophozoites were harvested 28-30 h.p.i. Proteomic samples were prepared as previously described with minor modifications [24, 60]. For generating stage-specific samples, iRBCs were pelleted by centrifugation (650 × *g*, 5 min) and parasites were isolated from RBCs by resuspending in saponin lysis buffer (0.1% w/v in phosphate buffered saline (PBS)) containing protease and phosphatase inhibitors (PPI); 1 × complete mini protease inhibitor cocktail (Roche), 20 mM sodium fluoride and 0.1 mM sodium orthovanadate) and incubated for 10 mins on ice. Isolated parasites were pelleted (4,000 × *g*, 7 min) and washed (15,850 × *g*, 3 min, supernatant discarded after each wash) a total of three times in 1 mL 1 × PBS with PPI.

P. falciparum library generation using cytosolic uRBC and iRBC fractions

Synchronous cultures were obtained with a double sorbitol lysis (6 h interval). At ~30 h.p.i, iRBCs were collected by magnet purification [61], quantified using a Neubauer hemocytometer, and 10^9 iRBCs were aliquoted in microtubes. As a control, 10^9 uRBCs from the same donor were also aliquoted into microfuge tubes. This was performed 10 times (using RBCs from different donors) and the protein samples were pooled together to achieve 10^{10} uRBCs and 10^{10} iRBCs.

uRBCs and iRBCs were lysed for 10 min on ice in 600 µl saponin lysis buffer (as described above). After centrifugation (16,200 × *g*, 5 min, 4°C), the supernatant, containing cytosolic fraction of the RBCs, was carefully collected and loaded onto 600 µl of TALON® Metal Affinity Resin slurry (Takara) and washed with an equal volume of 1× PBS with PPI (600 × *g*, 2min) to remove haemoglobin. Samples were incubated with the resin for 10 min on a rotating wheel at 4°C, and the haemoglobin-free supernatant was collected (2 min at 600 × *g*; 2 min at 2,400 × *g*, transferring the supernatant to fresh pre-chilled microfuge tubes after each wash). Ice-cold trichloroacetic acid (TCA) was added to samples

(1:20 of final volume) and incubated for 10 min on ice to facilitate protein precipitation. The pellet was washed in 1 ml acetone (16,200 × *g*, 3 min), the acetone removed and evaporated, before samples were stored at -80°C until required.

Sample collection for quantitative DIA-MS using *P. falciparum* spectral library

To achieve tightly synchronous cultures for quantitative DIA-MS analysis, late schizont-stage parasites were enriched by magnet purification [61]. The highly enriched parasite fraction was then added to fresh uRBCs (2% HCT) and left to invade for 3 h before sorbitol synchronisation. For 3D7 parasites, the parasitaemia was adjusted to 16% and 45 mL was used to prepare rings samples (6 h.p.i). For trophozoite and schizont stages, 15 mL of the same sample were adjusted to 8% parasitaemia and samples prepared at 22-28 h.p.i and 38-42 h.p.i, respectively. For artemisinin resistant (Cam3.II^{R539T}, Cam3.II^{C580Y}) and sensitive (Cam3.II^{rev}) parasites, the parasitaemia was adjusted to 8%, and 15 mL of trophozoite-stage parasites at 22-26 h.p.i were collected.

Sample preparation for *P. falciparum* spectral library

Saponin pellets were solubilized with SDC lysis buffer (100 mM 4-(2-hydroxyethyl)-1-piperazineethanesulfonic acid (HEPES), 1% sodium deoxycholate (SDC), pH 8.1) supplemented with PPI and probe sonicated with × 3 pulses at 30 secs each. Following sonication, samples were boiled at 95°C for 5 mins, allowed to return to room temperature (RT), before reducing and alkylating with tris(2-carboxyethyl) phosphine (TCEP) (10mM final) and iodoacetamide (40 mM final) at 95°C for 5 mins. After returning to RT, proteins were precipitated using ice cold TCA and pellets were resuspended in SDC lysis buffer without PPI and sonicated to aid protein solubilisation. Protein concentration was measured using the Pierce bicinchoninic acid (BCA) protein assay and samples adjusted to 3-5 mg of protein per sample. Trypsin (1:50; Promega) was added and samples were incubated 16 h at 37°C with constant agitation at 1500 rpm in a Multi-Therm™ (Benchmark Scientific). On the following day, trypsin activity was quenched using 5% (v/v) formic acid (FA), before adding 100% (v/v) ethyl acetate to remove detergent. The samples were centrifuged at 4000 × *g* for 5 mins and the top layer of supernatant was removed. Samples were dried using CentriVap Benchtop Centrifugal Vacuum Concentrator (Labconco) at 100 mbar and 37 °C for 10 mins to remove excess ethyl acetate. The samples were then diluted five-fold and loaded onto a SCX Bond Elut Plexa (Agilent) and eluted into 12 fractions as described previously [24]. Peptides were then eluted from SCX cartridges using 500 µl of elution buffers at a rate of one drop/sec. Elution buffers consisted of increasing concentrations of ammonium acetate (Sigma-Aldrich) (75 mM, 100 mM, 125 mM, 150 mM, 175 mM, 225 mM, 250 mM, 275 mM, 300 mM, 325 mM, and 350 mM) with 20% (v/v) acetonitrile (ACN) and 0.5% (v/v) FA. The final elution buffer consisted of 80% ACN and 5% ammonium hydroxide (Sigma-Aldrich) to remove any remaining bound peptides. Eluates (fractions) were semi-dried to remove most of the ACN and then subjected to desalting using in-house generated StageTips as described previously [62]. The desalted

fractions were dried to completion and reconstituted in 20 µl of 2% ACN and 0.1% FA, sonicated for 15 mins and subject to automatic vortexing for a further 15 mins to allow complete resuspension of peptides. To facilitate retention-time alignments among samples, a retention-time kit (iRT kit, Biognosys, GmbH) was spiked at a concentration of 1:20 (v/v) for all fractions [63]. Samples were stored at -80°C and the particle free supernatant was transferred to LC-MS vials immediately prior to LC-MS/MS analysis.

Sample preparation for quantitative DIA-MS

Sample preparation for DIA-MS was mostly the same as sample preparation for spectral library generation with minor modifications. Significantly less protein material was digested overnight with trypsin, 500 µg compared to 3-5 mg. On the following day, the peptide samples were not subjected to SCX fractionation, but rather directly desalted using in-house generated StageTips as described previously [62]. The desalted peptide samples were then dried to completion and reconstituted in 20 µl of 2% ACN and 0.1% FA with iRT peptides [63]. Samples were stored at -80°C and the particle free supernatant was transferred to LC-MS vials immediately prior to LC-MS/MS analysis.

Mass spectrometric instrumentation and data acquisition

For DDA acquisition, NanoLC-MS/MS was carried out on a Q Exactive HF Orbitrap LC-MS/MS system as described previously [24], with minor modifications. Samples were loaded at a flow rate of 15 µL/min onto a reversed-phase trap column (100 µm × 2 cm), Acclaim PepMap media (Dionex) and maintained at a temperature of 40 °C. Peptides were eluted from the trap column at a flow rate of 0.25 µL/min through a reversed-phase capillary column (75 µm × 50 cm) (LC Packings, Dionex). For acquisition by HPLC, a 158 min gradient was set using an incremental gradient that reached 30% ACN after 123 min, 34% ACN after 126 min, 79.2% ACN after 131 min and 2% ACN after 138 min for a further 20 min. The mass spectrometer was operated in a data-dependent mode with each cycle comprising of one full scan performed at 70,000 resolution (AGC target of $3e^6$, mass range of 375-1575 *m/z* and maximum injection time of 54 ms) The 20 most intense precursors with charge states 2-6 were selected for fragmentation at 17,500 resolution (AGC target of $5e^5$, normalised collision energy of 27.0, activation time of 15 ms, isolation window of 1.8 *m/z*, maximum injection time of 118 ms and dynamic exclusion enabled). For DIA, a 25-fixed-window setup of 24 *m/z* effective precursor isolation over the *m/z* range of 376-967 Da was applied. Full scan was the same as DDA mode with fragmentation resolution at 17,500 (AGC target of $2e^5$, maximum injection time at auto, normalised collision energy of 27).

Shotgun data searching and spectral library generation

DDA files were searched against *P. falciparum* (UP000001450, release version 2016_04) and *Homo sapiens* (UP000005640, release version 2017_05) UniProt FASTA databases and the Biognosys iRT

peptides database. The number of entries in the database actually searched were 3,970,852 with trypsin as enzyme specificity and 2 missed cleavages were permitted. Carbamidomethylation of cysteines was set as a fixed modification. Oxidation of methionine and protein N-terminal acetylation were set as variable modifications. Parent mass error tolerance and fragment mass tolerance were set to 20 ppm. For both peptide and protein identification, a false discovery rate (FDR) of 1% was used. MaxQuant search results were imported as spectral libraries into Spectronaut using default settings. MaxQuant output files were obtained and imported into Spectranout and iRT values were computed using the linear iRT regression function embedded in Spectranout. A consensus library was generated for *P. falciparum* iRBCs and saved for downstream targeted analysis. The consensus library contained 44,449 peptides corresponding to 4,730 proteins.

Spectronaut targeted data extraction

Raw files were processed using Spectronaut^{TM} (version 13.0) against the in-house generated *P. falciparum* spectral library. For processing, raw files were loaded in Spectronaut, the ideal mass tolerances for data extraction and scoring were calculated on its extensive mass calibration with a correction factor of 1. Both at precursor and fragment level, the highest data-point within the selected *m/z* tolerance was chosen. Identification of peptides against the library was based on default Spectronaut settings (Manual for Spectronaut 13.0, available on Biognosis website). Briefly, precursor Qvalue Cut-off and Protein Qvalue Cut-off were as per default at 1% and therefore only those that passed this cut-off were considered as identified and used for subsequent processing. Retention time (RT) prediction type was set to dynamic indexed RT. Interference correction was performed at the MS2 level. For quantification, interference correction was activated and cross run normalisation was performed using the total peak area at a significance threshold of 0.01. Fold-changes for the ring-stage versus trophozoite-stage and schizont-stage were calculated in Microsoft Excel and p-values were calculated using a standard Student's t-test. Volcano plots and hierarchical clustering is performed in Metaboanalyst [64]. Hierarchical clustering analysis was carried on two sets, namely, all identified P. *falciparum* proteins, and 400 differentially regulated proteins.

Comparative analysis of protein to mRNA expression

The public transcriptomics data for the same developmental stages of *P. falciparum* from Toenhanke *et.al.* [26] was obtained from GEO (Accession: GSE104075). Counts data was generated by mapping to the reference genome of *P. falciparum* (P3D7-release-39) using RNAsik pipeline implemented in the Laxy platform (https://doi.org/10.5281/zenodo.3767371). The transcriptomics data was then CPM normalised in Degust Software. The comparative analysis was carried out in R for common gene/protein expressions found in both transcriptomics and proteomics data. First, the average expression of each protein was calculated for each developmental stage, which was then used to calculate Pearson's correlation coefficient between transcript and protein abundance.

GO Enrichment Analysis

Protein abundance values were log-transformed and subjected to pairwise Student's t-tests to assess differences in abundance between ring-, trophozoite- and schizont-stages, or between Cam3.II^{R539T} and Cam3.II^{rev} parasites. The resulting p-values were used as protein scores in a GO enrichment analysis using topGO, using the classic algorithm and ks statistic to assess GO-term enrichment.

Over-represented GO terms extracted were imported into the *Reduce and Visualize Gene Ontology (REVIGO)* web server [65] using default parameters with *P. falciparum* as the chosen database for term size. The resulting output file containing summarized GO terms (redundant terms removed) was visualized in Cytoscape (v3.8.0) [66]. Nodes were sized according to GO term uniqueness (i.e. fewer redundant terms merged with more general, higher-order terms). Nodes were coloured by fold-change up (red) or down (blue) when compared between lifecycle stages or resistant versus sensitive parasites.

Data availability

All the raw data (DDA) and search result files (MaxQuant excel output) used to generate the *P. falciparum* spectral library have been deposited in the ProteomeXchange Consortium through the PRIDE partner repository [67] with identifier PXD027241.

The raw data (DIA-MS) files generated in this study for quantitative analysis and their Spectronaut protein intensity have been deposited in the ProteomeXchange Consortium through the PRIDE partner repository [67] with identifier PXD027301.

Other data further supporting this work are openly available in the *GigaScience* repository, GigaDB [68].

Supplementary data files

Venn diagrams depicting overlaps of identified proteins by data-dependent acquisition; Venn diagram comparison and hierarchical cluster analysis of differentially expressed proteins; Volcano plot of differential protein abundance from 2064 identified *P. falciparum* proteins;

Supplementary_data_sheet_1-Intensities of proteins identified in the spectral library. Uniprot IDs for *H. sapiens* proteins and PlasmoDB IDs for *P. falciparum* proteins with their corresponding gene names are provided. For each gene, the relative protein intensities measured using a DDA approach in different stages of the parasite red blood cell stage (ring-, trophozoite-, and schizont-stage) and supernatant of infected cell is also provided.

Supplementary_data_sheet_2-Comparison of *P. falciparum* proteins in this Spectral library to PlasmoDB. Annotated as “common” are proteins that are identified in this spectral library and have mass spectrometry evidence in the red blood cell stage of infection from PlasmoDB. Annotated as “not seen” are proteins not identified in this spectral library and also have no mass spectrometry evidence of being present in the red blood cell stage of infection. Annotated as “PlasmoDB-unique” are proteins that have mass spectrometry evidence in the red blood cell stage of infection, but were not detected in this spectral library. Annotated as “spectral-library-unique” are proteins that have no mass spectrometry evidence in the red blood cell stage of infection from PlasmoDB, but were identified in this spectral library.

Supplementary_data_sheet_3-Number of proteolytic peptides per protein of *H. sapiens* and *P. falciparum* identified in this Spectral library.

Supplementary_data_sheet_4-Protein intensities for DIA experiment 1 – Ring- Vs Trophozoite- Vs Schizont-stage parasites with three biological replicates per condition.

Supplementary_data_sheet_5-Protein intensities for DIA experiment 2 – Ring- Vs Trophozoite- Vs Schizont-stage parasites, with two-three biological replicates per condition.

Supplementary_data_sheet_6-Comparison of commonly identified *P. falciparum* protein expression of Exp 1 and Exp 2.

Supplementary_data_sheet_7-Protein intensities for DIA experiment 3 – Ring- Vs Schizont-stage parasites with a minimum of three biological replicates per condition.

Supplementary_data_sheet_8-GOterms (with protein IDs in GOterm) of differentially expressed proteins. Annotated as "rings to trophozoites enrichment" are GOterms enriched in ring-stage parasites compared to trophozoites. Annotated as "schizonts to rings enrichment" are GOterms enriched in schizont-stage parasites compared to rings. Annotated as "trophozoites to schizonts enrichment" are GOterms enriched in trophozoite-stage parasites compared to schizonts.

Supplementary_data_sheet_9-Protein intensities for DIA experiments - Artemisinin resistant (Cam3.II^{R539T}) Vs sensitive (Cam3.II^{rev}).

Supplementary_data_sheet_10-GOterms (with protein IDs in GOterm) of differentially expressed proteins in artemisinin resistant line (Cam3.II^{R539T}) compared to sensitive (Cam3.II^{rev}).

Supplementary_data_sheet_11-Comparison of protein expression (common proteins identified from Exp 1 and Exp 2) from the three distinct stages of the red blood cell infection to published RNA levels.

List of abbreviations

DDA: data dependent acquisition; RBC: red blood cells; iRBC: infected red blood cells; DIA: data-independent acquisition; uRBC: uninfected red blood cells; h.p.i: hours post invasion; ART: artemisinin; LC-MS/MS: liquid chromatography tandem mass spectrometry; *m/z*: mass-to-charge; Kelch13: PfKelch13; MS: mass spectrometry; CV: coefficient variation; PPI: protease and phosphatase inhibitors; *P. falciparum*: *Plasmodium falciparum*; TCA: trichloroacetic acid; formic acid: F.A; acetonitrile: ACN.

Competing interests

The authors declare that they have no competing interests.

Authors' contributions

G.S, A.D., A.E.S., C.B. performed the experiments. G.S. processed the data and analysed the results. G.S., C.A.M., A.D.S. and M.B.B. conducted further analysis of the results. G.S., A.D., A.E.S., C.B., C.A.M., A.D.S., M.B.B., R.B.S., drafted the manuscript. T.G.C. and D.J.C. supervised the study. All authors revised and approved the final version of the manuscript.

Acknowledgements

The authors thank Professor David Fidock (Columbia University) for provision of the genetically modified Cam3.II^{R539T}, Cam3.II^{C580Y} and Cam3.II^{rev} *P. falciparum* isolates. The Monash Proteomics and Metabolomics Facility (Parkville Node) provided technical assistance with metabolomics and proteomics experiments. The Australian Red Cross Blood Service in Melbourne donated human red blood cells for *in vitro* parasite cultivation (Deed 19-10VIC-06). At La Trobe University, use of human erythrocytes was approved by the La Trobe University Research Ethics Committee (ethics number HEC17- 013) and an Australian Red Cross Blood Service Agreement (Deed 19-05VIC-01). Funding support was provided by the Australian National Health and Medical Research Council (NHMRC) project grants #APP1128003 and #APP1160705 and fellowship to D.J.C. (#APP1148700). We also wish to acknowledge the traditional owners of the lands on which this project was conducted, the Wurundjeri People of the Kulin nation.

References

1. WHO. *World Malaria Report 2019*. 2019. World Health Organization, Geneva.
2. Gilles HM. Bruce-Chwatt's essential malariology. 3rd ed. / Herbert M. Gilles, David A. Warrell. ed. London: London : E. Arnold; 1993.
3. Cowman AF and Crabb BS. Invasion of red blood cells by malaria parasites. Cell. 2006;124 4:755-66. doi:10.1016/j.cell.2006.02.006.
4. Abkarian M, Massiera G, Berry L, Roques M and Braun-Breton C. A novel mechanism for egress of malarial parasites from red blood cells. Blood. 2011;117 15:4118-24. doi:10.1182/blood-2010-08-299883.
5. Amato R, Pearson RD, Almagro-Garcia J, Amaratunga C, Lim P, Suon S, et al. Origins of the current outbreak of multidrug-resistant malaria in southeast Asia: a retrospective genetic study. Lancet Infect Dis. 2018;18 3:337-45. doi:10.1016/S1473-3099(18)30068-9.
6. Imwong M, Hien TT, Thuy-Nhien NT, Dondorp AM and White NJ. Spread of a single multidrug resistant malaria parasite lineage (PfPailin) to Vietnam. Lancet Infect Dis. 2017;17 10:1022-3. doi:10.1016/S1473-3099(17)30524-8.
7. Uwimana A, Legrand E, Stokes BH, Ndikumana JM, Warsame M, Umulisa N, et al. Emergence and clonal expansion of in vitro artemisinin-resistant *Plasmodium falciparum* kelch13 R561H mutant parasites in Rwanda. Nat Med. 2020;26 10:1602-8. doi:10.1038/s41591-020-1005-2.
8. Ideker T, Thorsson V, Ranish JA, Christmas R, Buhler J, Eng JK, et al. Integrated genomic and proteomic analyses of a systematically perturbed metabolic network. Science. 2001;292 5518:929-34. doi:10.1126/science.292.5518.929.
9. Griffin TJ, Gygi SP, Ideker T, Rist B, Eng J, Hood L, et al. Complementary profiling of gene expression at the transcriptome and proteome levels in Saccharomyces cerevisiae. Mol Cell Proteomics. 2002;1 4:323-33. doi:10.1074/mcp.m200001-mcp200.
10. Baliga NS, Pan M, Goo YA, Yi EC, Goodlett DR, Dimitrov K, et al. Coordinate regulation of energy transduction modules in Halobacterium sp. analyzed by a global systems approach. Proceedings of the National Academy of Sciences. 2002;99 23:14913. doi:10.1073/pnas.192558999.
11. Gygi SP, Rist B, Gerber SA, Turecek F, Gelb MH and Aebersold R. Quantitative analysis of complex protein mixtures using isotope-coded affinity tags. Nat Biotechnol. 1999;17 10:994-9. doi:10.1038/13690.
12. Foth BJ, Zhang N, Chaal BK, Sze SK, Preiser PR and Bozdech Z. Quantitative time-course profiling of parasite and host cell proteins in the human malaria parasite Plasmodium falciparum. Mol Cell Proteomics. 2011;10 8:M110.006411. doi:10.1074/mcp.M110.006411.
13. Pandya NJ, Klaassen RV, van der Schors RC, Slotman JA, Houtsmuller A, Smit AB, et al. Group 1 metabotropic glutamate receptors 1 and 5 form a protein complex in mouse hippocampus and cortex. Proteomics. 2016;16 20:2698-705. doi:10.1002/pmic.201500400.
14. Hondius DC, van Nierop P, Li KW, Hoozemans JJ, van der Schors RC, van Haastert ES, et al. Profiling the human hippocampal proteome at all pathologic stages of Alzheimer's disease. Alzheimers Dement. 2016;12 6:654-68. doi:10.1016/j.jalz.2015.11.002.
15. Liu H, Sadygov RG and Yates JR, 3rd. A model for random sampling and estimation of relative protein abundance in shotgun proteomics. Anal Chem. 2004;76 14:4193-201. doi:10.1021/ac0498563.
16. Michalski A, Damoc E, Hauschild JP, Lange O, Wieghaus A, Makarov A, et al. Mass spectrometry-based proteomics using Q Exactive, a high-performance benchtop quadrupole Orbitrap mass spectrometer. Mol Cell Proteomics. 2011;10 9:M111.011015. doi:10.1074/mcp.M111.011015.
17. Gillet LC, Navarro P, Tate S, Rost H, Selevsek N, Reiter L, et al. Targeted data extraction of the MS/MS spectra generated by data-independent acquisition: a new concept for consistent

and accurate proteome analysis. Mol Cell Proteomics. 2012;11 6:O111 016717. doi:10.1074/mcp.O111.016717.
18. Ludwig C, Gillet L, Rosenberger G, Amon S, Collins BC and Aebersold R. Data-independent acquisition-based SWATH-MS for quantitative proteomics: a tutorial. Mol Syst Biol. 2018;14 8:e8126. doi:10.15252/msb.20178126.
19. Rosenberger G, Koh CC, Guo T, Röst HL, Kouvonen P, Collins BC, et al. A repository of assays to quantify 10,000 human proteins by SWATH-MS. Scientific Data. 2014;1:140031. doi:10.1038/sdata.2014.31.
20. Fabre B, Korona D, Mata CI, Parsons HT, Deery MJ, Hertog MLATM, et al. Spectral Libraries for SWATH-MS Assays for Drosophila melanogaster and Solanum lycopersicum. PROTEOMICS. 2017;17 21:1700216. doi:10.1002/pmic.201700216.
21. Blattmann P, Stutz V, Lizzo G, Richard J, Gut P and Aebersold R. Generation of a zebrafish SWATH-MS spectral library to quantify 10,000 proteins. Scientific Data. 2019;6:190011. doi:10.1038/sdata.2019.11.
22. Boddey JA, Moritz RL, Simpson RJ and Cowman AF. Role of the *Plasmodium* export element in trafficking parasite proteins to the infected erythrocyte. Traffic. 2009;10 3:285-99. doi:10.1111/j.1600-0854.2008.00864.x.
23. Straimer J, Gnädig NF, Witkowski B, Amaratunga C, Duru V, Ramadani AP, et al. Drug resistance. K13-propeller mutations confer artemisinin resistance in Plasmodium falciparum clinical isolates. Science (New York, NY). 2015;347 6220:428-31. doi:10.1126/science.1260867.
24. Siddiqui G, Srivastava A, Russell AS and Creek DJ. Multi-omics Based Identification of Specific Biochemical Changes Associated With PfKelch13-Mutant Artemisinin-Resistant Plasmodium falciparum. J Infect Dis. 2017;215 9:1435-44. doi:10.1093/infdis/jix156.
25. Aurrecoechea C, Brestelli J, Brunk BP, Dommer J, Fischer S, Gajria B, et al. PlasmoDB: a functional genomic database for malaria parasites. Nucleic Acids Res. 2009;37 Database issue:D539-43. doi:10.1093/nar/gkn814.
26. Toenhake CG, Fraschka SA, Vijayabaskar MS, Westhead DR, van Heeringen SJ and Bartfai R. Chromatin Accessibility-Based Characterization of the Gene Regulatory Network Underlying *Plasmodium falciparum* Blood-Stage Development. Cell Host Microbe. 2018;23 4:557-69 e9. doi:10.1016/j.chom.2018.03.007.
27. Lopez-Barragan MJ, Lemieux J, Quinones M, Williamson KC, Molina-Cruz A, Cui K, et al. Directional gene expression and antisense transcripts in sexual and asexual stages of *Plasmodium falciparum*. BMC Genomics. 2011;12:587. doi:10.1186/1471-2164-12-587.
28. Zanghi G, Vembar SS, Baumgarten S, Ding S, Guizetti J, Bryant JM, et al. A Specific PfEMP1 Is Expressed in *P. falciparum* Sporozoites and Plays a Role in Hepatocyte Infection. Cell Rep. 2018;22 11:2951-63. doi:10.1016/j.celrep.2018.02.075.
29. Bruderer R, Sondermann J, Tsou CC, Barrantes-Freer A, Stadelmann C, Nesvizhskii AI, et al. New targeted approaches for the quantification of data-independent acquisition mass spectrometry. Proteomics. 2017;17 9 doi:10.1002/pmic.201700021.
30. Tsou CC, Avtonomov D, Larsen B, Tucholska M, Choi H, Gingras AC, et al. DIA-Umpire: comprehensive computational framework for data-independent acquisition proteomics. Nat Methods. 2015;12 3:258-64, 7 p following 64. doi:10.1038/nmeth.3255.
31. Yang T, Yeoh LM, Tutor MV, Dixon MW, McMillan PJ, Xie SC, et al. Decreased K13 Abundance Reduces Hemoglobin Catabolism and Proteotoxic Stress, Underpinning Artemisinin Resistance. Cell reports. 2019;29 9:2917-28. e5.
32. Birnbaum J, Scharf S, Schmidt S, Jonscher E, Hoeijmakers WAM, Flemming S, et al. A Kelch13-defined endocytosis pathway mediates artemisinin resistance in malaria parasites. Science. 2020;367 6473:51-9. doi:10.1126/science.aax4735.
33. Birrell GW, Challis MP, De Paoli A, Anderson D, Devine SM, Heffernan GD, et al. Multi-omic Characterization of the Mode of Action of a Potent New Antimalarial Compound, JPC-3210,

Against *Plasmodium falciparum*. Mol Cell Proteomics. 2020;19 2:308-25. doi:10.1074/mcp.RA119.001797.
34. Pease BN, Huttlin EL, Jedrychowski MP, Talevich E, Harmon J, Dillman T, et al. Global analysis of protein expression and phosphorylation of three stages of *Plasmodium falciparum* intraerythrocytic development. J Proteome Res. 2013;12 9:4028-45. doi:10.1021/pr400394g.
35. Oehring SC, Woodcroft BJ, Moes S, Wetzel J, Dietz O, Pulfer A, et al. Organellar proteomics reveals hundreds of novel nuclear proteins in the malaria parasite *Plasmodium falciparum*. Genome Biol. 2012;13 11:R108. doi:10.1186/gb-2012-13-11-r108.
36. Davies H, Belda H, Broncel M, Ye X, Bisson C, Introini V, et al. An exported kinase family mediates species-specific erythrocyte remodelling and virulence in human malaria. Nat Microbiol. 2020;5 6:848-63. doi:10.1038/s41564-020-0702-4.
37. Giannangelo C, Siddiqui G, De Paoli A, Anderson BM, Edgington-Mitchell LE, Charman SA, et al. System-wide biochemical analysis reveals ozonide antimalarials initially act by disrupting Plasmodium falciparum haemoglobin digestion. bioRxiv. 2020:2020.03.23.003376. doi:10.1101/2020.03.23.003376.
38. Rujimongkon K, Mungthin M, Tummatorn J, Ampawong S, Adisakwattana P, Boonyuen U, et al. Proteomic analysis of Plasmodium falciparum response to isocryptolepine derivative. PLOS ONE. 2019;14 8:e0220871. doi:10.1371/journal.pone.0220871.
39. Dogovski C, Xie SC, Burgio G, Bridgford J, Mok S, McCaw JM, et al. Targeting the cell stress response of Plasmodium falciparum to overcome artemisinin resistance. PLoS biology. 2015;13 4.
40. Searle BC, Pino LK, Egertson JD, Ting YS, Lawrence RT, MacLean BX, et al. Chromatogram libraries improve peptide detection and quantification by data independent acquisition mass spectrometry. Nat Commun. 2018;9 1:5128. doi:10.1038/s41467-018-07454-w.
41. Gnädig NF, Stokes BH, Edwards RL, Kalantarov GF, Heimsch KC, Kuderjavy M, et al. Insights into the intracellular localization, protein associations and artemisinin resistance properties of Plasmodium falciparum K13. PLOS Pathogens. 2020;16 4:e1008482. doi:10.1371/journal.ppat.1008482.
42. Jimenez-Ruiz E, Morlon-Guyot J, Daher W and Meissner M. Vacuolar protein sorting mechanisms in apicomplexan parasites. Mol Biochem Parasitol. 2016;209 1-2:18-25. doi:10.1016/j.molbiopara.2016.01.007.
43. Morse D, Webster W, Kalanon M, Langsley G and McFadden GI. Plasmodium falciparum Rab1A Localizes to Rhoptries in Schizonts. PLoS One. 2016;11 6:e0158174. doi:10.1371/journal.pone.0158174.
44. Cowman AF, Berry D and Baum J. The cellular and molecular basis for malaria parasite invasion of the human red blood cell. J Cell Biol. 2012;198 6:961-71. doi:10.1083/jcb.201206112.
45. Elliott DA, McIntosh MT, Hosgood HD, 3rd, Chen S, Zhang G, Baevova P, et al. Four distinct pathways of hemoglobin uptake in the malaria parasite *Plasmodium falciparum*. Proc Natl Acad Sci U S A. 2008;105 7:2463-8. doi:10.1073/pnas.0711067105.
46. Bunnik EM, Chung DW, Hamilton M, Ponts N, Saraf A, Prudhomme J, et al. Polysome profiling reveals translational control of gene expression in the human malaria parasite *Plasmodium falciparum*. Genome Biol. 2013;14 11:R128. doi:10.1186/gb-2013-14-11-r128.
47. Caro F, Ahyong V, Betegon M and DeRisi JL. Genome-wide regulatory dynamics of translation in the *Plasmodium falciparum* asexual blood stages. Elife. 2014;3 doi:10.7554/eLife.04106.
48. Pavlovic Djuranovic S, Erath J, Andrews RJ, Bayguinov PO, Chung JJ, Chalker DL, et al. *Plasmodium falciparum* translational machinery condones polyadenosine repeats. Elife. 2020;9 doi:10.7554/eLife.57799.
49. Matthews H, Duffy CW and Merrick CJ. Checks and balances? DNA replication and the cell cycle in *Plasmodium*. Parasit Vectors. 2018;11 1:216. doi:10.1186/s13071-018-2800-1.

50. Bozdech Z, Llinas M, Pulliam BL, Wong ED, Zhu J and DeRisi JL. The transcriptome of the intraerythrocytic developmental cycle of Plasmodium falciparum. PLoS Biol. 2003;1 1:E5. doi:10.1371/journal.pbio.0000005.
51. Le Roch KG, Johnson JR, Florens L, Zhou Y, Santrosyan A, Grainger M, et al. Global analysis of transcript and protein levels across the Plasmodium falciparum life cycle. Genome Res. 2004;14 11:2308-18. doi:10.1101/gr.2523904.
52. Cui L, Lindner S and Miao J. Translational regulation during stage transitions in malaria parasites. Ann N Y Acad Sci. 2015;1342:1-9. doi:10.1111/nyas.12573.
53. Adderley JD, John von Freyend S, Jackson SA, Bird MJ, Burns AL, Anar B, et al. Analysis of erythrocyte signalling pathways during *Plasmodium falciparum* infection identifies targets for host-directed antimalarial intervention. Nat Commun. 2020;11 1:4015. doi:10.1038/s41467-020-17829-7.
54. Collins BC, Hunter CL, Liu Y, Schilling B, Rosenberger G, Bader SL, et al. Multi-laboratory assessment of reproducibility, qualitative and quantitative performance of SWATH-mass spectrometry. Nat Commun. 2017;8 1:291. doi:10.1038/s41467-017-00249-5.
55. Toprak UH, Gillet LC, Maiolica A, Navarro P, Leitner A and Aebersold R. Conserved peptide fragmentation as a benchmarking tool for mass spectrometers and a discriminating feature for targeted proteomics. Mol Cell Proteomics. 2014;13 8:2056-71. doi:10.1074/mcp.O113.036475.
56. Zolg DP, Wilhelm M, Schnatbaum K, Zerweck J, Knaute T, Delanghe B, et al. Building ProteomeTools based on a complete synthetic human proteome. Nat Methods. 2017;14 3:259-62. doi:10.1038/nmeth.4153.
57. Barkovits K, Pacharra S, Pfeiffer K, Steinbach S, Eisenacher M, Marcus K, et al. Reproducibility, Specificity and Accuracy of Relative Quantification Using Spectral Library-based Data-independent Acquisition. Mol Cell Proteomics. 2020;19 1:181-97. doi:10.1074/mcp.RA119.001714.
58. Trager W and Jensen JB. Human malaria parasites in continuous culture. Science. 1976;193 4254:673-5.
59. Creek DJ, Chua HH, Cobbold SA, Nijagal B, MacRae JI, Dickerman BK, et al. Metabolomics-Based Screening of the Malaria Box Reveals both Novel and Established Mechanisms of Action. Antimicrob Agents Chemother. 2016;60 11:6650-63. doi:10.1128/AAC.01226-16.
60. Tjhin ET, Spry C, Sewell AL, Hoegl A, Barnard L, Sexton AE, et al. Mutations in the pantothenate kinase of Plasmodium falciparum confer diverse sensitivity profiles to antiplasmodial pantothenate analogues. PLOS Pathogens. 2018;14 4:e1006918. doi:10.1371/journal.ppat.1006918.
61. Cobbold SA, Vaughan AM, Lewis IA, Painter HJ, Camargo N, Perlman DH, et al. Kinetic flux profiling elucidates two independent acetyl-CoA biosynthetic pathways in Plasmodium falciparum. The Journal of biological chemistry. 2013;288 51:36338-50. doi:10.1074/jbc.M113.503557.
62. Rappsilber J, Ishihama Y and Mann M. Stop and Go Extraction Tips for Matrix-Assisted Laser Desorption/Ionization, Nanoelectrospray, and LC/MS Sample Pretreatment in Proteomics. Analytical Chemistry. 2003;75 3:663-70. doi:10.1021/ac026117i.
63. Escher C, Reiter L, MacLean B, Ossola R, Herzog F, Chilton J, et al. Using iRT, a normalized retention time for more targeted measurement of peptides. Proteomics. 2012;12 8:1111-21. doi:10.1002/pmic.201100463.
64. Chong J, Soufan O, Li C, Caraus I, Li S, Bourque G, et al. MetaboAnalyst 4.0: towards more transparent and integrative metabolomics analysis. Nucleic Acids Research. 2018;46 W1:W486-W94. doi:10.1093/nar/gky310.
65. Supek F, Bosnjak M, Skunca N and Smuc T. REVIGO summarizes and visualizes long lists of gene ontology terms. PLoS One. 2011;6 7:e21800. doi:10.1371/journal.pone.0021800.

66. Shannon P, Markiel A, Ozier O, Baliga NS, Wang JT, Ramage D, et al. Cytoscape: a software environment for integrated models of biomolecular interaction networks. Genome Res. 2003;13 11:2498-504. doi:10.1101/gr.1239303.
67. Perez-Riverol Y, Csordas A, Bai J, Bernal-Llinares M, Hewapathirana S, Kundu DJ, et al. The PRIDE database and related tools and resources in 2019: improving support for quantification data. Nucleic Acids Res. 2019;47 D1:D442-D50. doi:10.1093/nar/gky1106.
68. Siddiqui G; De Paoli A; MacRaild CA; Sexton AE; Boulet C ; Shah AD; Batty MB; Schittenhelm RB; Carvalho TG; Creek DJ: Supporting data for "A new mass spectral library for high-coverage and reproducible analysis of the Plasmodium falciparum-infected red blood cell proteome." GigaScience Database. 2022. http://dx.doi.org/10.5524/100962

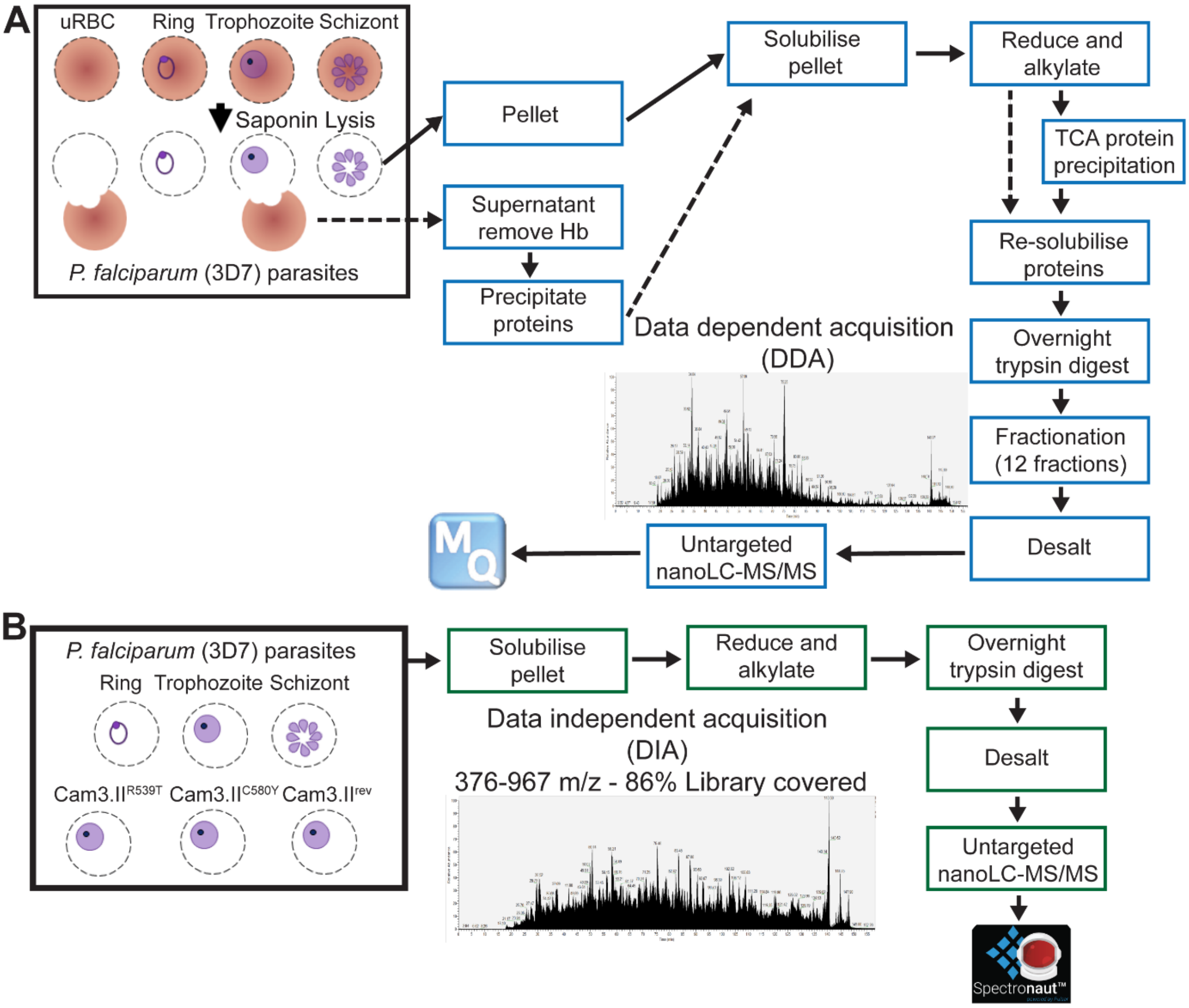

Figure 1. Flow chart for creation and application of the spectral library for *Plasmodium falciparum* infected red blood cells. (A) The spectral library (blue boxes) was built from uninfected red blood cells (uRBC), ring-, trophozoite- and schizont-stage (*P. falciparum* 3D7) parasites, where both the saponin pellets (solid arrows) and supernatants (dashed arrows) were combined (3-5 mg of proteins), trypsinised, fractionated extensively and data analysed using Maxquant. (B) The spectral library was then used to analyse samples (green boxes) from *P. falciparum* 3D7 parasites (ring-, trophozoite- and schizont-stage) and artemisinin resistant (Cam3.II^{R539T}, Cam3.II^{C580Y}) and sensitive (Cam3.II^{rev}) parasites (saponin pellets only, 500 µg of proteins) over a window of 376-967 *m/z* covering 86% of the spectral library generated. The data was analysed using Spectronaut^{TM}.

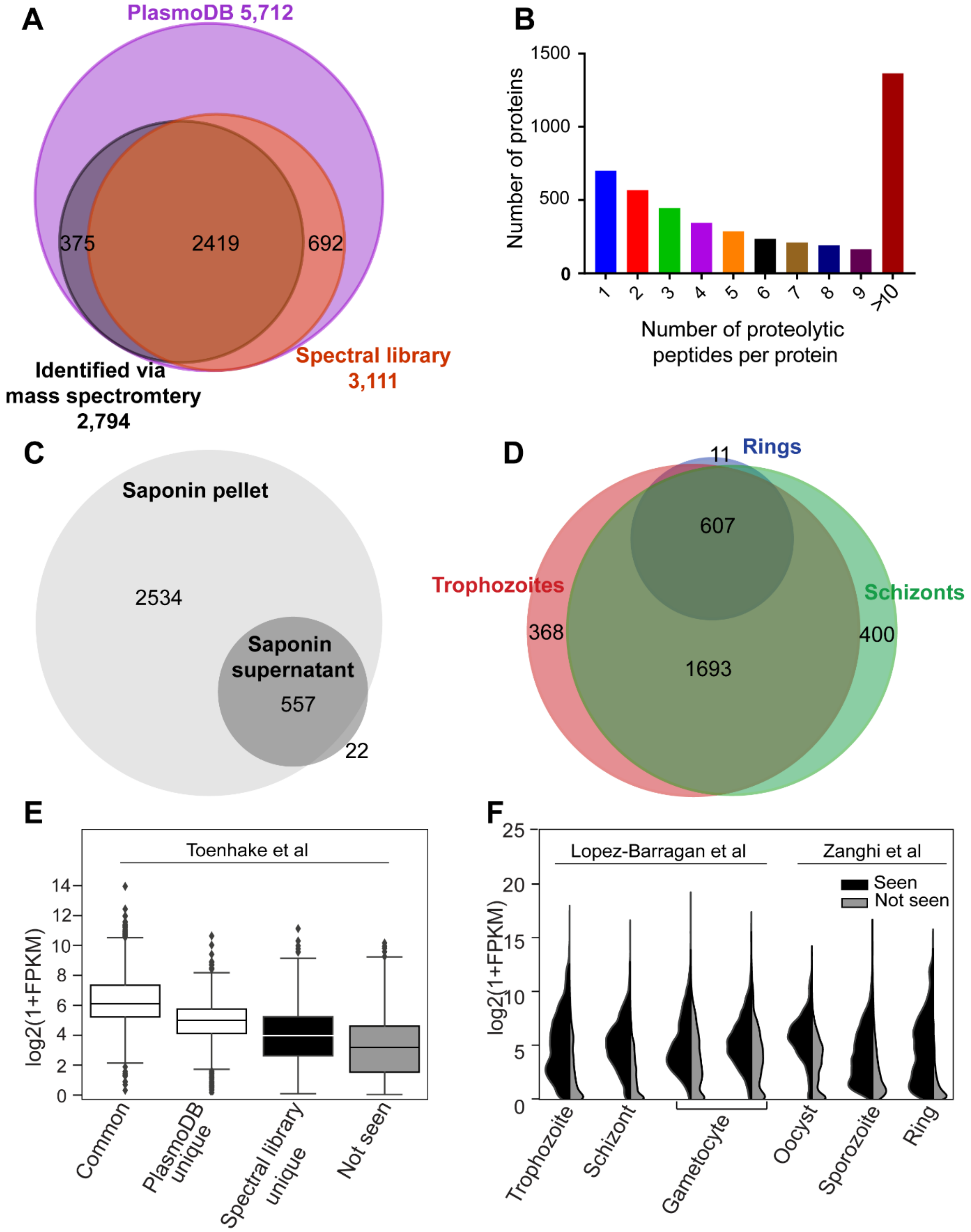

Figure 2. Characterisation of *Plasmodium falciparum* asexual red blood cell stage spectral library. (A) Venn diagram describing overlap of identified *P. falciparum* proteins from the generated spectral library (orange) with *P. falciparum* protein-encoding genes in PlasmoDB (purple), and PlasmoDB subset annotated with protein level evidence from mass spectrometry (black) (B) The number of proteotypic peptides per protein in the *P. falciparum* spectral library (C) Venn diagram of identified *P.*

falciparum proteins for spectral library generation from saponin soluble and insoluble fractions, and (D) of ring-, trophozoite- and schizont-stage parasites. (E) Box plot of FPKM distribution of transcript expression level from Toenhake *et al.* [26] dataset for genes common to spectral library and PlasmoDB (common), unique to PlasmoDB and spectral library, and not identified (not seen) with logarithmic values of FPKM on the vertical axis. (F) Violin plots displaying the expression intensity distribution of the genes from Lopez-Barragan *et al.* [27] and Zanghi *et al.* [28] datasets with products identified (seen) and not identified (not seen) in the *P. falciparum* spectral library across the distinct life cycle stages.

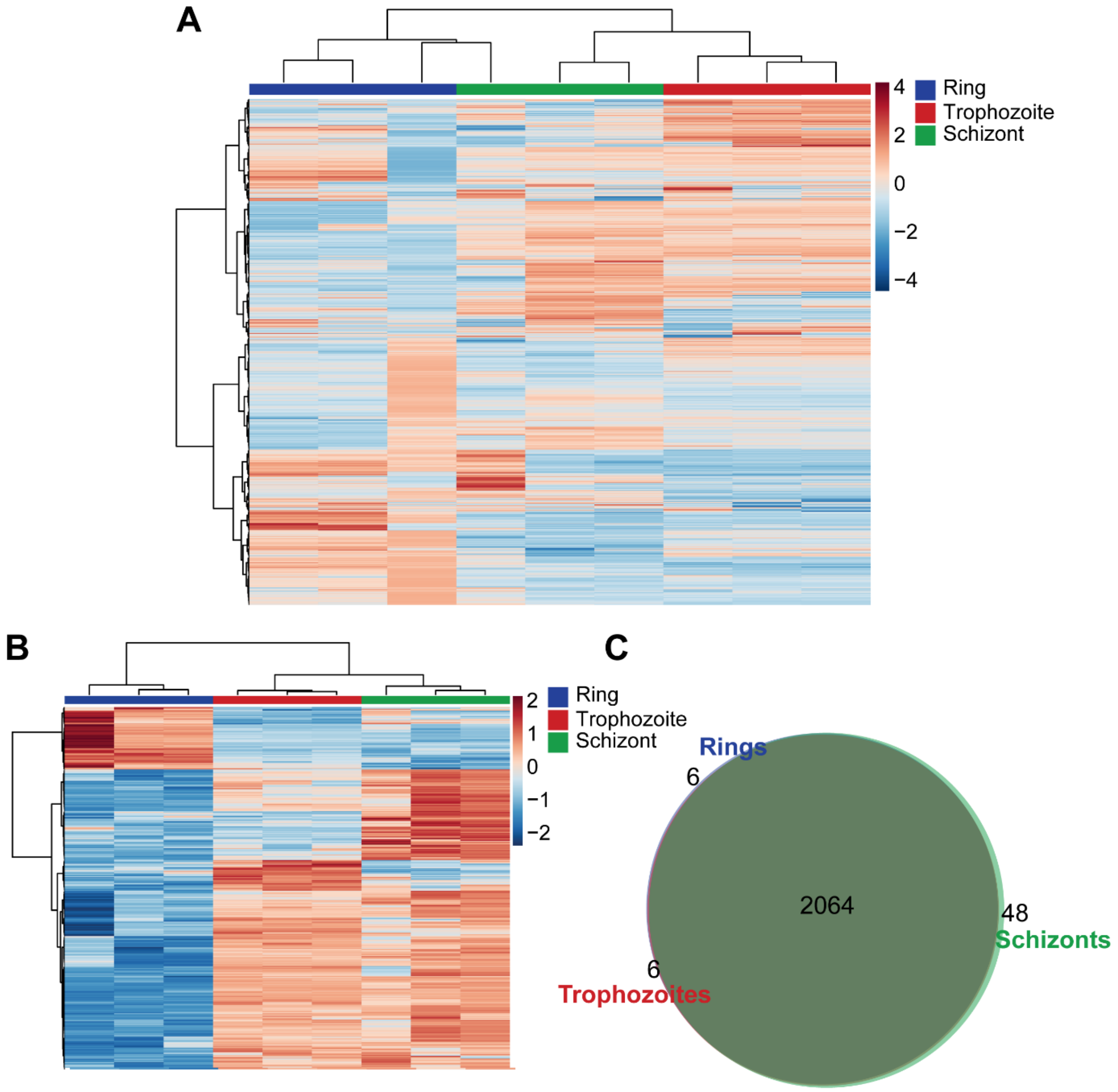

Figure 3. Hierarchical clustering of *P. falciparum* proteins of ring- (blue), trophozoite- (red), and schizont-stage parasites (green). Vertical clustering displays similarities between sample groups, while horizontal clusters reveal the relative abundances of all identified proteins (2064) (A) and the 400 (B) most significantly different proteins from DIA analysis. (C) Venn diagram of identified *P. falciparum* proteins in ring-, trophozoite- and schizont-stage parasites using the DIA-MS. Data shown is from three independent biological replicates. The colour scale bar represents $\log_2$ (mean-centered and divided by the standard deviation of each variable) intensity values.

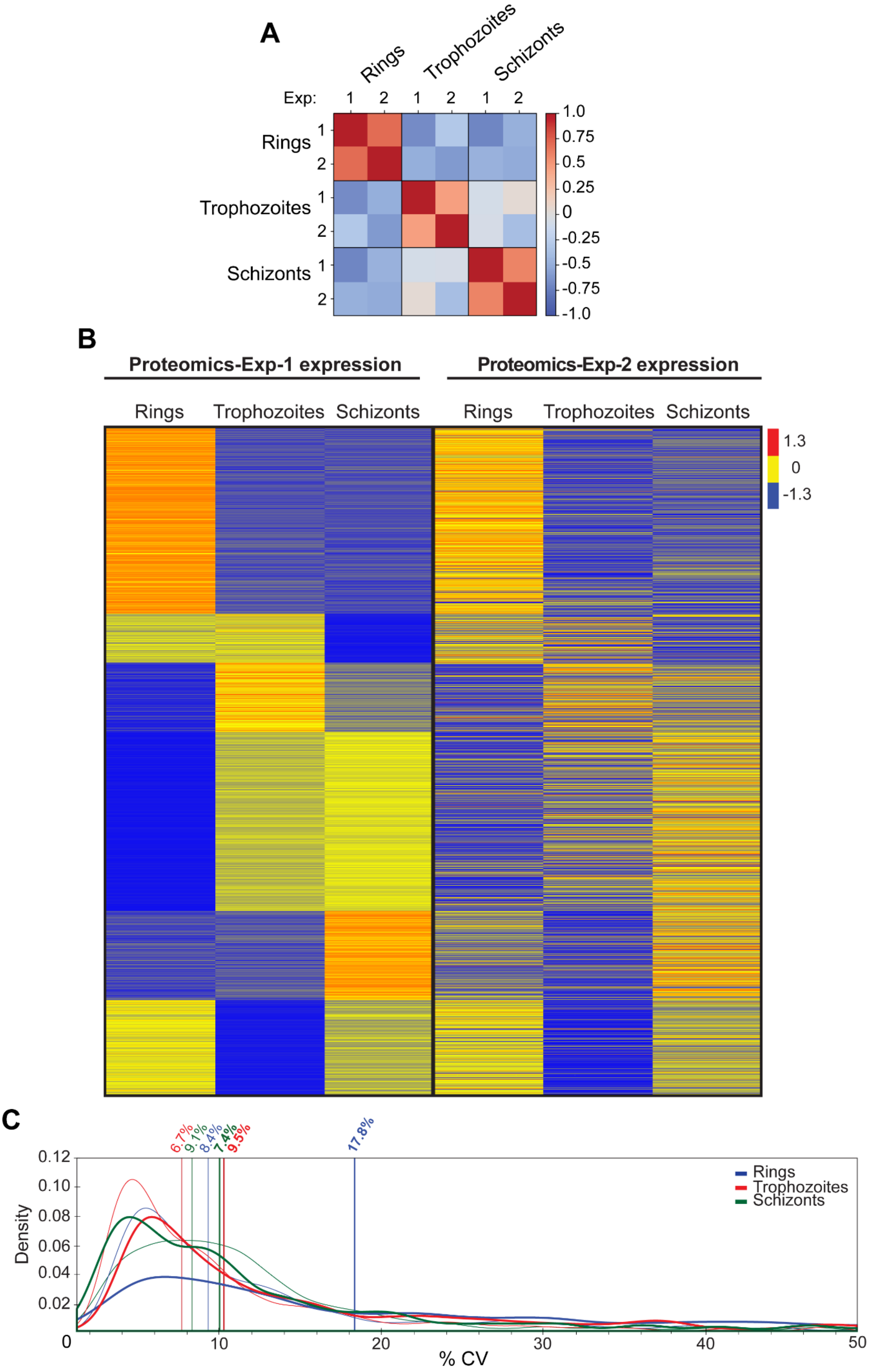
A
Rings
Trophozoites
Schizonts
Exp:
1
2
1.0
0.75
0.5
0.25
0
-0.25
-0.5
-0.75
-1.0
B
Proteomics-Exp-1 expression
Proteomics-Exp-2 expression
Rings
Trophozoites
Schizonts
1.3
0
-1.3
C
6.7%
9.1%
8.4%
7.4%
9.5%
17.8%
Density
0.12
0.10
0.08
0.06
0.04
0.02
0
10
20
30
40
50
% CV
Rings
Trophozoites
Schizonts

Figure 4. Analysing stage specific DIA-MS data using the *P. falciparum* spectral library. (A) Pearson correlation of average protein intensities in each specific stage (rings-, trophozoites- and schizont-stage parasites) identified in two experiments 1 and 2. (B) Heatmap of the *P. falciparum* average protein expression patterns of 1,990 common proteins from ring- to trophozoite- to schizont-stage parasites in experiment 1 and experiment 2. The left portion of heatmap represents proteomics data (experiment 1), and the right represents an independent proteomics data set (experiment 2). The colour code is as follows: red indicates up-regulated proteins; blue indicates down-regulated proteins; yellow indicates unchanged proteins. (C) Coefficient of Variation (%CV) of protein intensities in experiment 1 (n = 3) and experiment 2 (n = 2-3) for each of the distinct stages. Bold lines are experiment 2. Vertical lines indicate median %CV for each stage in each experiment.

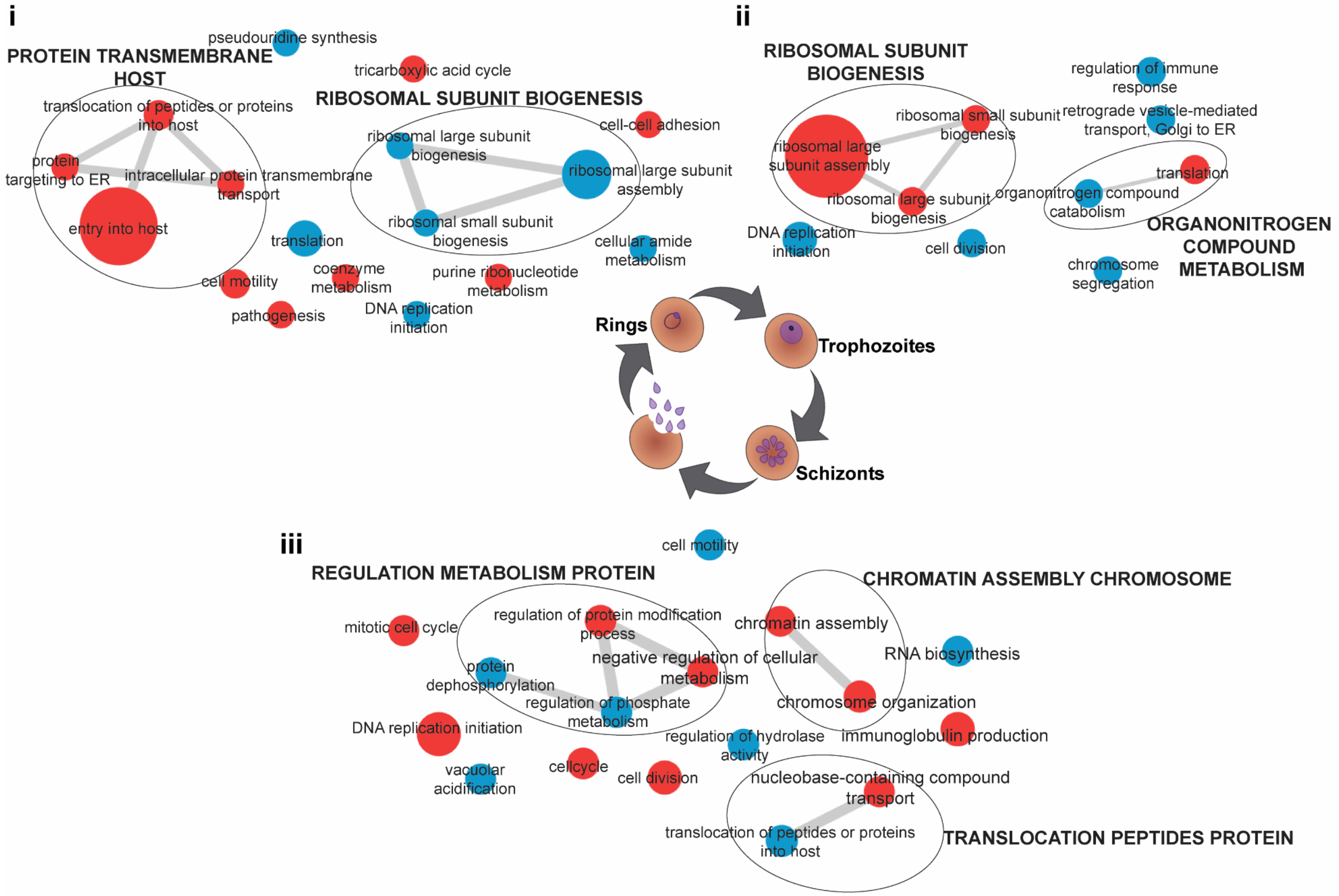
i
PROTEIN TRANSMEMBRANE HOST
pseudouridine synthesis
tricarboxylic acid cycle
RIBOSOMAL SUBUNIT BIOGENESIS
translocation of peptides or proteins into host
protein targeting to ER
intracellular protein transmembrane transport
entry into host
cell motility
pathogenesis
translation
coenzyme metabolism
ribosomal large subunit biogenesis
ribosomal large subunit assembly
ribosomal small subunit biogenesis
cell-cell adhesion
cellular amide metabolism
purine ribonucleotide metabolism
DNA replication initiation
ii
RIBOSOMAL SUBUNIT BIOGENESIS
ribosomal large subunit assembly
ribosomal small subunit biogenesis
ribosomal large subunit biogenesis
DNA replication initiation
cell division
regulation of immune response
retrograde vesicle-mediated transport, Golgi to ER
translation
organonitrogen compound catabolism
ORGANONITROGEN COMPOUND METABOLISM
chromosome segregation
Rings
Trophozoites
Schizonts
iii
cell motility
REGULATION METABOLISM PROTEIN
mitotic cell cycle
regulation of protein modification process
protein dephosphorylation
negative regulation of cellular metabolism
regulation of phosphate metabolism
DNA replication initiation
vacuolar acidification
cellcycle
cell division
regulation of hydrolase activity
CHROMATIN ASSEMBLY CHROMOSOME
chromatin assembly
RNA biosynthesis
chromosome organization
immunoglobulin production
nucleobase-containing compound transport
translocation of peptides or proteins into host
TRANSLOCATION PEPTIDES PROTEIN

Figure 5. Gene Ontology network analysis of processes enriched in the spectral library for each stage of asexual development. Gene Ontology (GO) terms significantly overrepresented in the transition between the three distinct stages of the asexual development (**i**; schizont- to ring-stage parasites, **ii**; ring- to trophozoite-stage parasites, **iii**; trophozoite- to schizont-stage parasites transition) were obtained using in-house methods and imported into REVIGO for network compilation. Visualisation of network enrichment was performed using Cytoscape (v3.8.2). Each node is sized by increasing GO term uniqueness and coloured according whether the process is up (red) or down (blue) by comparison to the subsequent developmental stage. Grey bars connecting each node represent gene overlap between terms.

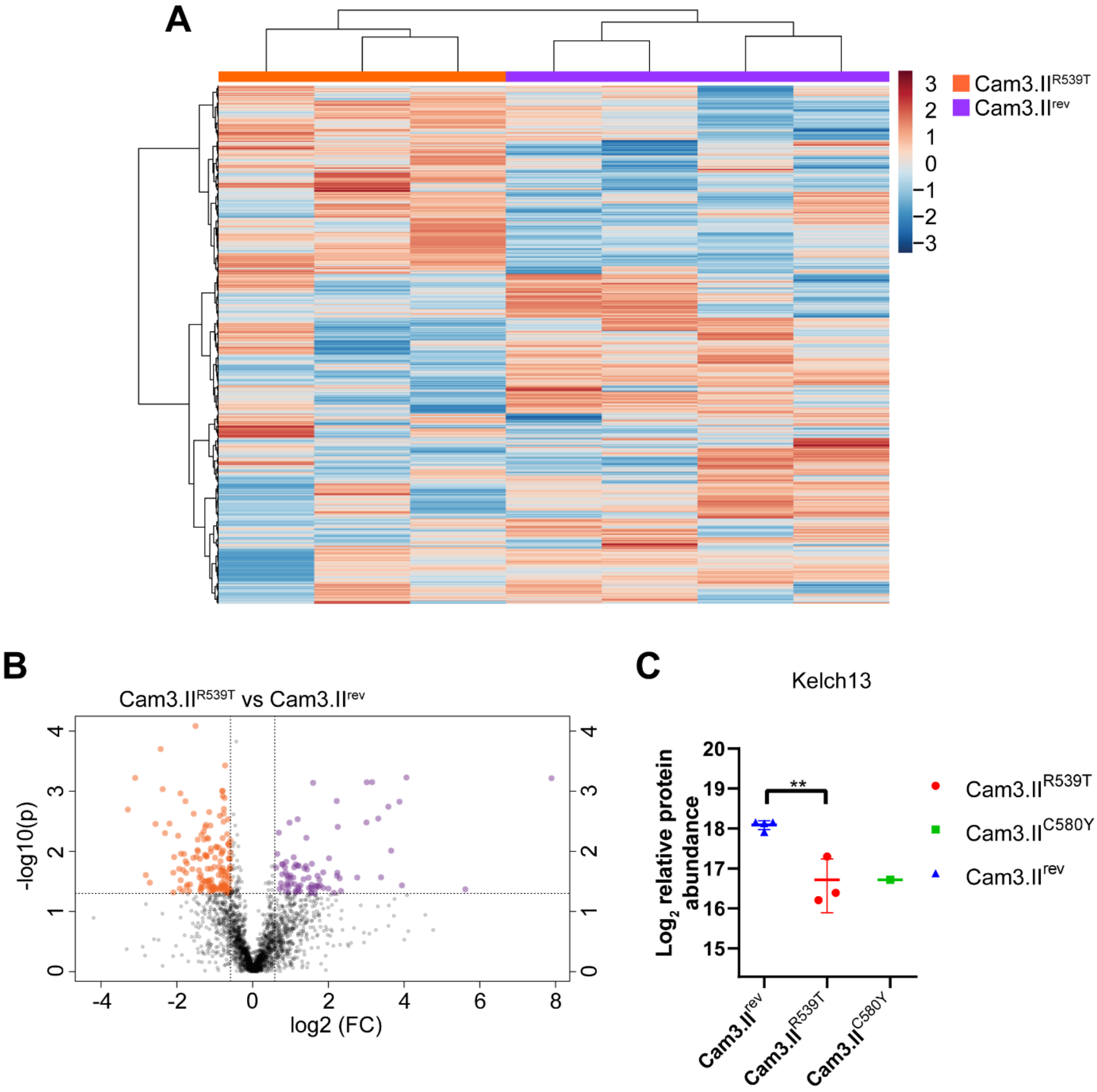

Figure 6. DIA-MS analysis of artemisinin resistant (Cam3.II^{R539T}) and sensitive (Cam3.II^{rev}) parasites using the *P. falciparum* spectral library. (A) Hierarchical clustering of the *P. falciparum* proteins of Cam3.II^{R539T} (orange, n=3) and Cam3.II^{rev} (purple, n=4). Vertical clustering displays similarities between sample groups, while horizontal clusters reveal the relative abundances of identified *P. falciparum* proteins (2,317). The colour scale bar represents $\log_2$ (mean-centered and divided by the standard deviation of each variable) intensity values. (B) Volcano plot of differential protein abundance between Cam3.II^{R539T} and Cam3.II^{rev} parasites. Proteins above the significance threshold (p-value < 0.05) and fold change ≥ 1.5 are shown as orange (up-regulated in Cam3.II^{R539T}) and purple (up-regulated in Cam3.II^{rev}) dots. Data shown from at least three independents biological replicates. (C) Log_2 relative abundance (mean ± standard deviation) of PF3D7_1343700 (Kelch13) in Cam3.II^{rev}, Cam3.II^{R539T}, and Cam3.II^{C580Y}. ** p-value = 0.001.

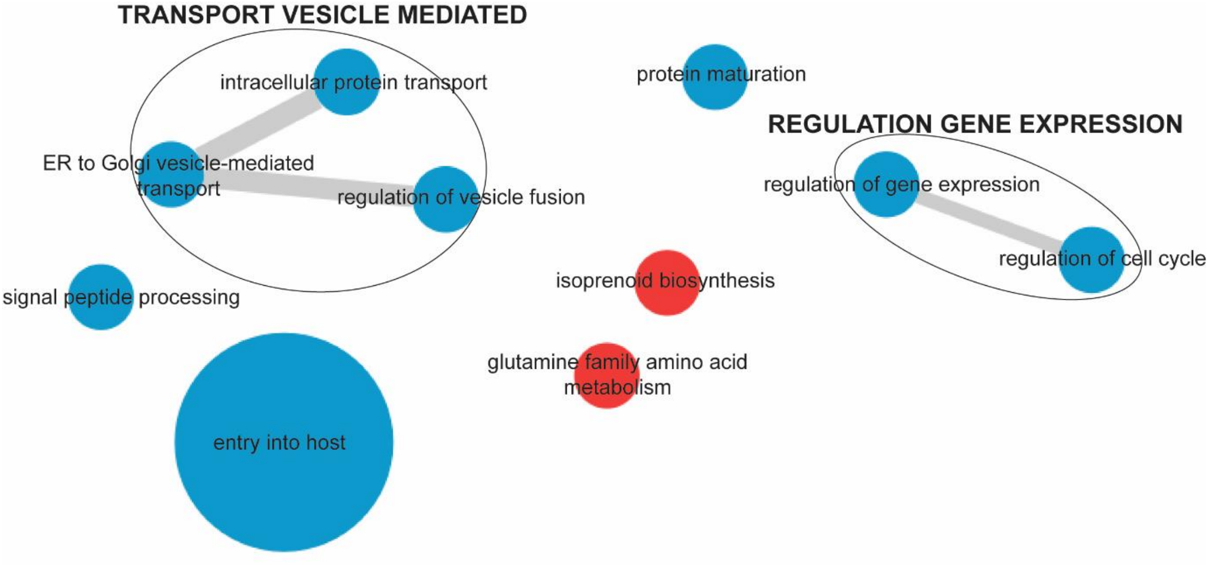

Figure 7. Comparative enrichment analysis of trophozoite-stage artemisinin resistant (Cam3.II^{R539T}) and sensitive (Cam3.II^{rev}) parasites. Pathway enrichment networks were prepared from significantly overrepresented GO terms obtained by in-house statistical methods. Nodes are sized by increasing GO term uniqueness and coloured according to whether a process is up (red) or down (blue) in resistant versus sensitive parasites.

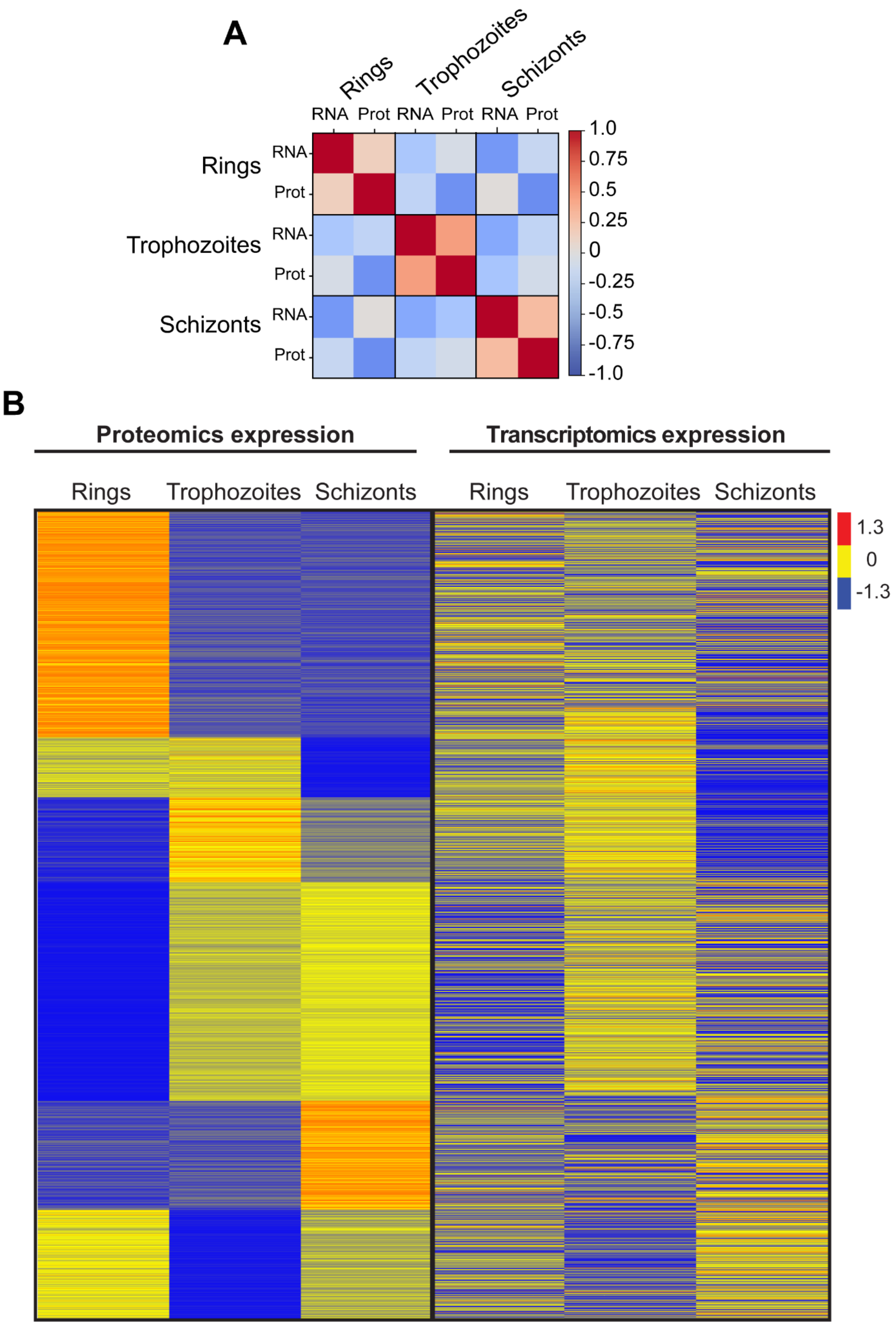

Figure 8. Heatmap of the *P. falciparum* gene and protein expression patterns from ring- to

trophozoite- to schizont-stage parasites. (A) Pearson correlation of average protein intensities (1990 common proteins identified in Experiment 1 and 2) in each specific stage (rings-, trophozoites- and schizont-stage parasites) to RNA transcript abundance from Toenhake *et al.* [26]. (B) The left portion of heatmap represents proteomics data (DIA) of 1990 common proteins from ring- to trophozoite- to schizont-stage parasites in experiment 1 and experiment 2. While, the right represents transcriptomics data from Toenhake *et al.* [26]. The colour code is as follows: red indicates up-regulated proteins or transcripts; blue indicates down-regulated proteins or transcripts; yellow indicates unchanged proteins or transcripts.

Figure 1

Click here to access/download;Figure;Figure_1 updated.tif

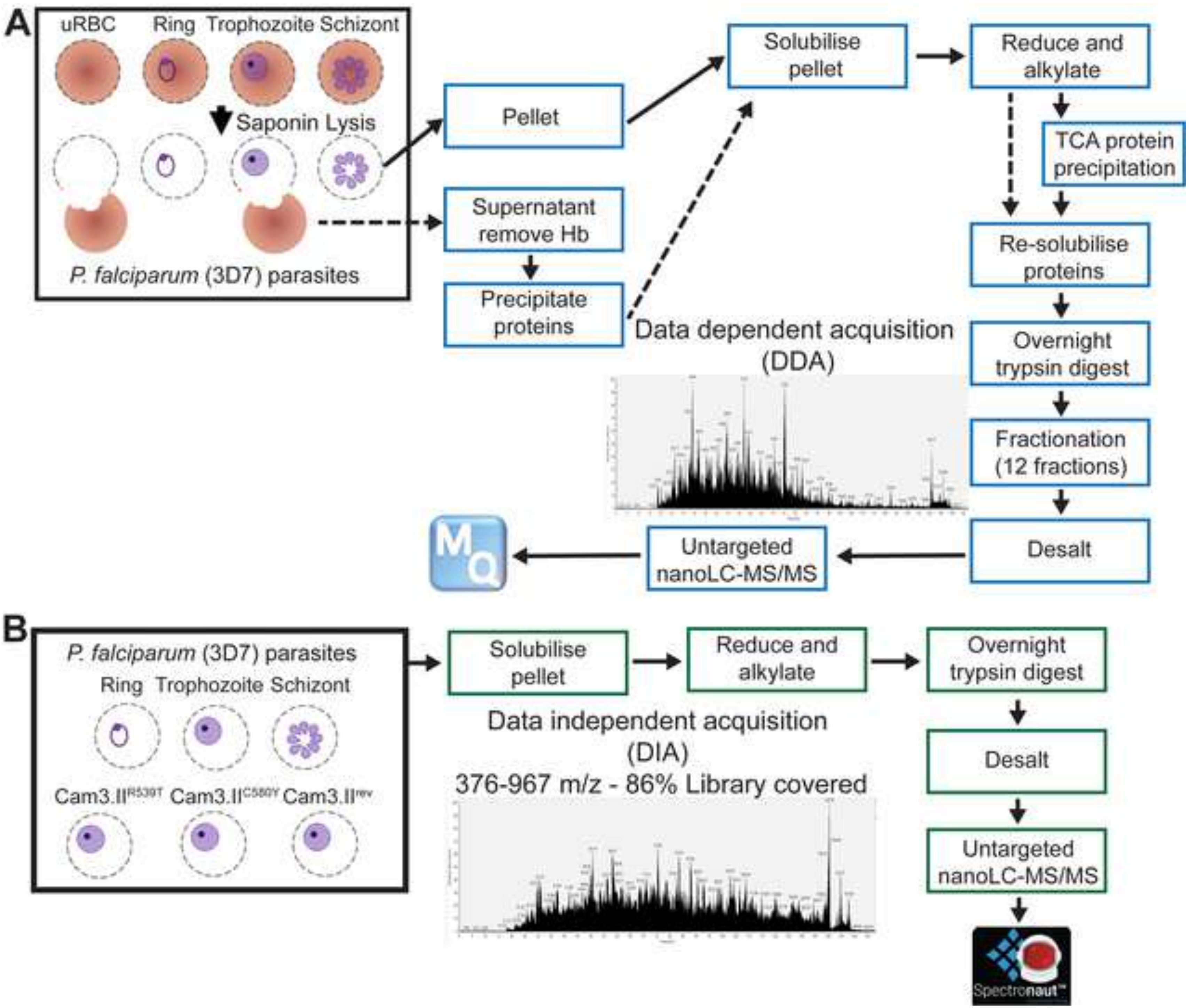

Figure 2

Click here to access/download;Figure;Figure_2-v1.tif

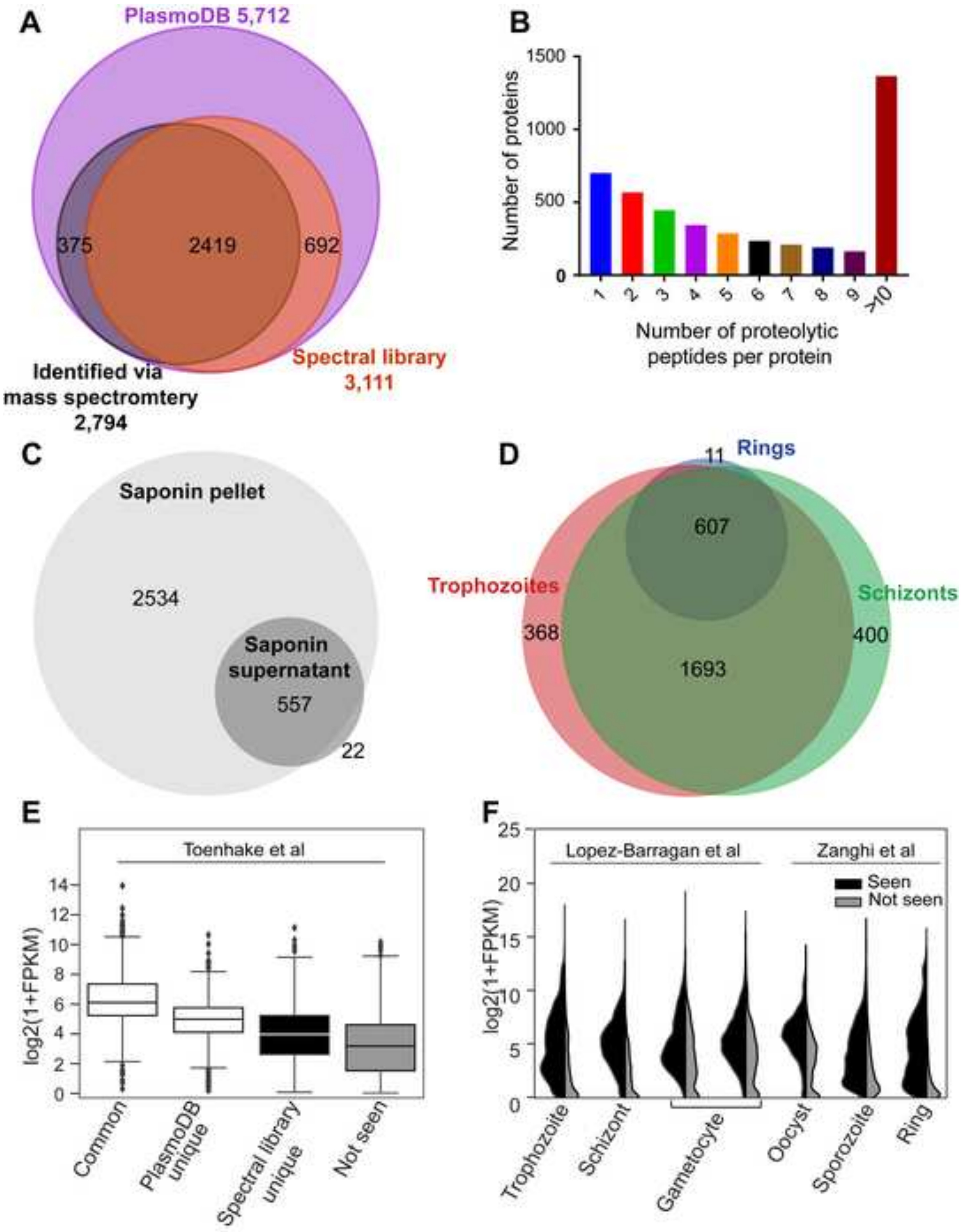

Figure 3

Click here to access/download;Figure;Figure_3-v1.tif

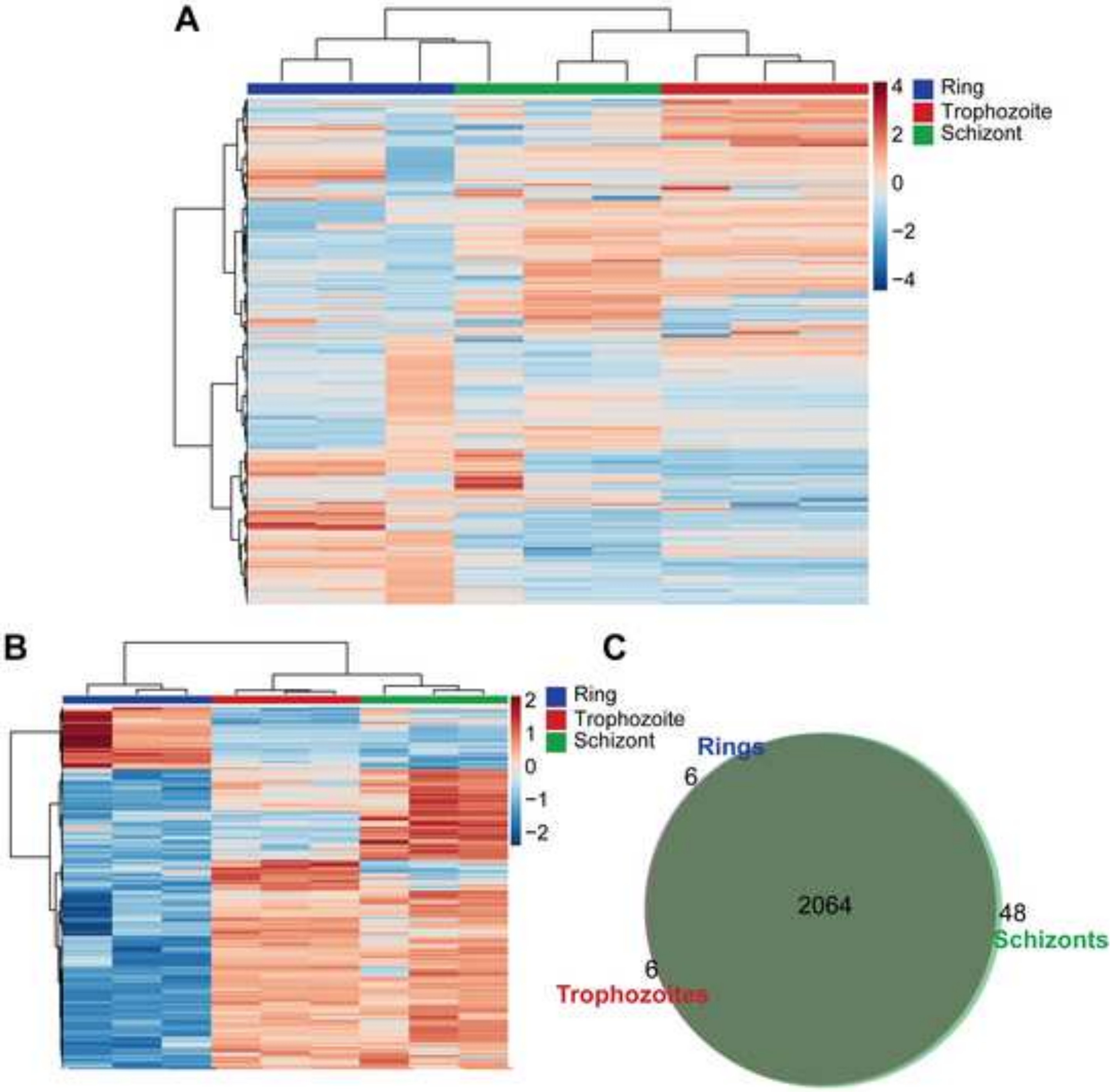

Click here to access/download;Figure;Figure_4.tif

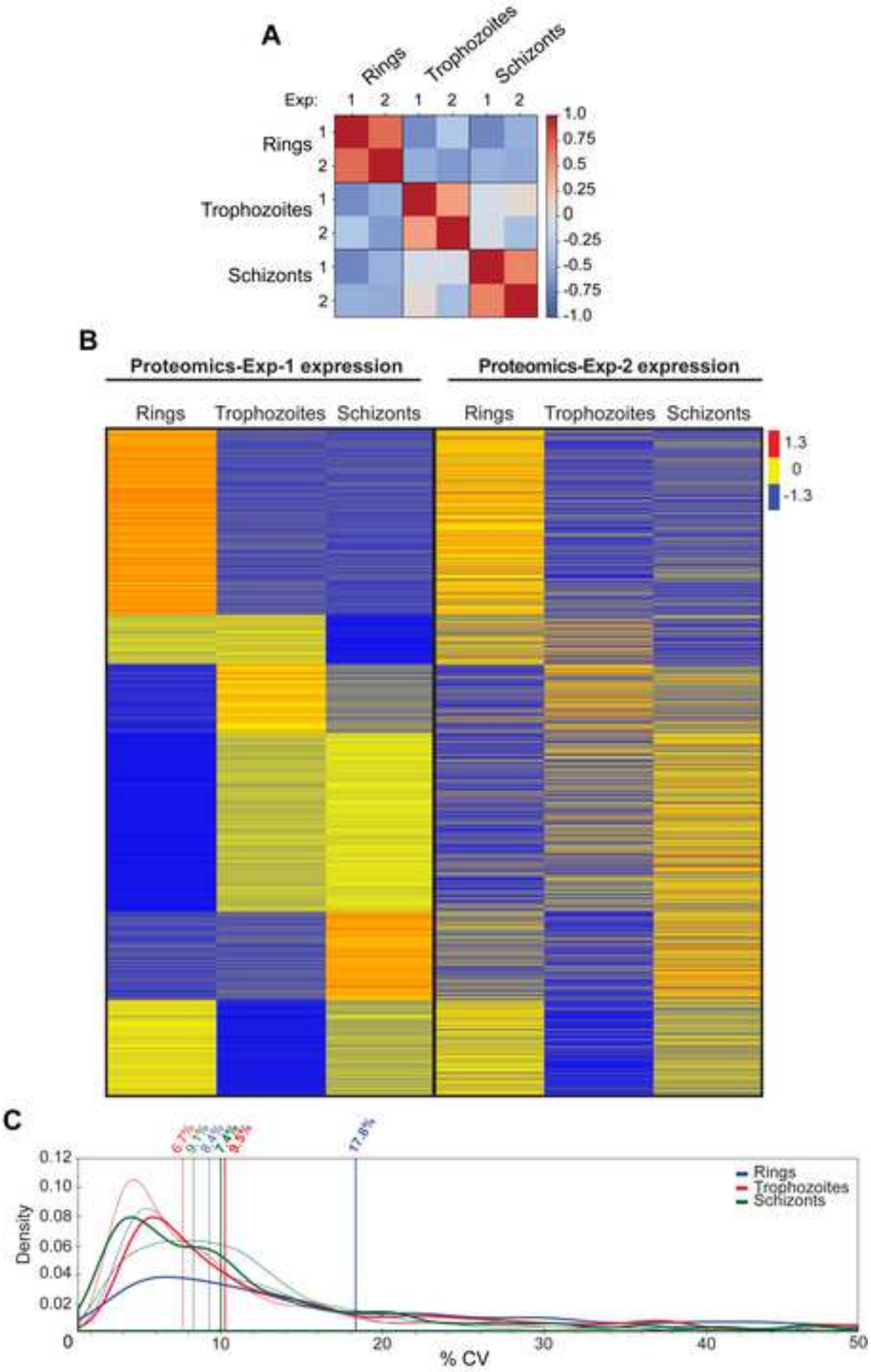

Figure 5 Click here to access/download;Figure;Figure_5.tif

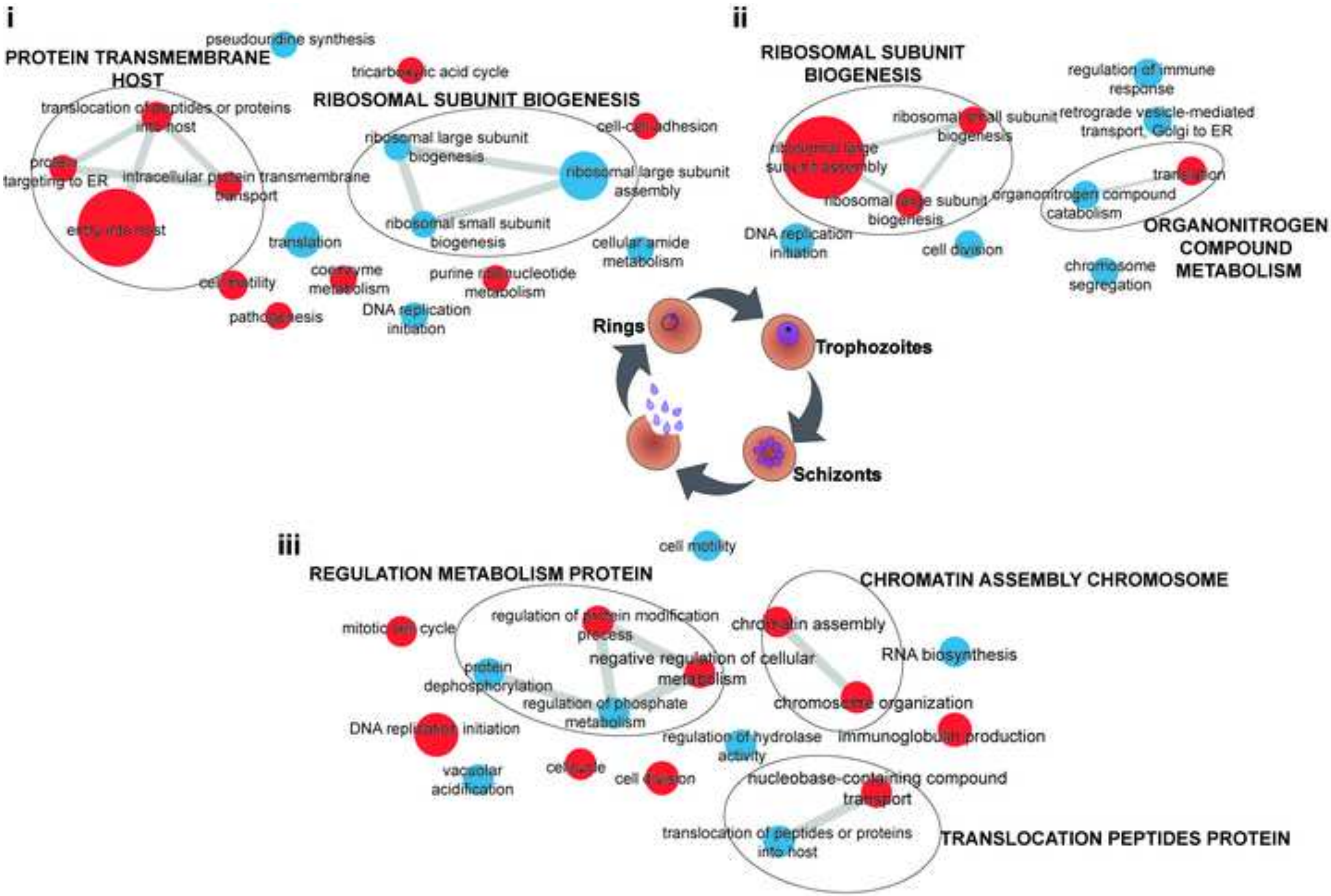

Figure 6

Click here to access/download;Figure;Figure_6.tif

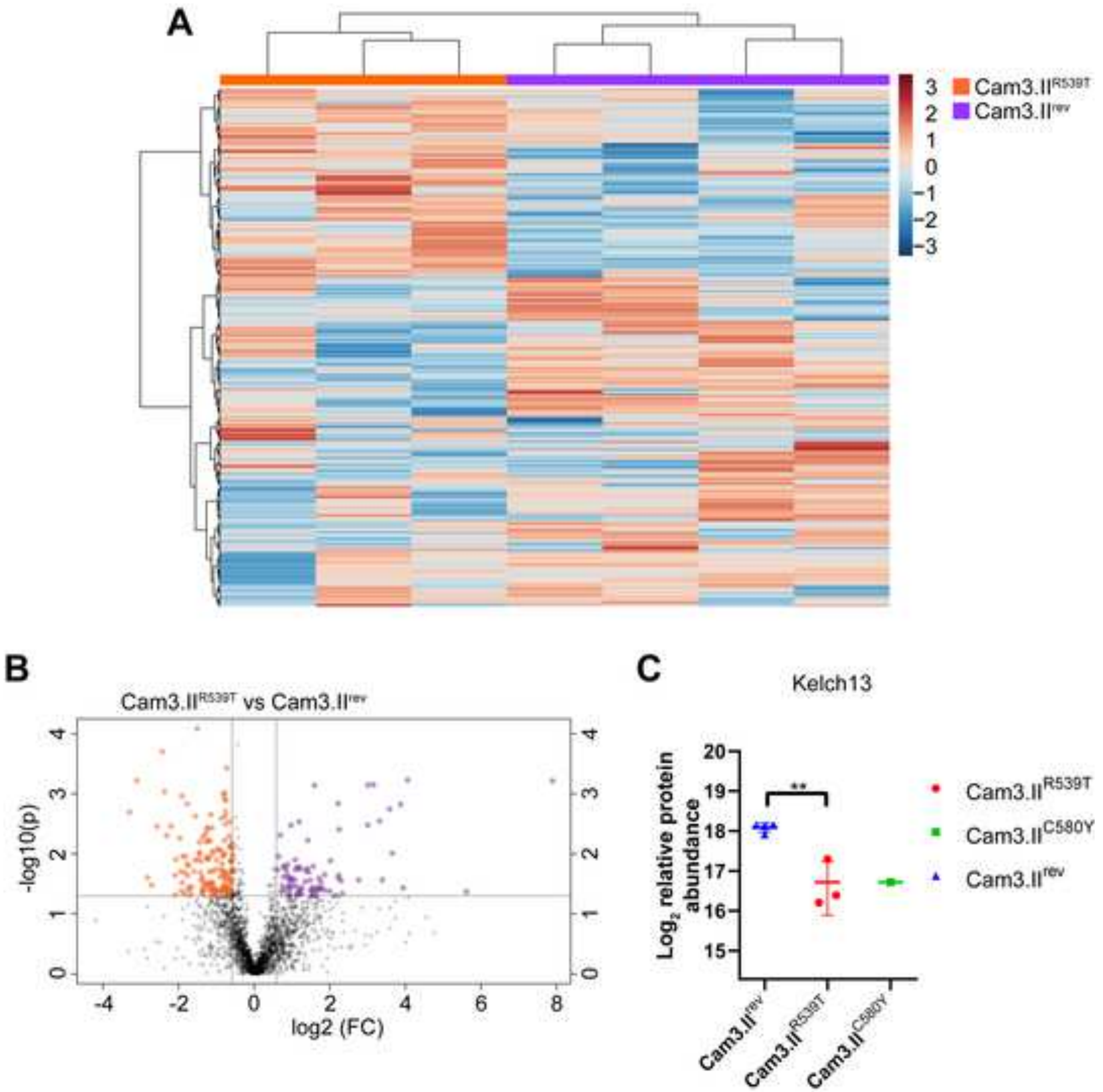

Figure 7

Click here to access/download;Figure;Figure_7.tif

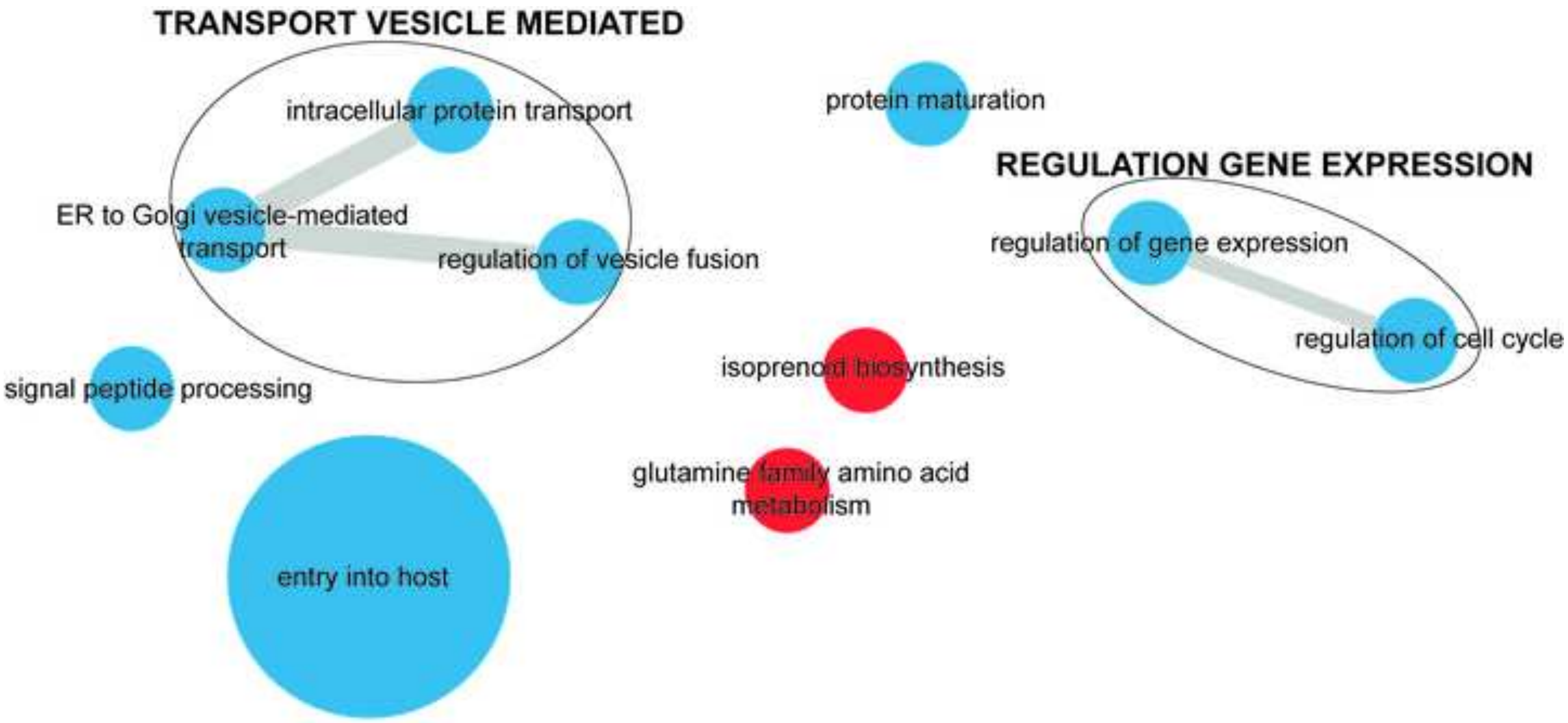

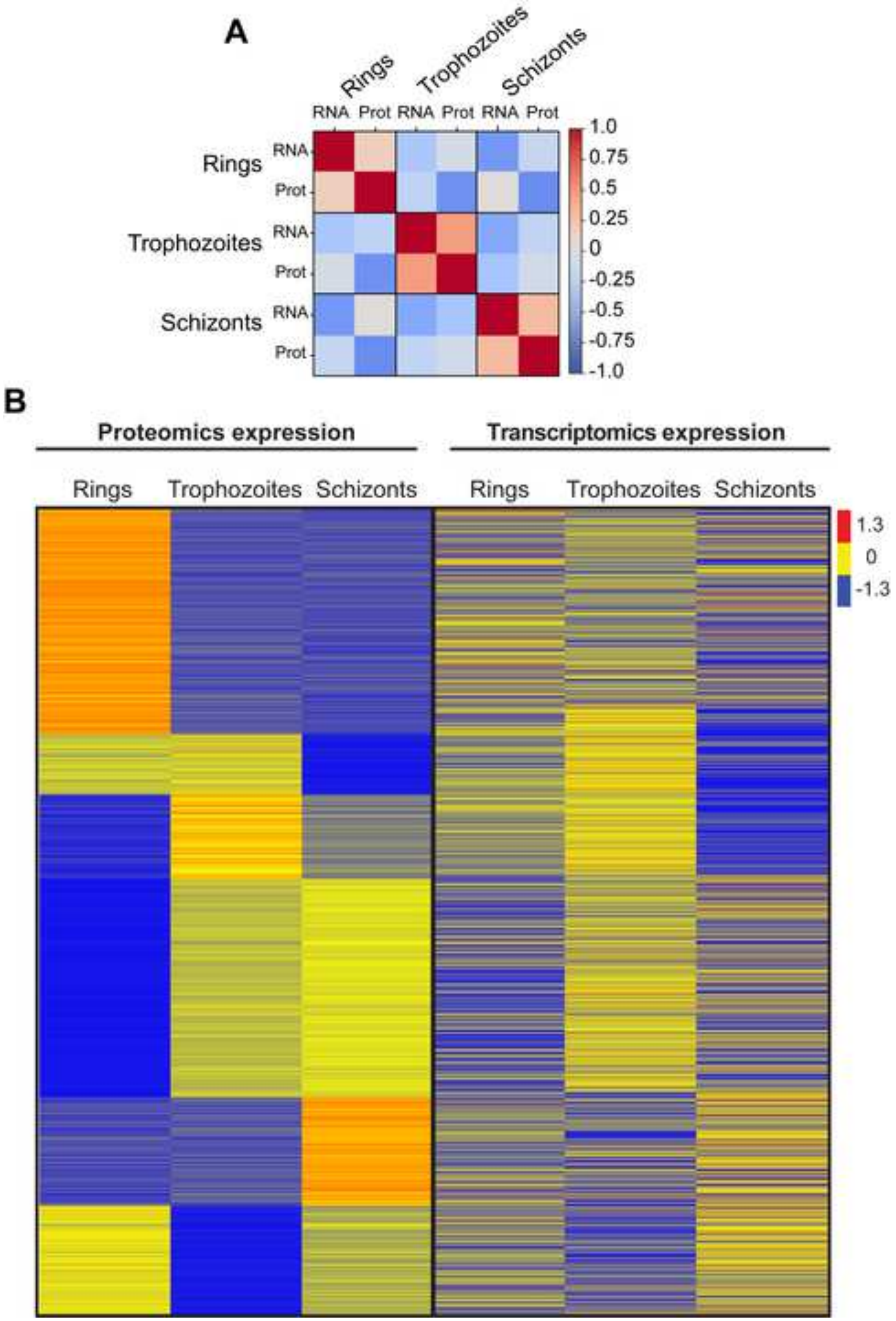
A
Rings
Trophozoites
Schizonts
RNA
Prot
RNA
Prot
RNA
Prot
1.0
0.75
0.5
0.25
0
-0.25
-0.5
-0.75
-1.0
B
Proteomics expression
Transcriptomics expression
Rings
Trophozoites
Schizonts
Rings
Trophozoites
Schizonts
1.3
0
-1.3

Supporting Figure 1

Click here to access/download;Figure;Supp_Figure_1_venndigram_human_so

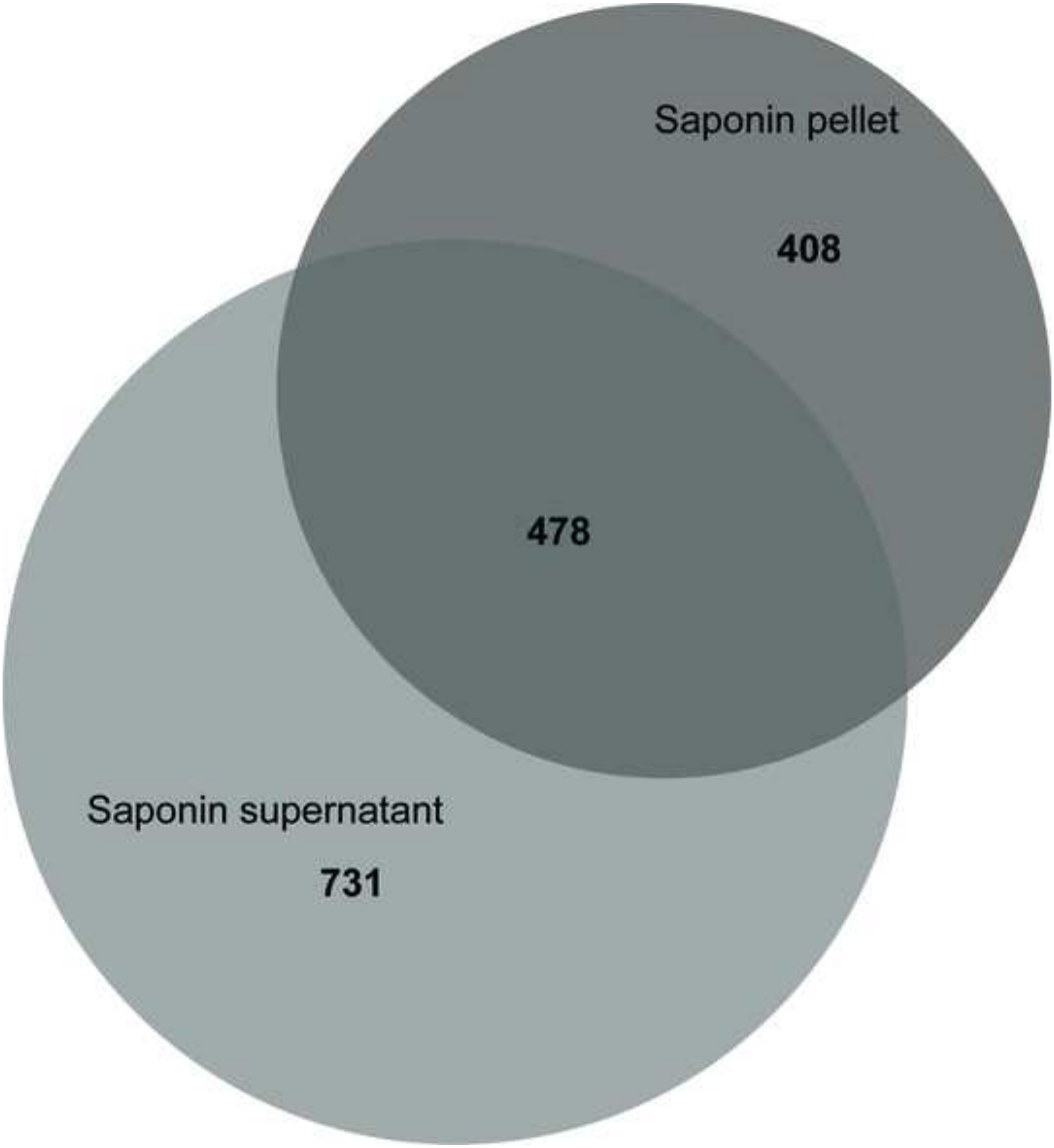

Supporting Figure 2

Click here to access/download;Figure;Supp_Figure_2_heatmap_venndigram_DIA_exp_1_exp_2-

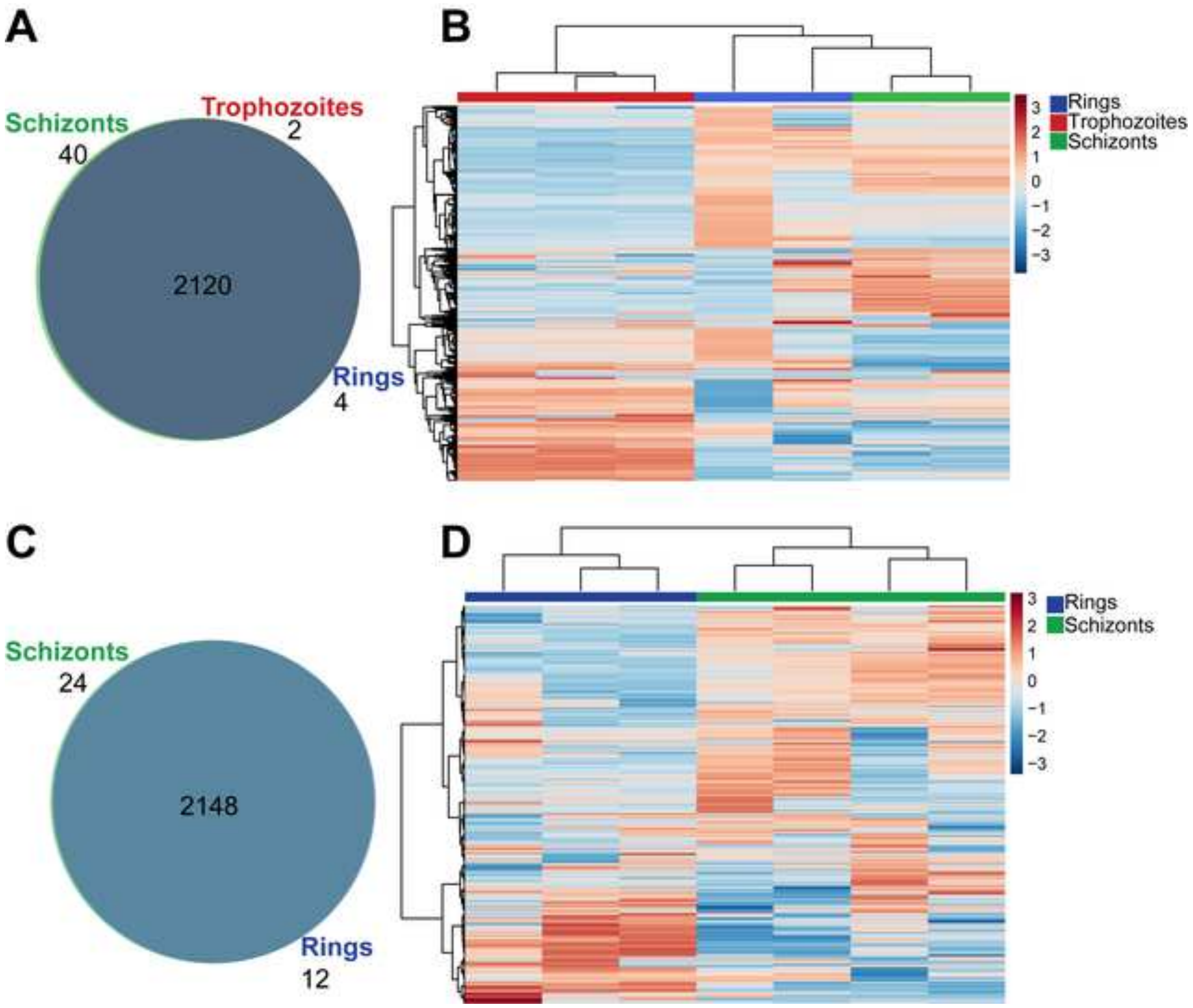

Supporting Figure 3

Click here to access/download;Figure;Supp_Figure_3.tif

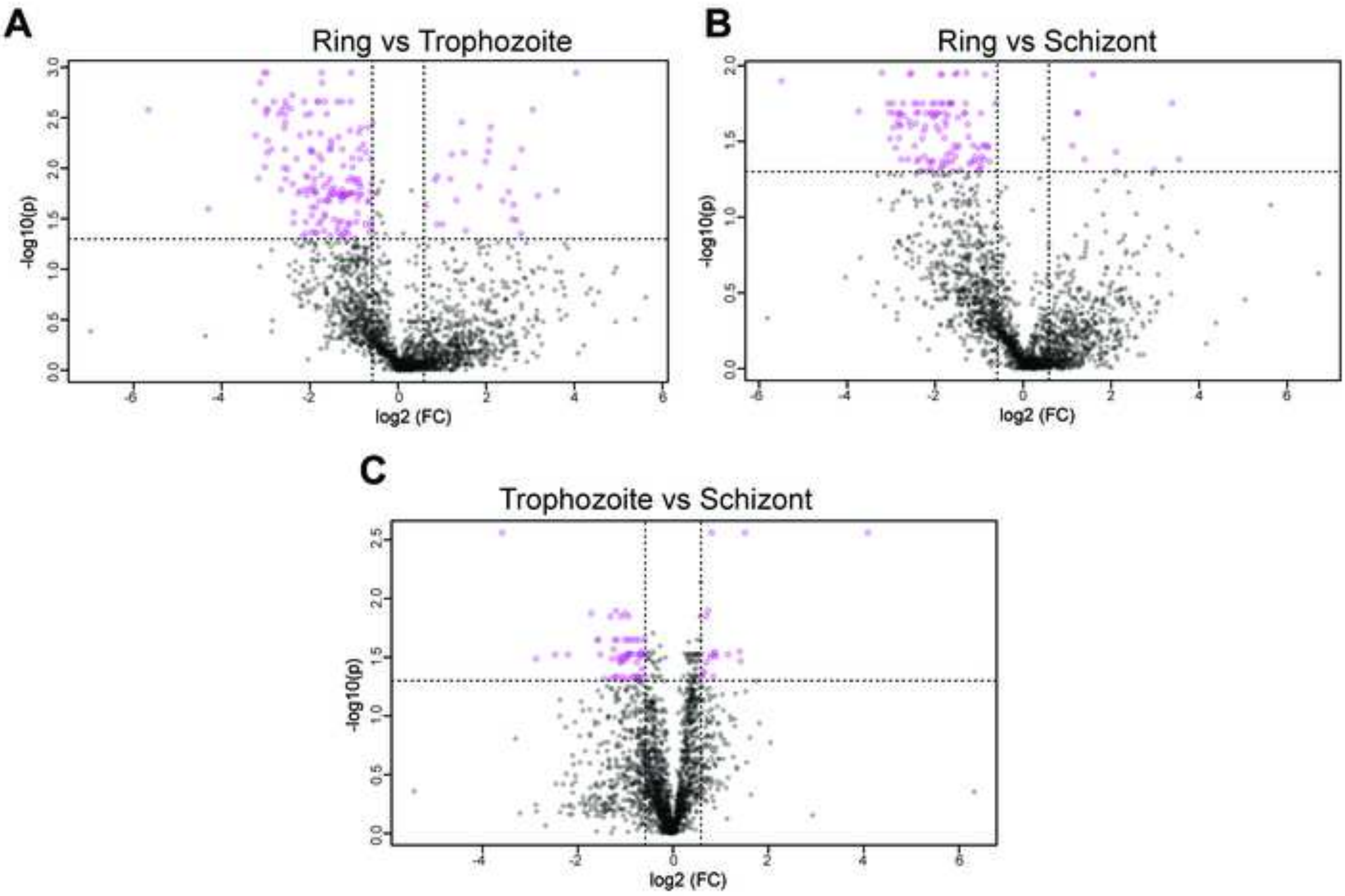

Supporting Information (Figures)

Click here to access/download
Supplementary Material
Supporting Information-cleaned.docx

Supplementary Data 1

Click here to access/download
Supplementary Material
Supplementary_data_sheet_1_fixed.csv

Supplementary Data 2

Click here to access/download

Supplementary Material

Supplementary_data_sheet_2-fixed.csv

Supplementary Data 3

Click here to access/download
Supplementary Material
Supplementary_data_sheet_3-fixed.csv

Supplementary Data 4

Click here to access/download

Supplementary Material

Supplementary_data_sheet_4-experiment-1-relative-abundance.csv

Supplementary Data 5

Click here to access/download

Supplementary Material

Supplementary_data_sheet_5-experiment-2-relative-abundance.csv

Supplementary Data 6

Click here to access/download

Supplementary Material

Supplementary_data_sheet_6.csv

Supplementary Data 7

Click here to access/download

Supplementary Material

Supplementary_data_sheet_7-experiment-3-relative-abundance.csv

Supplementary Data 8

Click here to access/download
Supplementary Material
Supplementary_data_sheet_8-fixed.csv

Supplementary Data 9

Click here to access/download

Supplementary Material

Supplementary_data_sheet_9-art-res-relative-abundance.csv

Click here to access/download
Supplementary Material
Supplementary_data_sheet_10-art-res-enrichment.csv

Supplementary Data 11

Click here to access/download
Supplementary Material
Supplementary_data_sheet_11.csv

Responses to Reviewer Comments
Click here to access/download;Personal Cover;Addressing-reviewers-comments-DIA-manuscript.docx

Subject: GIGA-D-21-00227 Response to Reviewers

Reviewer #1.

1. The reviewer has asked for how this DDA library performs compared to other DIA searching strategies, including using options for (1) spectral library prediction and (2) library-free searches? Can they demonstrate benefits, either in terms of coverage or detecting lower abundance proteins?

   Response: We thank the reviewer for the comment and have performed a DirectDIA search (library-free search) using our dataset (Experiment 1 comparing the distinct stages of the red blood cell stage infection) and found that spectral-library searches resulted in significantly greater number of proteotypic peptides compared to DirectDIA. Furthermore, this finding is consistent with previous reports that compared these types of approaches, and references have been included. This additional analysis is added to the results section, lines 204-211.

2. Can the Venn diagrams in Fig1C, Fig1D, Fig2C be proportional as formatted in the Venn in Fig1A?

   Response: The Figures have been modified as per suggestion.

3. I was not able to access the data in PDX027241 as no dataset was available when I used the reviewer login - can the authors double check and verify this?

   Response: The data have now been made publicly available, so the reviewers should now be able to access it.

4. After downloading supplementary data tables, I noted that there were often several .csv files for the same supplementary data table number. Are these intended to be separate sheets in a single excel? The descriptions of the SD are very short, and extending the supplementary data table descriptions on pg21.

   Response: We thank the reviewer for the comment, and we have modified the csv files to combine a number of supplementary dataset and have added detailed description for each of the supplementary data sheets in pg 21, lines 572-606. The supplementary datasheet numbers have also been modified and this has been updated within the manuscript.

5. In SD1 can the protein IDs be tidied up to separate protein IDS and gene names and a column added with cleaned UniProt/PlasmoDB ascensions?

   Response: This has been modified as per suggestion.

6. SD2 "Supplementary_data_sheet_2-non-seen" doesn't appear to have the same column formats as all the other tables for SD2?

   Response: This has been modified as per suggestion.

7. Can the SD4 sheets be combined into a single table for ease of reference?

Response: We thank the reviewer for the comment, however due to journal requirements, we are unable to combine into a single sheet, as they have to be individual csv files for access of data to those interested in reproducing our findings.

8. Can the protein IDs in each GO cluster be added to the SD6 tables for each GO term?

   Response: This has been modified as per suggestion.

Reviewer #2.

1. The authors did not consider in the introduction and the discussion is that there are different types of mass spectrometers and their fragmentation pattern has to be noted. DIA methods were first introduced for TOF instruments and using Orbitrap mass spectrometers provides different challenges in fragmentation and dwell times. Please add more in detail information about this topic to the manuscript.

   Response: We thank the reviewer for the comment and we have added a paragraph in the discussion addressing their points in lines 374-386.

Reviewer #3.

1. Please provide details of instrumentation and DIA analysis (fragment ions resolution etc.)

   Response: We thank the reviewer for the comment and we have modified the methods section as requested, please see lines 494-510.
