# Supplementary figures and images for: A new mass spectral library for high-coverage and reproducible analysis of the Plasmodium falciparum–infected red blood cell proteome
Source: Gigascience. 2022 Mar 7;11:giac008. doi: 10.1093/gigascience/giac008 (PMC8900498; doi:10.1093/gigascience/giac008)

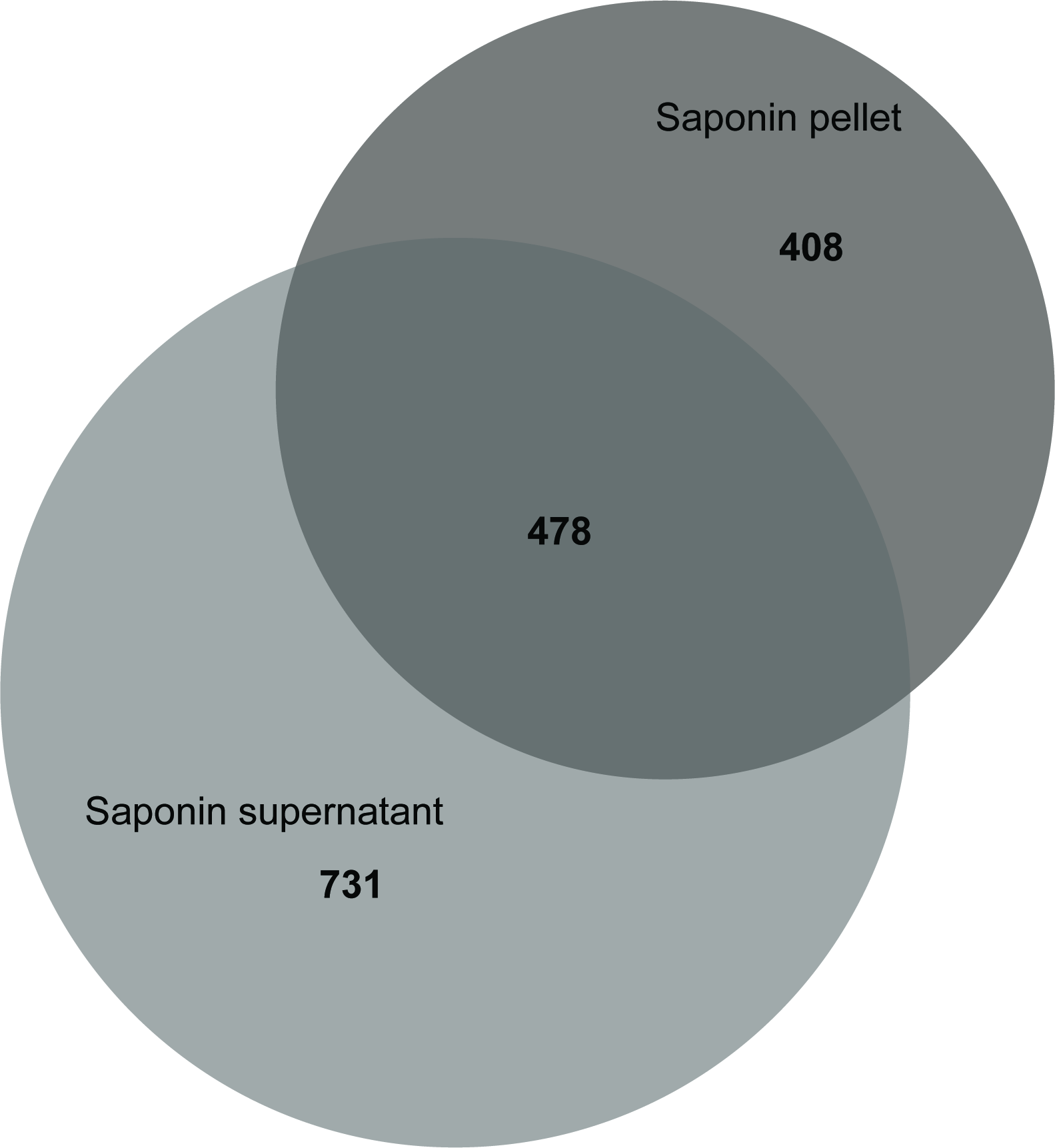

Supplement: giac008_Supplemental_Files [file giac008_supplemental_files.zip › Supp_Figure_1_venndigram_human_solubleVSinsoluble-V1.tif]

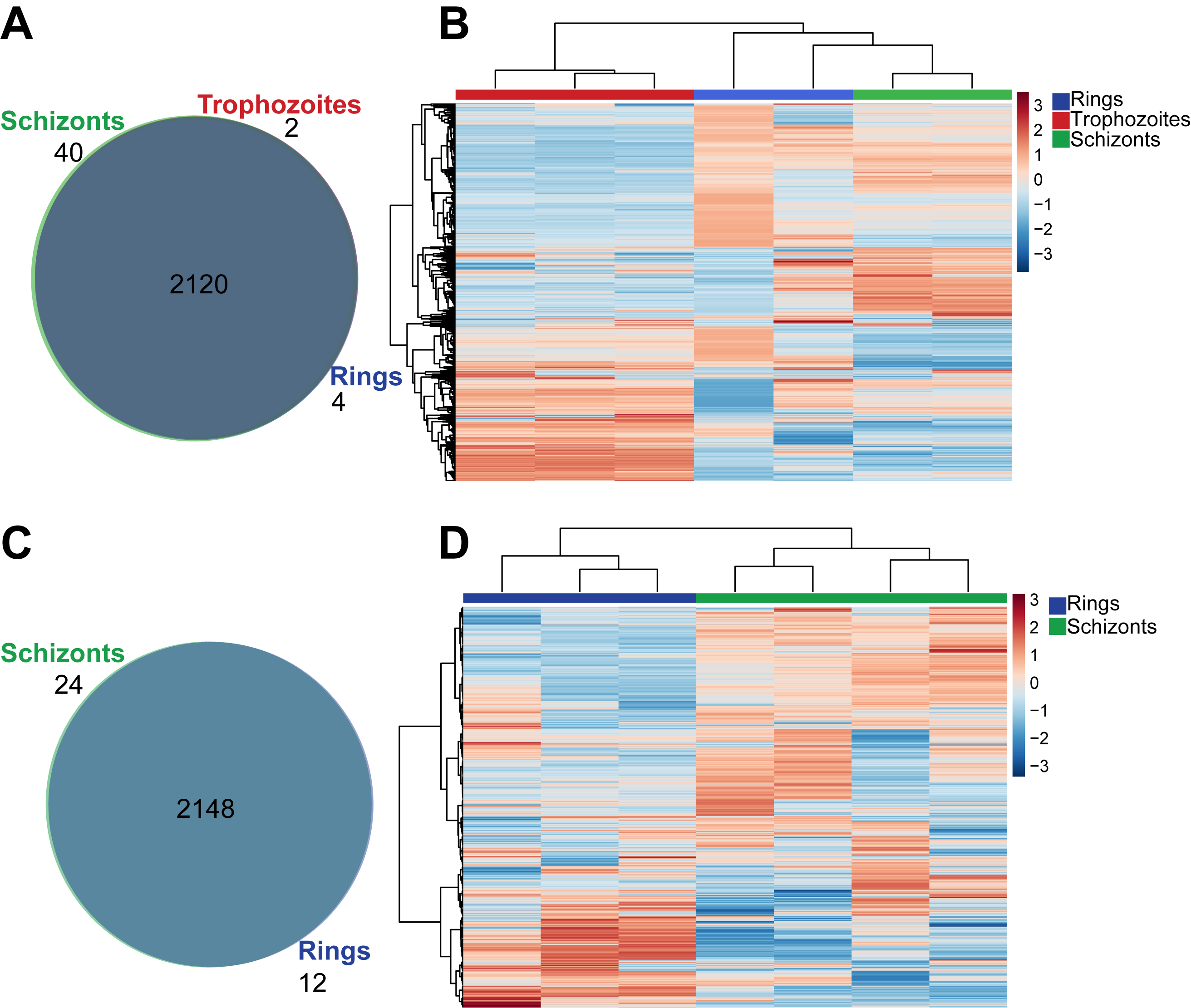

Supplement: giac008_Supplemental_Files [file giac008_supplemental_files.zip › Supp_Figure_2_heatmap_venndigram_DIA_exp_1_exp_2-v2.tif]

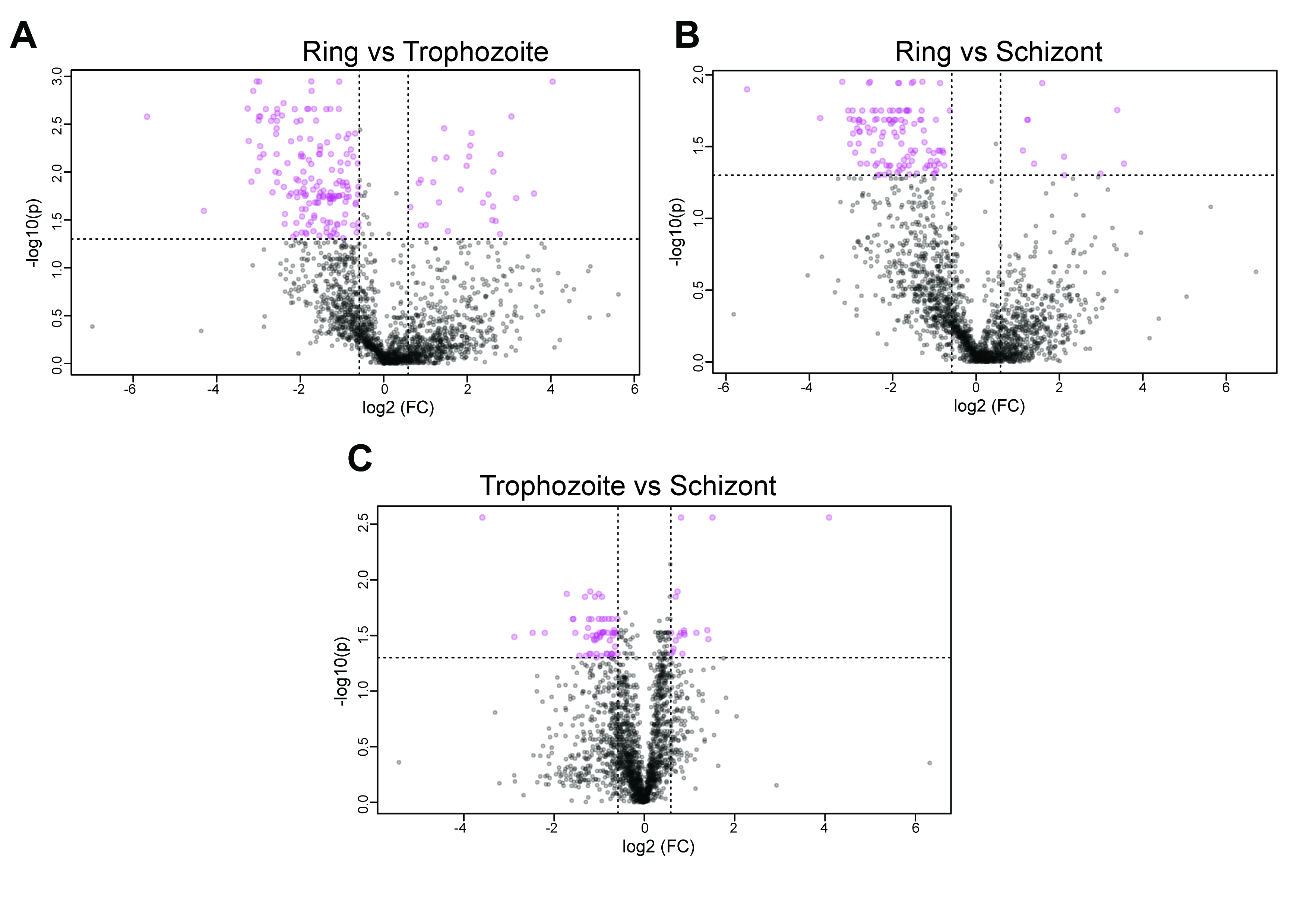

Supplement: giac008_Supplemental_Files [file giac008_supplemental_files.zip › Supp_Figure_3.tif]
