# Supplementary material for: A new mass spectral library for high-coverage and reproducible analysis of the Plasmodium falciparum–infected red blood cell proteome
Source: Gigascience. 2022 Mar 7;11:giac008. doi: 10.1093/gigascience/giac008 (PMC8900498; doi:10.1093/gigascience/giac008)
Supplement: giac008_Response_to_Reviewer_Comments_Revision_1 [file giac008_response_to_reviewer_comments_revision_1.pdf]

Author's Response To Reviewer Comments

Close

We thank the editor and reviewers for a thorough and helpful review of this manuscript. The suggested edits have been added to the manuscript and a point-by-point response to each reviewer comment is below:

Reviewer #1.
1. The reviewer has asked for how this DDA library performs compared to other DIA searching strategies, including using options for (1) spectral library prediction and (2) library-free searches? Can they demonstrate benefits, either in terms of coverage or detecting lower abundance proteins?

Response: We thank the reviewer for the comment and have performed a DirectDIA search (library-free search) using our dataset (Experiment 1 comparing the distinct stages of the red blood cell stage infection) and found that spectral-library searches resulted in significantly greater number of proteotypic peptides compared to DirectDIA. Furthermore, this finding is consistent with previous reports that compared these types of approaches, and references have been included. This additional analysis is added to the results section, lines 204-211.

2. Can the Venn diagrams in Fig1C, Fig1D, Fig2C be proportional as formatted in the Venn in Fig1A?

Response: The Figures have been modified as per suggestion.

3. I was not able to access the data in PDX027241 as no dataset was available when I used the reviewer login - can the authors double check and verify this?

Response: The data have now been made publicly available, so the reviewers should now be able to access it.

4. After downloading supplementary data tables, I noted that there were often several .csv files for the same supplementary data table number. Are these intended to be separate sheets in a single excel? The descriptions of the SD are very short, and extending the supplementary data table descriptions on pg21.
Response: We thank the reviewer for the comment, and we have modified the csv files to combine a number of supplementary dataset and have added detailed description for each of the supplementary data sheets in pg 21, lines 572-606. The supplementary datasheet numbers have also been modified and this has been updated within the manuscript.

5. In SD1 can the protein IDs be tidied up to separate protein IDS and gene names and a column added with cleaned UniProt/PlasmoDB ascensions?

Response: This has been modified as per suggestion.

6. SD2 "Supplementary_data_sheet_2-non-seen" doesn't appear to have the same column formats as all the other tables for SD2?

Response: This has been modified as per suggestion.

7. Can the SD4 sheets be combined into a single table for ease of reference?

Response: We thank the reviewer for the comment, however due to journal requirements, we are unable to combine into a single sheet, as they have to be individual csv files for access of data to those interested in reproducing our findings.

8. Can the protein IDs in each GO cluster be added to the SD6 tables for each GO term?

Response: This has been modified as per suggestion.

Reviewer #2.
1. The authors did not consider in the introduction and the discussion is that there are different types of mass spectrometers and their fragmentation pattern has to be noted. DIA methods were first introduced for TOF instruments and using Orbitrap mass spectrometers provides different challenges in fragmentation and dwell times. Please add more in detail information about this topic to the manuscript.

Response: We thank the reviewer for the comment and we have added a paragraph in the discussion addressing their points in lines 374-386.

Reviewer #3.
1. Please provide details of instrumentation and DIA analysis (fragment ions resolution etc.)

Response: We thank the reviewer for the comment and we have modified the methods section as requested, please see lines 494-510.

Close
