# Supplementary material for: A new mass spectral library for high-coverage and reproducible analysis of the Plasmodium falciparum–infected red blood cell proteome
Source: Gigascience. 2022 Mar 7;11:giac008. doi: 10.1093/gigascience/giac008 (PMC8900498; doi:10.1093/gigascience/giac008)
Supplement: giac008_Reviewer_1_Report_Original_Submission — Samantha Emery -- 9/17/2021 Reviewed [file giac008_reviewer_1_report_original_submission.pdf]

Reviewer Report

Title: A new mass spectral library for high-coverage and reproducible analysis of the Plasmodium falciparum-infected red blood cell proteome

Version: Original Submission **Date:** 9/17/2021

Reviewer name: Samantha Emery

Reviewer Comments to Author:

The authors have provided a very detailed and clearly communicated manuscript regarding their proteomic data. It was easy to read, and easy to understand the overlaps and unique aspects of their data compared to data in the scholarship and on PlasmoDB. It was also very pleasing to see the authors provide comprehensive and detailed supplementary information, ensuring transparency of their analysis. Overall I think this will be a great proteome resource, and it will be excellent to see that the data will be made available on PlasmoDB.

I think the manuscript is in great shape, but I have two questions for the authors and their spectral library. Have the authors explored how their DDA library performs compared to other DIA searching strategies, including using options for (1) spectral library prediction and (2) library-free searches? Can they demonstrate benefits, either in terms of coverage or detecting lower abundance proteins? Considering that the authors have gone to such depths (no pun intended) to ensure they have delivered a comprehensive spectral library, it would be interesting to compare if their DDA spectral library outperforms these methods. In addition, I would be curious to know whether library-free options for searching their DIA data could still provide good depth and quantitation, particularly as we are seeing DIA analysis tools improve with more library-free options available.

Figures, minor comment

If possible, can the Venn diagrams in Fig1C, Fig1D, Fig2C be proportional as formatted in the Venn in Fig1A?

Supplementary Data, comments

The authors have done an excellent job providing data, and documentation of the data - including inputs and outputs of figures.

The only issue I had with the supplementary data was I was not able to access the data in PDX027241 as no dataset was available when I used the reviewer login - can the authors double check and verify this? I noted the same reviewer username was provided (mailto:reviewer_pxd027301@ebi.ac.uk) but two unique identifiers were mentioned.

After downloading supplementary data tables, I noted that there were often several .csv files for the same supplementary data table number. Are these intended to be separate sheets in a single excel? The descriptions of the SD are very short, and extending the supplementary data table descriptions on pg21 (or providing a word document explaination for of the sheets as per "csv files for figures" supps) is required for those who wish to dig deeper into the data. Where possible, it would be great to consolidate information to decrease the number of sheets associated with each SD file.

I have added some additional comments below on the individual SDs

1. In SD1 can the protein IDs be tidied up to separate protein IDS and gene names and a column added with cleaned UniProt/PlasmoDB ascensions?
2. SD2 "Supplementary_data_sheet_2-non-seen" doesn't appear to have the same column formats as all the other tables for SD2?
3. Can the SD4 sheets be combined into a single table for ease of reference?
4. Can the protein IDs in each GO cluster be added to the SD6 tables for each GO term?

Methods

Are the methods appropriate to the aims of the study, are they well described, and are necessary controls included? Choose an item.

Conclusions

Are the conclusions adequately supported by the data shown? Choose an item.

Reporting Standards

Does the manuscript adhere to the journal's guidelines on minimum standards of reporting? Choose an item.

Choose an item.

Statistics

Are you able to assess all statistics in the manuscript, including the appropriateness of statistical tests used? Choose an item.

Quality of Written English

Please indicate the quality of language in the manuscript: Choose an item.

Declaration of Competing Interests

Please complete a declaration of competing interests, considering the following questions:

- Have you in the past five years received reimbursements, fees, funding, or salary from an organisation that may in any way gain or lose financially from the publication of this manuscript, either now or in the future?
- Do you hold any stocks or shares in an organisation that may in any way gain or lose financially from the publication of this manuscript, either now or in the future?
- Do you hold or are you currently applying for any patents relating to the content of the manuscript?

- Have you received reimbursements, fees, funding, or salary from an organization that holds or has applied for patents relating to the content of the manuscript?
- Do you have any other financial competing interests?
- Do you have any non-financial competing interests in relation to this paper?

If you can answer no to all of the above, write 'I declare that I have no competing interests' below. If your reply is yes to any, please give details below.

I declare that I have no competing interests

I agree to the open peer review policy of the journal. I understand that my name will be included on my report to the authors and, if the manuscript is accepted for publication, my named report including any attachments I upload will be posted on the website along with the authors' responses. I agree for my report to be made available under an Open Access Creative Commons CC-BY license (http://creativecommons.org/licenses/by/4.0/). I understand that any comments which I do not wish to be included in my named report can be included as confidential comments to the editors, which will not be published.

Choose an item.

To further support our reviewers, we have joined with Publons, where you can gain additional credit to further highlight your hard work (see: https://publons.com/journal/530/gigascience). On publication of this paper, your review will be automatically added to Publons, you can then choose whether or not to claim your Publons credit. I understand this statement.

Yes Choose an item.
