# Supplementary material for: A new mass spectral library for high-coverage and reproducible analysis of the Plasmodium falciparum–infected red blood cell proteome
Source: Gigascience. 2022 Mar 7;11:giac008. doi: 10.1093/gigascience/giac008 (PMC8900498; doi:10.1093/gigascience/giac008)
Supplement: giac008_Reviewer_2_Report_Original_Submission — Guenther Kahlert -- 9/21/2021 Reviewed [file giac008_reviewer_2_report_original_submission.pdf]

Reviewer Report

Title: A new mass spectral library for high-coverage and reproducible analysis of the Plasmodium falciparum-infected red blood cell proteome

Version: Original Submission **Date:** 9/21/2021

Reviewer name: Guenther Kahlert

Reviewer Comments to Author:

Siddiqui et al presents new DIA repository library of Plasmodium falciparum in this manuscript, created on-the-fly without using synthetic peptides but solely using the parasitic asexual red blood cell stages. The aim of this survey study is achieved by the authors, covering 87% of the P. falciparum proteome using DDA mass spectrometry to create the presented DIA spectral library.
The by the authors presented DIA spectral library was created by elaborately purifying the parasite. Using their Orbitrap, MaxQuant, and Spectonaut pipeline has lead to a solid, reliable spectral library. And using mutants added biological reliability.
The only thing that the authors did not take into account in the introduction and the discussion is that there are different types of mass spectrometers and their fragmentation pattern has to be noted. DIA methods were first introduced for TOF instruments and using Orbitrap mass spectrometers provides different challenges in fragmentation and dwell times.
Please add more in detail information about this topic to the manuscript.
The statistics and the plots of this manuscript are well done and adequate to the survey. I especially value the volcano plots and the heat maps that show the difference between the red blood cell stages of of the parasite.

Methods

Are the methods appropriate to the aims of the study, are they well described, and are necessary controls included? Choose an item.

Conclusions

Are the conclusions adequately supported by the data shown? Choose an item.

Reporting Standards

Does the manuscript adhere to the journal's guidelines on minimum standards of reporting? Choose an item.

Choose an item.

Statistics

Are you able to assess all statistics in the manuscript, including the appropriateness of statistical tests used? Choose an item.

Quality of Written English

Please indicate the quality of language in the manuscript: Choose an item.

Declaration of Competing Interests

Please complete a declaration of competing interests, considering the following questions:

- Have you in the past five years received reimbursements, fees, funding, or salary from an organisation that may in any way gain or lose financially from the publication of this manuscript, either now or in the future?
- Do you hold any stocks or shares in an organisation that may in any way gain or lose financially from the publication of this manuscript, either now or in the future?
- Do you hold or are you currently applying for any patents relating to the content of the manuscript?
- Have you received reimbursements, fees, funding, or salary from an organization that holds or has applied for patents relating to the content of the manuscript?
- Do you have any other financial competing interests?
- Do you have any non-financial competing interests in relation to this paper?

If you can answer no to all of the above, write 'I declare that I have no competing interests' below. If your reply is yes to any, please give details below.

I declare that I have no competing interests.

I agree to the open peer review policy of the journal. I understand that my name will be included on my report to the authors and, if the manuscript is accepted for publication, my named report including any attachments I upload will be posted on the website along with the authors' responses. I agree for my report to be made available under an Open Access Creative Commons CC-BY license (http://creativecommons.org/licenses/by/4.0/). I understand that any comments which I do not wish to be included in my named report can be included as confidential comments to the editors, which will not be published.

Choose an item.

To further support our reviewers, we have joined with Publons, where you can gain additional credit to further highlight your hard work (see: https://publons.com/journal/530/gigascience). On publication of this paper, your review will be automatically added to Publons, you can then choose whether or not to claim your Publons credit. I understand this statement.

Yes Choose an item.
